# Stereoselective Anomeric Phosphorylation under Modified Mitsunobu Reaction Conditions

**DOI:** 10.1021/acs.joc.5c01760

**Published:** 2025-09-24

**Authors:** Alessandro Monti, Biswajit Sarkar, Alla Zamyatina

**Affiliations:** Department of Natural Sciences and Sustainable Resources, Institute of Organic Chemistry, 27270 BOKU University, Muthgasse 18, Vienna A-1190, Austria

## Abstract

Glycosyl phosphates are vital components of various biomolecules and serve as intermediates in the biosynthesis of substrates for glycosyltransferases such as nucleotide-activated sugars and dolichol-phosphate sugars. Their limited availability underscores the importance of stereoselective chemical synthesis of glycosyl phosphates with a defined anomeric configuration. A particular challenge lies in the generation of glycosyl phosphates with an anomeric configuration opposite to that of the starting lactol, as exemplified by l/d-*glycero*-β-d-*manno*-heptosyl phosphate-precursors in the synthesis of the pathogen-associated molecular pattern ADP-heptose, which triggers innate immune signaling through interaction with α-kinase-1 (ALPK1). The modification of the Mitsunobu reaction enabled stereospecific anomeric phosphorylation and efficient synthesis of challenging β-heptosyl phosphates. Mechanistic studies using NMR spectroscopy guided the differentiation between two possible reaction pathways, proceeding either with the inversion or with retention of the anomeric configuration. The identification of (diglycosyloxy)­phosphoranes as reactive species enabled the development of specialized reaction protocols that facilitate the stereoselective synthesis of glycosyl phosphates with inversion of the anomeric configuration via an SN2 mechanism. The use of auxiliary reagents altered the reaction pathway, leading instead to the formation of glycosyl phosphates with the retention of the anomeric configuration.

## Graphical abstract

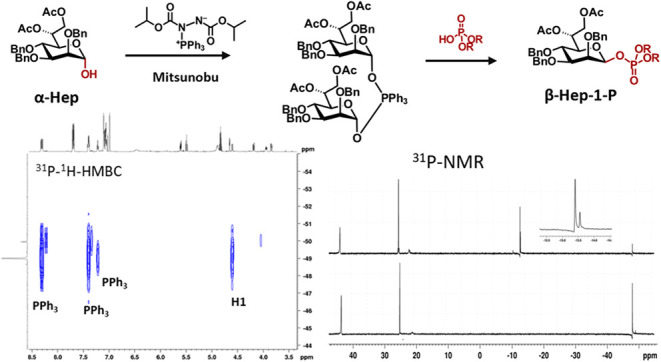

## Introduction

Glycosyl phosphates are essential components of diverse biomolecules and biopolymers such as phosphorylated bacterial and parasitic glycans and are also intermediates in the biosynthesis of nucleotide-activated sugars, which act as substrates for glycosyltransferases.^1^ Glycosyltransferases are key enzymes involved in glycosylation reactions that are essential for almost all vital biological processes, while the dysregulation of protein glycosylation is strongly associated with the onset and progression of various congenital and acquired diseases, including cancers.^2^ Understanding the structural and chemical mechanisms of glycosyltransferases is crucial for exploring their functions and potential applications in synthetic biology and drug discovery.^3^ Most glycosyltransferases exhibit strict stereospecificity, with a few exceptions capable of processing both α- and β-anomers of sugar nucleotides.^1,4^ One of the obstacles in using glycosyltransferases to study the functional role of glycosylation in biological systems is the limited availability of their substrates: nucleotide-activated sugars and dolichol-phosphate sugars with a specific anomeric configuration. This underscores the importance of stereospecific synthetic methods for producing glycosyl phosphates, which serve as key precursors in the chemical or enzymatic synthesis of nucleotide-activated and dolichol-phosphate sugars.^5^

Indeed, both chemical and enzymatic approaches to the synthesis of sugar nucleotides and phosphoglycans commonly rely on chemically prepared glycosyl phosphates or their analogues with a specific anomeric configuration.^6^ While enzymatic methods for synthesizing anomeric sugar phosphates are accessible, the flexibility and versatility of chemical synthesis remain unparalleled. A variety of synthetic methods have been developed for the preparation of anomeric phosphates and phosphites to be used as glycosyl donors in chemical glycosylation reactions; however, stereoselectivity in anomeric phosphorylation is of less significance in this context.^7^

The most common approaches to the chemical synthesis of glycosyl phosphates can be divided into two main categories: (1) the anomerically activated sugar acts as the electrophilic moiety, which undergoes a nucleophilic displacement reaction by a phosphate anion (electrophilic glycosyl donors, such as glycosyl halides, trichloroacetimidates, orthoesters, activated 1-thioglycosides, and glycals are commonly used in combination with phosphate-derived glycosyl acceptors); (2) the anomeric hydroxyl group acts as a nucleophilic moiety, reacting with an activated phosphate or phosphite, which involves either the activation of the anomeric hydroxyl group through 1-*O*-lithiation, followed by its reaction with protected phosphoric anhydride or the coupling of the anomeric lactol with activated phosphates (e.g., phosphoryl chlorides in the presence of a base) or phosphite derivatives (e.g., phosphoramidites or imidazolyl phosphites).^8^ These methods provide good yields but show variable stereoselectivity, largely influenced by the stereoconfiguration of the sugar component, whereas the synthesis of glycosyl phosphates of the β-*manno* configuration presents a particularly challenging case.

The interest in the chemical synthesis of β-d-mannosyl*-* and l/d-*glycero*-β-d-*manno*-heptosyl phosphates dates back 20–25 years, driven by their putative biological activity and potential biomedical application. This includes the preparation of β-mannosyl-phosphodolichols, specifically β-d-mannosyl-phosphomycoketides—naturally occurring glycolipids found in the cell walls of *Mycobacteria* that act as potent antigens to activate T cells,^9^ and the Gram-negative bacterial metabolite ADP-l-*glycero-* and ADP-d-*glycero*-β-d-*manno*-heptose (ADP-Hep), a key substrate for heptosyltransferase in the LPS biosynthesis pathway.^10^ The recent discovery of the cytosolic kinase ALPK1 (α-kinase-1) as a pattern recognition receptor that directly detects bacterial ADP-Hep and triggers NF-κB signaling followed by proinflammatory innate immune responses has further spurred interest in this field.^11^ Smaller heptose-based bacterial metabolites such as HBP (β-heptose-1,7-bisphosphate) and β-heptose-1-phosphate are also recognized as important virulence factors and potential immunomodulators in the *Helicobacter pylori* infection.^12^

The significance and implication of the small molecule β-d-manno-heptose-1-phosphate and its derivatives in diseases with an immunopathological background are currently the subject of intensive research.^13^ Several critical diseases that depend on the ALPK1 signaling axis, such as inflammatory bowel disease,^14^ chronic *H. pylori* infections,^15^ and others, or those resulting from ALPK1 mutations, including the autosomal disorder ROSAH syndrome (characterized by retinal dystrophy, optic nerve edema, splenomegaly, anhidrosis, and migraine headache),^16^ may be amenable to therapeutic modulation using synthetic l/d-*glycero*-β-d-*manno*-heptose-1-phosphate-derived compounds, ADP-heptose, or structural analogues. A recent seminal study revealed that age-related intestinal changes trigger the systemic spread of ADP-Hep, which drives the expansion of preleukemic mutant hematopoietic cells, contributing to increased susceptibility to leukemia and immune-related disorders.^17^ In addition to ADP-heptose, CDP-heptose and UDP-heptose have also been shown to induce strong ALPK1-mediated proinflammatory signaling.^18^ Functional nucleotide-diphosphate-heptose biosynthetic enzymes appear to be widely distributed across bacterial, archaeal, and viral kingdoms, highlighting the importance of β-d-*manno*-heptoses and nucleotide-activated heptoses as cross-kingdom pathogen-associated molecular patterns capable of inducing ALPK1-dependent innate immunity.^18^

## Results and Discussion

### Phosphorylation Using Adjusted Mitsunobu Conditions

The most effective methodology for the stereocontrolled β-anomeric phosphorylation in the d-*manno*-series relies on the approach where the anomeric OH group serves as a nucleophile undergoing a reaction with an activated phosphodiester, e.g., diphenyl phosphoryl chloride.^19^ A more reactive equatorial lactol, formed through anomerization in the presence of a large excess of DMAP, reacts more readily with the electrophilic phosphate moiety than its more thermodynamically stable α-counterpart, leading to the preferential formation of the β-mannosyl phosphate. This approach has been successfully applied to the synthesis of important mannose- and heptose-containing biomolecules, where the kinetic conditions underlying β-stereoselectivity were achieved by limiting the amount of activated phosphate in the reaction mixture, allowing the newly formed β-lactol to react selectively with the chlorophosphate.^9b,10a,20^ Varying the protecting groups on the phosphorus atom, e.g., by using diallyl chlorophosphate, provided similar anomeric selectivity in the phosphorylation reaction and expanded the repertoire of functional groups compatible with the conditions of phosphate deblocking.^21^ Despite obvious advantages such as stereoselectivity, this approach has several limitations, as it requires the use of unstable chlorophosphates, which sometimes need to be prepared in situ, and relies on sophisticated reaction conditions, such as the use of a large excess of the base (DMAP) and a very slow dropwise addition of a solution of the unstable chlorophosphate, which are strictly stereoselectivity-determining.

The Mitsunobu reaction, in its original form, involves the reaction of a nucleophile, most commonly an alcohol, with a carboxylic acid, promoted by a reactive intermediate known as a betaine, which is generated from triphenylphosphine and a dialkyl azodicarboxylate.^22^ This transformation, proceeding with the inversion of the configuration, has attracted considerable attention in carbohydrate chemistry for the modification of primary and secondary hydroxyl groups. The classical Mitsunobu reaction at the anomeric position, however, represents a special case due to its challenging stereochemistry.^23^ In some cases, phosphoric acid diesters can also be used instead of a carboxylic acid, leading to the formation of phosphotriesters (hereafter termed *phosphorylation under modified Mitsunobu reaction conditions*). However, the stereochemistry and mechanism of this specific modification of the Mitsunobu reaction have not been clearly defined, especially since most reported cases have involved a primary hydroxyl group as the nucleophile^22,24^ and the use of an auxiliary reagent such as triethylamine.^25^ Adaptation of the Mitsunobu reaction for anomeric phosphorylation with the inversion of configuration has been reported by the groups of Vincent^26^ and Fujimoto^27^ for the synthesis of β-heptose-1,7-bisphosphate (HBP), as well as by us,^28^ albeit furnishing only moderate stereoselectivity and overall yields. Application of the Mitsunobu reaction conditions toward the synthesis of glycosyl phosphates from other sugars also appears to be challenging and inefficient,^29^ whereas its use for nonstereoselective phosphorylation of the primary hydroxyl group in pyranoses (e.g., C6-OH in ManNAc or C7-OH in d-*glycero-* d *-gluco-*Hep) affords the expected phosphoester linkage in high yield.^25^

Given the mildness of the Mitsunobu reaction conditions, essential for maintaining the stability of newly generated, intrinsically labile glycosyl phosphates, and the easy availability and chemical stability of protected phosphoric acids as reagents (used in place of unstable phosphoric acid chloroanhydrides, in line with the phosphate triester approach), we investigated the reaction mechanisms underlying anomeric phosphorylation under modified Mitsunobu conditions. Acetylated mannose lactol **1** (Scheme 1), which exhibits a high prevalence of the α-anomer (α/β = 9:1) and thus enables the straightforward control of reaction stereoselectivity, was used as the substrate for all initial experiments while also serving as a structural surrogate for the rare 7-carbon sugar d-*manno*-heptose.

**Scheme 1:**
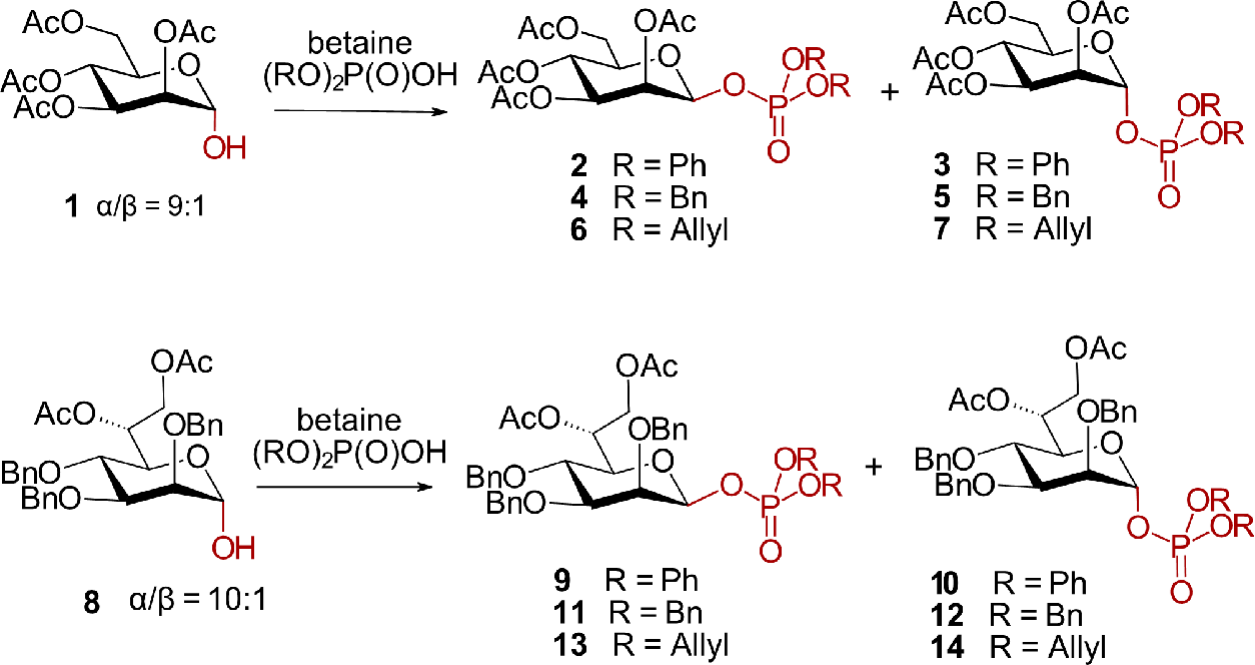
Phosphorylation of Man-lactol **1** and Hep-lactol **8** under Modified Mitsunobu Reaction Conditions

### Anomeric Phosphorylation under Mitsunobu Reaction Conditions Proceeds via Generation of (Di-glycosyloxy)­phosphoranes

The Mitsunobu reaction begins with the nucleophilic attack of a phosphine, typically triphenylphosphine, on the N=N double bond of an azodicarboxylate, commonly diethyl azodicarboxylate (DEAD) or diisopropylazodicaroxylate (DIAD). This initial step has been shown to be irreversible, resulting in the formation of a relatively stable betaine intermediate.^30^ The mechanism of the second step remains controversial and involves at least two competing pathways.^31^ As our initial goal was to determine the optimal reaction conditions, such as the nature of the phosphine and azodicarboxylate, the quantities of reagents, the choice of solvent, etc., we employed Man-lactol **1** as the nucleophilic- and dibenzyl phosphate as the acidic component to generate the known protected β-*manno*-1-(dibenzyl)­phosphates. As a more advanced objective, diphenyl- and diallylphosphoric acid derivatives were also used to synthesize a series of differently protected β-*manno*-1-phosphates **2**–**7** to investigate the influence of protecting groups at phosphorus (Scheme 1). Once the reaction conditions had been established and a thorough understanding of the reaction mechanism and kinetics had been gained, the anomeric phosphorylation of 2,3,4-tri-*O*-benzyl-d-*glycero*-d-*manno*-heptose **8** was subsequently studied by using three distinctly protected phosphoric acids. These newly established conditions allowed for anomeric phosphorylation with inversion of configuration and the predominant formation of β-configured heptosyl phosphates (**9, 11, and 13**), accompanied by minor amounts of the corresponding α-anomers (**10, 12, and 14**) (Scheme 1 and Table 1).

**Table 1:** Synthesis of β-Mannosyl Phosphates **2, 4, 6** and β-Heptosyl Phosphates **9, 11, 13** with Inversion of Anomeric Configuration^a^

			yield/product	
lactol	phosphate	temp.	β %	α %
**1**	(PhO)_2_P(O)OH	r.t.	68 (**2**)	22 (**3**)
**1**	(BnO)_2_P(O)OH	r.t.	65 (**4**)	21 (**5**)
**1**	(AllylO)_2_P(O)OH	r.t.	61 (**6**)	20 (**7**)
**8**	(PhO)_2_P(O)OH	–10 °C	71 (**9**)	26 (**10**)
**8**	(BnO)_2_P(O)OH	–10 °C	71 (**11**)	12 (**12**)
**8**	(AllylO)_2_P(O)OH	–10 °C	64 (**13**)	21 (**14**)

a Reaction conditions are indicated in Figure 4A,B and in the Experimental Section; reaction protocol *MIP-inversion*.

Following the original procedure^22^ consisting of combining a nucleophile, mannose lactol **1**, with an acidic component (dibenzyl phosphate), triphenylphosphine and DIAD resulted in no conversion (Table S1, entries 1 and 2). Similarly, when PPh_3_, DIAD, and dibenzyl phosphate were premixed in a solvent (THF, DCM, or toluene) prior to supplementing with lactol **1**, no product formation was observed (Table S1, entries 5, 8, and 14). It thus became clear that the synthesis of glycosyl phosphates may benefit from sophisticated protocols^32^ that strategically alter the order of reagent addition. Specifically, we found that the formation of the betaine, generated via a Michael-type nucleophilic attack of triphenylphosphine on diisopropyl azodicarboxylate,^30a^ must precede the addition of any other reaction components (Table S1). Of the five solvents tested (CH_3_CN, THF, DMF, toluene, and DCM), toluene showed a slight, but not significant, improvement in reaction selectivity and yield. The use of triethylamine as an additive^26–29^ increased the overall yield up to 80%, however, at the expense of stereoselectivity, as a substantial proportion of the α-configured product was formed without inversion of configuration (Table S1).

To gain deeper insight into the reaction kinetics and to explore strategies for achieving stereoselective anomeric phosphorylation under Mitsunobu conditions, we monitored the reaction progress in situ by ^31^P NMR, performing the phosphorylation directly in the NMR tube using toluene-d_8_ as the solvent. Premixing PPh_3_ (1 equiv) and DIAD (1 equiv) in toluene-d_8_ resulted in the formation of two species detectable in the ^31^P NMR spectrum: betaine **15** (^31^P NMR, δ: + 42 ppm) and triphenylphosphine oxide (TPPO) **16** (^31^P NMR, δ: + 24 ppm) (Figure 1A), with ^31^P NMR chemical shifts consistent with previously reported data.^31b,33^ The observation of the TPPO signal before the addition of lactol **1** indicates that betaine undergoes partial hydrolysis due to residual water in the deuterated solvent, requiring an adjustment of its quantity to ensure accurate stoichiometry. Upon the addition of lactol **1** (1 equiv), a new ^31^P NMR signal appeared at δ: −49 ppm, consistent with the formation of (dimannosyloxy)­phosphorane **17**,^31c,34^ while betaine **15** remained detectable in the mixture. The relative intensities of betaine **15** and phosphorane **17** were approximately 1:1. Increasing the amount of lactol **1** to 2 equiv., while maintaining betaine at 1 equiv., led to the complete consumption of both components and full conversion of **1** to phosphorane **17** (Figure 1B).

**Figure 1:**
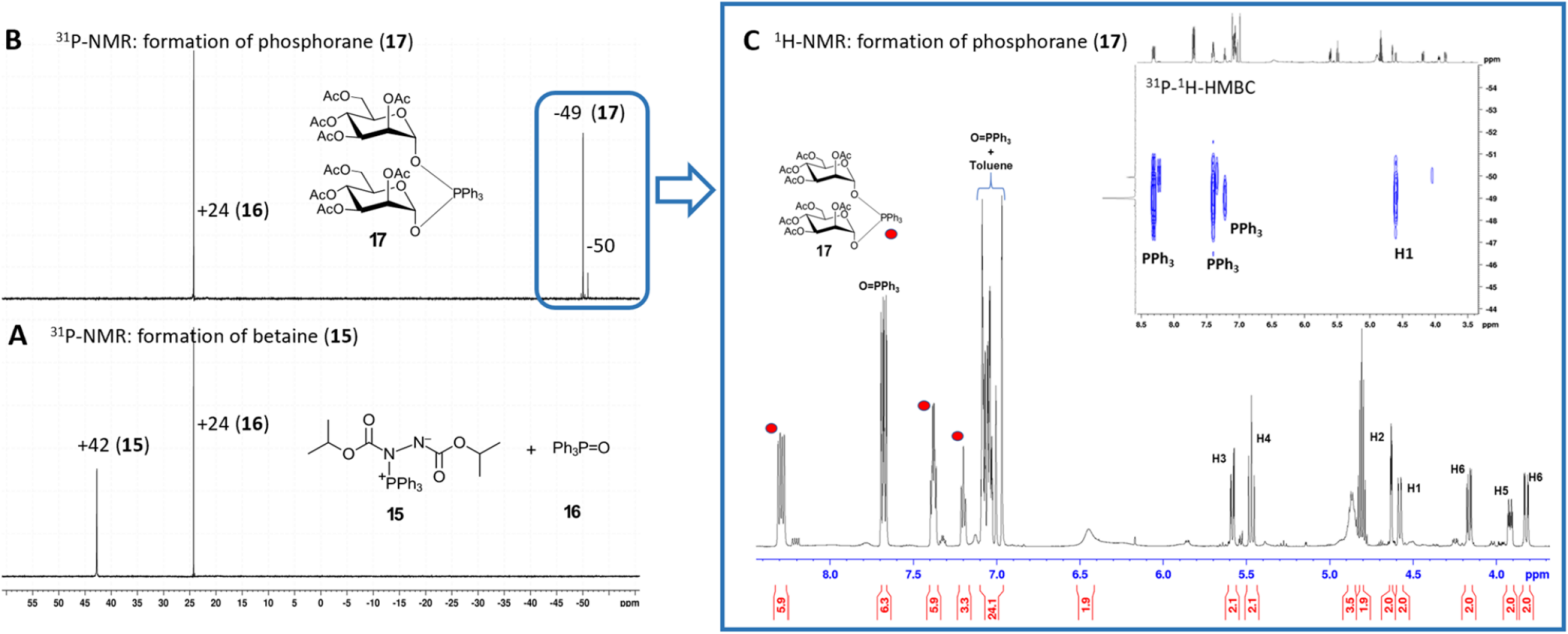
Formation of (dimannosyloxy)­triphenylphosphorane **17** from Man-lactol **1** and betaine **15**. (A) ^31^P NMR spectrum demonstrating the formation of betaine **15**; (B) ^31^P NMR spectrum showing the formation of **17** from betaine and Man-lactol **1**; and (C) ^1^H NMR and ^31^P–^1^H-HMBC NMR spectra of **17**.

The stoichiometry of this transformation, in which 1 mol of lactol **1** consumes only 0.5 mol of betaine **15**, as well as analysis of the ^1^H NMR-, ^31^P–^1^H-HMBC, and ^13^C–^1^H-HSQC NMR spectra (Figures 1C and S2), unambiguously confirmed the proposed structure of **17** as di­(2,3,4,6-tetra-*O*-acetyl-α-d-*manno*-pyranosyl)-triphenylphosphorane (^31^P NMR, δ: −49 ppm). Integration of the ^1^H NMR signals also supported the reaction stoichiometry, indicating two sugar moieties per the PPh_3_ unit, consistent with the structure of **17** (Figures S1 and S2). The 1,2-*trans*- configuration (α-d-*manno*) was confirmed by analysis of the heteronuclear coupling constant *J*_C1,H1_ = 168 Hz.^35^ A small signal δ = −50 ppm (about 10%) was assigned to the β-configured counterpart of **17** generated from a minor proportion of β-Man-lactol.

The reaction of tribenzylated d-*glycero*-d-*manno*-heptose lactol **8** with preformed betaine **15** (Figure 2A) was similarly carried out in the NMR tube using toluene-d_8_ as the solvent, while each step of reagent addition was monitored by ^31^P NMR analysis. When equimolar amounts of betaine **15** (1 equiv) and lactol **8** (1 equiv) were used, the ^31^P NMR resonance of betaine **15** (^31^P NMR, δ: + 42 ppm) persisted after the addition of lactol **8**. However, the addition of a second equivalent of **8** led to the immediate and full consumption of betaine accompanied by the appearance of a new resonance at ^31^P NMR, δ = −50 ppm, consistent with the formation of phosphorane **18** (Figure 2B). The structure of this new species was assigned as [di­(d-*glycero*-α-d-*manno*-heptosyloxy)]­phosphorane using ^31^P–^1^H-HMBC and ^13^C–^1^H-HSQC analyses (Figures 2C, S3, and S4). The anomeric configuration of **18** was confirmed by the analysis of the heteronuclear coupling constant *J*_C1,H1_ = 168 Hz, consistent with the α-d-*manno* configuration.^35^

**Figure 2:**
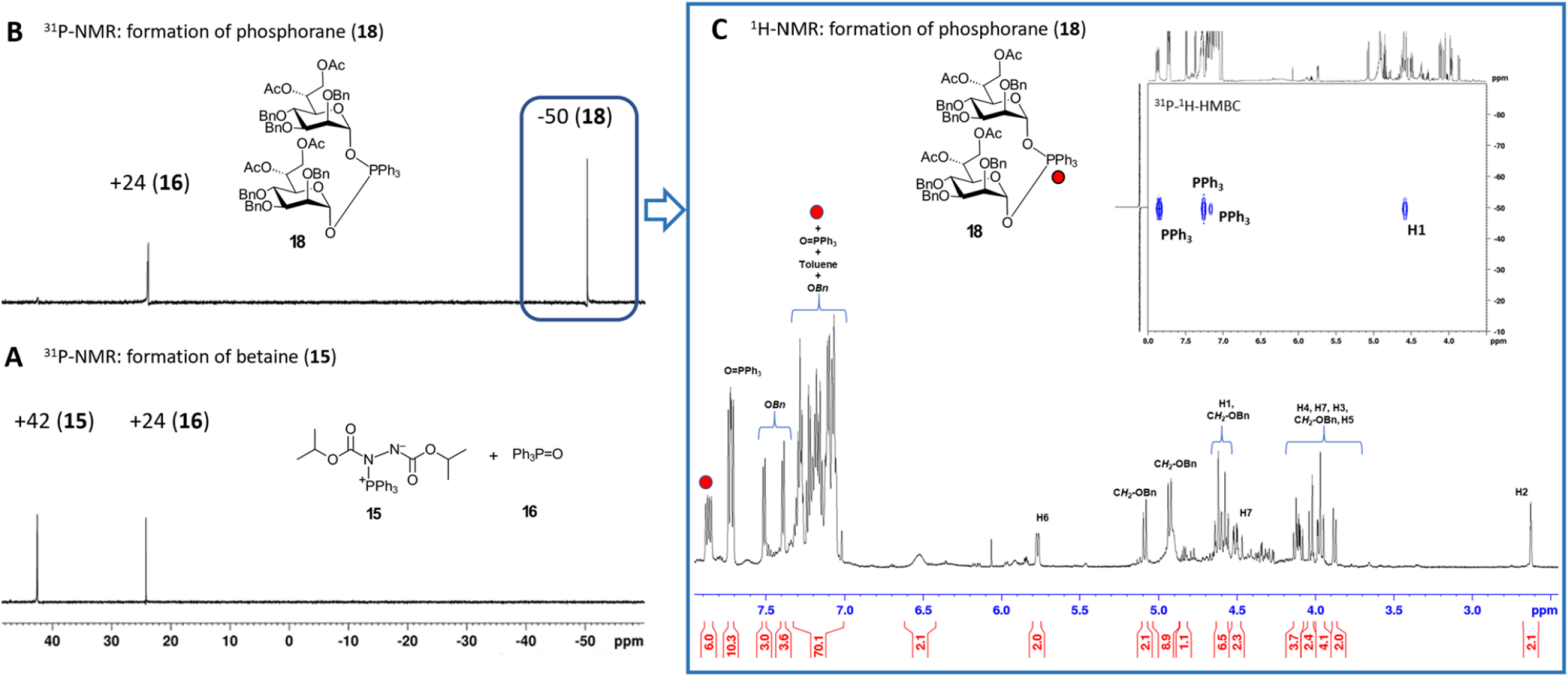
Formation of (diheptosyloxy)­triphenylphosphorane **18** from Hep-lactol **8** and betaine **15**. (A) ^31^P NMR spectrum demonstrating the formation of betaine **15**; (B) ^31^P NMR spectrum showing the formation of **18** from betaine and Hep-lactol **8**; and (C) ^1^H NMR and ^31^P–^1^H-HMBC NMR spectra of **18**.

The stoichiometry of this reaction was largely similar to that of the phosphorylation of tri-*O*-acetylated α-Man-lactol **1**, with the key difference being the reduced stability of (diheptosyloxy)­phosphorane **18**, attributed to the increased reactivity of the anomeric center, resulting from the presence of electron-donating benzyl groups. The assignment of signals and calculation of the reaction stoichiometry based on the integration of signals in the ^1^H NMR spectrum proved significantly more challenging due to an extensive signal overlap in the aromatic region (arising from the benzyl groups of the heptose, the phenyl groups of **18** and TPPO, and the residual toluene signal; Figures S3 and S4 and Table S2). Additional complexity arose from multiple overlapping signals in the carbohydrate region caused by minor resonances corresponding to the β-configured counterpart of **18** (∼5%) and a mixture of α/β-heptose (∼5%) formed as a result of partial hydrolysis of **18**.

Considering the failure to generate and characterize phosphoranes **17** and **18** in solvents other than toluene, and the known influence of solvent polarity on reaction rates,^36^ we examined the stability of phosphorane **17** in various deuterated solvents using ^31^P NMR spectroscopy. The ^31^P NMR peak at δ: −49 ppm, corresponding to (dimannosyloxy)­phosphorane **17**, was still detectable 10 min after its initial appearance in toluene-d_8_, but not in CD_2_Cl_2_, THF-d_8_, or CD_3_CN, where **17** decomposed within 5 min of formation (Figure S5). In toluene-d_8_, however, **17** remained sufficiently stable for at least 3 h, gradually decomposing into lactol **1** and TPPO within 6 h, as shown by the ^31^P NMR analysis (Figure S6). These results clearly demonstrate that for anomeric modifications using Mitsunobu reaction conditions, nonpolar solvents such as toluene are optimal due to the significantly prolonged half-life of the reactive anomeric (diglycosyloxy)­phosphorane species. Due to the “arming” effect of the benzyl protecting group, the anomeric center in **18** is more reactive than in **17**, leading to a shorter half-life of (diheptosyloxy)­phosphorane **18**, which begins to decompose as early as 1 h after its initial detection in the ^31^P NMR spectrum, with its proportion dropping to 20% within 4 h (Figure S7).

### Stereoselectivity of Anomeric Phosphorylation under Modified Mitsunobu Reaction Conditions: Synthesis of Glycosyl Phosphates with Inversion of Anomeric Configuration

The confirmed formation of phosphoranes **17** and **18** as the key intermediates aligns with the classical Mitsunobu reaction mechanism, implying that the products of the subsequent phosphorylation step must undergo an inversion of the anomeric configuration. However, in all of the phosphorylation attempts, where the formation of **17** was confirmed by ^31^P NMR, both the stereoselectivity and the extent of conversion remained low. The reaction was initiated by preforming betaine **15** (^31^P NMR, δ: + 42 ppm) using an excess of PPh_3_ (3 equiv) and DIAD (3 equiv) in toluene-d_8_ (Figure 3A). Subsequent addition of the nucleophilic component, an α-configured lactol **1** (1 equiv), resulted in the formation of phosphorane **17** (^31^P NMR, δ: −49 ppm), while betaine **15** remained present in excess in the reaction mixture. Addition of diphenyl phosphate (3 equiv.; ^31^P NMR, δ: −12.9 ppm) led to the immediate disappearance of both (dimannosyloxy)­phosphorane **17** and betaine **15**, accompanied by the appearance of new peaks at δ: −13.9 and −14.2 ppm, corresponding to the β- and α-glycosyl phosphates **2** and **3**, respectively, as well as a peak at δ: + 51 ppm (Figure 3A). The latter was assigned to the previously described phosphonium salt **15-H** (protonated betaine conjugated with a phosphoric acid anion).

**Figure 3:**
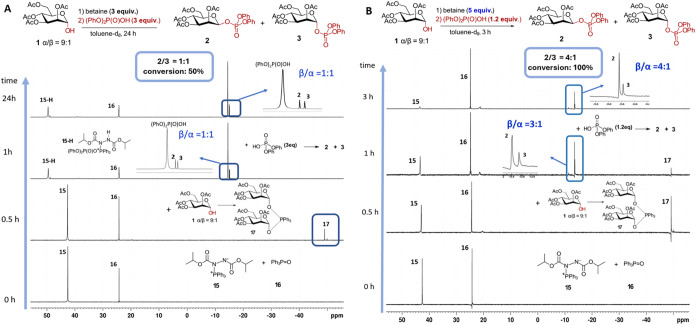
Stereoselectivity of the phosphorylation reaction of Man-lactol **1**. (A) The stereoselectivity of phosphorylation is compromised by an excess of the acidic component (diphenyl phosphate) (reaction protocol *MIP-nonselective*); (B) stereoselective phosphorylation of lactol **1** with the inversion of configuration via the formation of phosphorane intermediate **17** (reaction protocol *MIP-inversion*).

According to NMR analysis, β- and α-glycosyl phosphates **2** and **3** were formed in a 1:1 ratio, while only 50% of starting lactol **1** was consumed (reaction protocol *MIP-nonselective*). Extending the reaction time to 24 h did not improve the reaction outcome, indicating that without the detectable presence of phosphorane **17** and betaine **15** (which can recycle lactol **1** to **17**) no further phosphorylation occurs (Figure 3A). Surprisingly, and contrary to the previously proposed mechanism of the classic Mitsunobu transformation^33b^ and Mitsunobu phosphorylation of primary hydroxyl groups,^24a^ the phosphonium salt **15-H** did not react with the anomeric lactol **1** to form the reactive species **17**, as neither the α/β ratio nor the degree of conversion of **1** to phosphates **2** or **3** changed within 24 h. The rapid reaction of the betaine with an excess of phosphoric acid diester leads to the formation of unreactive phosphonium salt **15-H**, which prevents the phosphorylation reaction from reaching completion and results in low stereoselectivity. At this point, we concluded that reducing the amount of phosphoric acid may help to minimize the formation of **15-H**, thereby enhancing the efficiency of glycosyl phosphate formation. Indeed, lowering the amount of diphenyl phosphate to 1 equiv improved stereoselectivity (**2/3** = β/α = 3:1); however, the overall yield remained suboptimal (Figure S8).

Considering the relatively low stability of phosphorane **17** in the presence of protected phosphoric acid, we hypothesized that the continuous presence of betaine in the reaction mixture is crucial for the ongoing recycling of lactol **1** to regenerate the reactive species **17**. Since the second reaction step—the formation of the glycosyl phosphate from **17**—is rate-limiting, the consumption of phosphoric acid in this step is likely slower than its involvement in the protonation of betaine to form phosphonium salt **15-H**. Therefore, by increasing the amount of betaine while simultaneously limiting the quantity of the protected phosphoric acid, the formation of **15-H** could potentially be avoided, and a sufficient amount of betaine would remain available to recycle lactol **1** into phosphorane **17**, thereby enhancing the efficiency of glycosyl phosphate formation with inversion of the configuration.

Along these lines, further optimization of the reaction conditions with respect to stereoselectivity and yield revealed that using a larger excess of betaine (5 equiv) ensures continuous recycling of lactol **1** toward **17** and maintains a higher concentration of (di-α-mannosyloxy)­phosphorane **17** in the reaction mixture. The latter readily reacts with a limited amount of diphenyl phosphate (1.2 equiv) to form anomeric phosphates **2** and **3** (**2/3** = β/α = 3:1) after 1 h of reaction as shown by the ^31^P NMR analysis, while both **15** and betaine **17** remain present in sufficient quantities (Figure 3B). Notably, the signal at δ: + 51 ppm corresponding to the phosphonium salt **15-H** was not observed at any point during the reaction, indicating that an equimolar amount of phosphoric acid derivative is sufficient to react with **17** to form glycosyl phosphates but not to protonate betaine. After 3 h of reaction under these conditions (reaction protocol *MIP-inversion*), complete conversion of lactol **1** was achieved, and the stereoselectivity of mannosyl phosphate formation improved to β/α = 4:1 (Figure 3B).

To better understand the reaction kinetics and assess whether less bulky or more flexible protecting groups on the phosphorus atom of the phosphoric acid diester could promote a stronger shift toward an SN2 mechanism, diallyl- and dibenzyl phosphates were employed as the acidic components in the phosphorylation reaction, conducted in the NMR tube using toluene-d*_8_* as the solvent. Predominant formation of the β-configured mannosyl phosphates **4** and **6** through a reaction mechanism involving the generation of phosphorane **17**, which was continuously recycled from lactol **1** and betaine **15** under the application of a limited amount (1–1.2 equiv) of diallyl and dibenzyl phosphates, was monitored step by step using ^31^P NMR spectroscopy (Figures S9 and S10). Increasing the proportion of phosphoric acid deliberately led to a loss of stereoselectivity and inhibition of the phosphorylation reaction (reaction protocol *MIP-nonselective*), accompanied by the appearance of a signal at δ = +51 ppm corresponding to **15-H** (Figure S11). By contrast, reconditioning the system in favor of phosphorane **17** (by adding additional preconditioned betaine) accelerated phosphorylation and enhanced stereoselectivity of phosphorylation with inversion of configuration (Figure S9).

When Hep-lactol **8** was applied as a nucleophilic component, the best stereoselectivity and conversion, according to ^31^P NMR, were achieved using diphenyl phosphate (Figure 4A) and dibenzyl phosphate (Figure 4B) as acids. After preconditioning betaine (5 equiv) in the NMR tube, and replenishing the system with Hep-lactol **8**, the formation of phosphorane **18** was monitored by ^31^P NMR spectroscopy. Supplementing the reaction mixture with a limited amount of the corresponding protected phosphoric acid led to the formation of the expected β-heptosyl phosphates **9** and **11** as the major products within 2 h of reaction (β/α = 4:1) (Figure 4). These experiments demonstrate the robustness and reproducibility of our new reaction protocol (reaction protocol *MIP-inversion*), exhibiting excellent stereoselectivity and underscoring the pivotal role of phosphorane **18** as the key intermediate in the anomeric phosphorylation of d-*glycero*-α-d-*manno*-heptose with the inversion of configuration.

**Figure 4:**
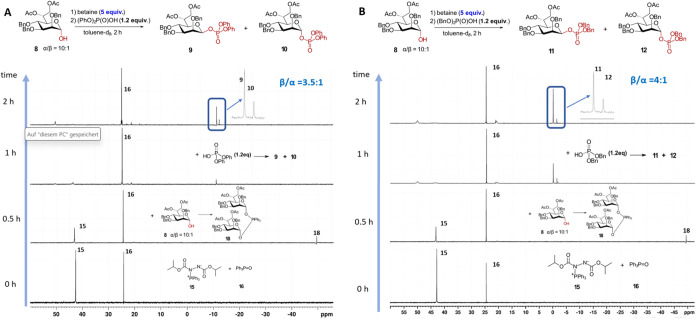
Stereoselectivity of the phosphorylation reaction of Hep-lactol **8**. (A) Phosphorylation with the inversion of configuration via formation of phosphorane **18** by using a limited amount of diphenyl phosphate (reaction protocol *MIP-inversion*); (B) following up the steps of stereoselective synthesis of β-heptosyl phosphate **11** by ^31^P NMR spectroscopy (reaction protocol *MIP-inversion*).

Based on the mechanistic and kinetic studies described above, we performed the anomeric phosphorylation on a practical scale using Man-lactol **1** and Hep-lactol **8** as nucleophilic components, along with three different phosphoric acid derivatives, and successfully isolated the corresponding mannosyl and heptosyl phosphates in pure anomeric form (Scheme 1 and Table 1). Following the proposed reaction protocol, a 5-fold excess of betaine was preconditioned in a flask, followed by the addition of either Man-lactol **1** or Hep-lactol **8** to generate phosphorane **17** or **18**, respectively (Scheme 2, path I). After 10–15 min, the reaction mixture was replenished with a limited amount (1.2 equiv) of the corresponding protected phosphoric acid and allowed to react for 3 h. The use of diphenyl and dibenzyl phosphates for coupling with Man-lactol **1** provided improved stereoselectivity, compared to diallyl phosphate, and afforded β-mannosyl phosphates **2** and **4** in 68% and 65% yields, respectively (Table 1).

**Scheme 2:**
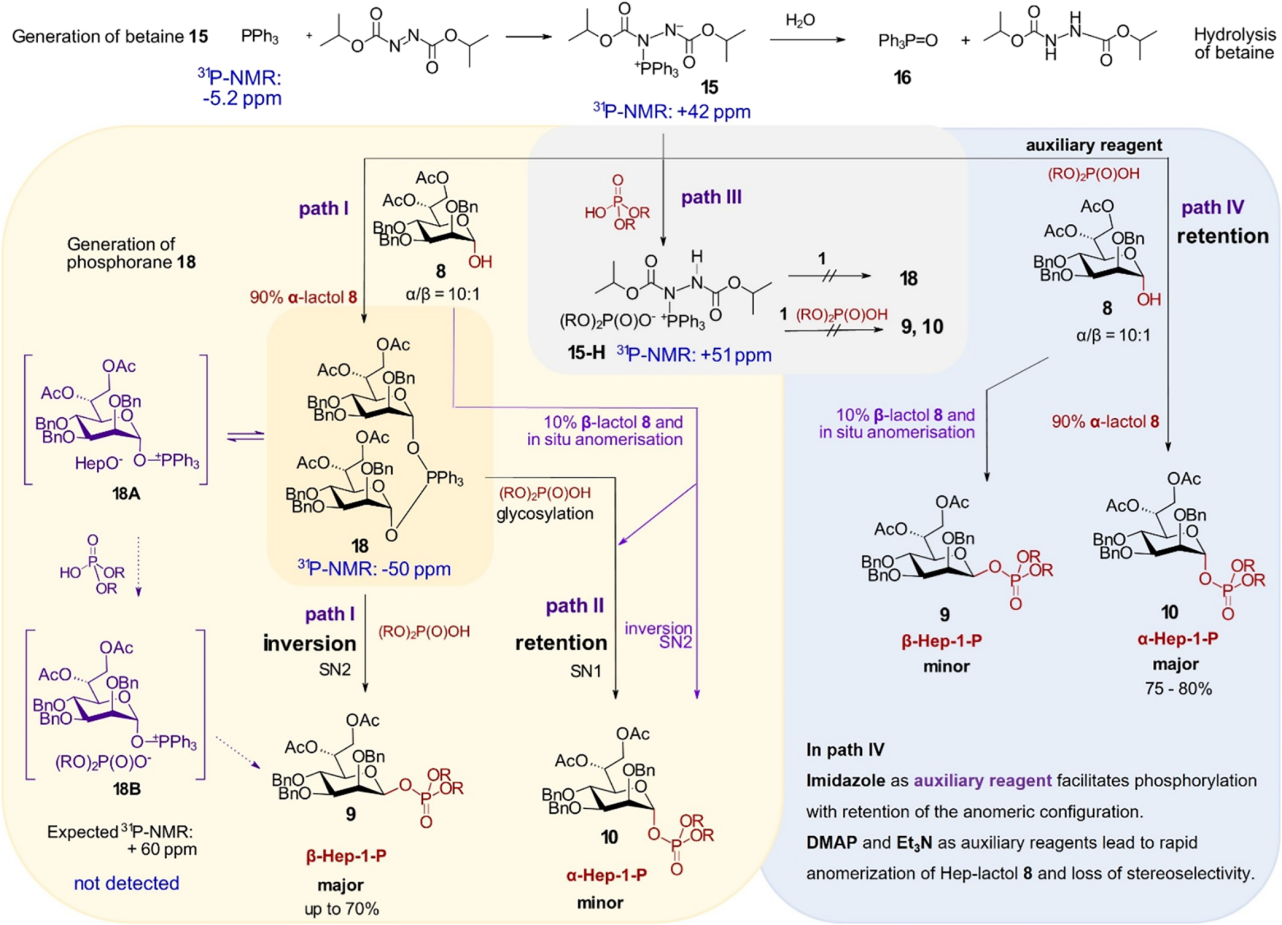
Anomeric Phosphorylation of Hep-lactol **8** under Modified Mitsunobu Reaction Conditions: Reaction Pathways^a^

a Reagents and conditions. Path I and path II (*protocol MIP-inversion*): (1) toluene, betaine (5 equiv), (2) lactol **8** (1 equiv), (3) (RO)_2_P­(O)­OH (1.2 equiv), 2 h, −10 °C. Path III (*protocol MIP-no reaction*): (1) toluene, betaine (3 equiv), (2) lactol **8** (1 equiv), (3) (RO)_2_P­(O)­OH (6 equiv), 3 h, −10 °C to r.t.; path IV (*protocol MIP-retention*): (1) betaine (5 equiv), lactol **8** (1 equiv), imidazole (10 equiv), (RO)_2_P­(O)­OH (1.5 equiv), 1 h, r.t.

Phosphorylation reactions using Hep-lactol **8** were performed at −10 °C to support the integrity of (diheptosyloxy)­phosphorane **18**, giving an excellent 71% isolated yield of the corresponding β-heptosyl phosphates **9** and **11** (Table 1). The stereochemical outcome of this straightforward and reliable phosphorylation procedure, performed under Mitsunobu reaction conditions using inexpensive and readily available reagents, was consistently reproducible.

### Synthesis of Glycosyl Phosphates with Retention of Anomeric Configuration

Since all previously known reports on carbohydrate phosphorylation under Mitsunobu reaction conditions involve the use of an auxiliary reagent, commonly Et_3_N, to enhance the reaction rate and overall conversion, we studied the influence of additives on the reaction mechanism using ^31^P NMR spectroscopy. According to the literature^26–28^ and our initial experiments (Table S1), this transformation has moderate stereoselectivity, yielding products formed with both inversion and retention of configuration. To investigate the consequences of the use of auxiliary reagents, we studied the anomeric phosphorylation of lactols **1** and **8** employing diphenyl phosphate as the acidic component. Initial experiments revealed that the classical Mitsunobu reaction mechanism is disrupted when an auxiliary reagent, such as Et_3_N or DMAP, is introduced. When following the protocol established for the formation of β-mannosyl phosphates, with the sole modification of including an additive (Et_3_N or DMAP), the reaction lacked stereoselectivity and produced a mixture of anomeric phosphates (Figure S12). To promote phosphorylation with the retention of anomeric configuration, the formation of phosphorane **17** was bypassed by changing the reagent addition sequence. Specifically, betaine was no longer premixed with lactol **1** to form phosphoranes **17**, but all reactants (lactol, protected phosphoric acid, and auxiliary reagent) were added simultaneously to the preformed betaine. This reaction protocol, when combined with DMAP as an additive, led to a complete loss of stereoselectivity and the formation of β/α mixtures of mannosyl phosphates ranging from 2:1 to 1:1 (Table 2 and Figure S12). Considering the well-documented ability of DMAP to promote rapid anomerization of α-Man-lactol to its β-counterpart,^10a,19,20^ which is reflected in the final α/β ratio of the resulting glycosyl phosphates, we explored the use of imidazole as an alternative auxiliary reagent. This adjustment restored stereoselectivity and led to the predominant formation of α-configured mannosyl phosphate **3** (**2**/**3** = β/α = 1:5) and α-heptosyl phosphate **10** (**9/10** = β/α = 1:6), presumably via the formation of a phosphorimidazolide intermediate. Conducted on a practical scale, this reaction protocol (*MIP-retention*) ensured the formation of α-mannosyl diphenyl phosphate **3** and α-heptosyl diphenyl phosphate **10** in a 75% isolated yield, along with the corresponding β-configured phosphates in a 10–15% yield (Table 2). Expansion of the nature of protecting groups at phosphorus to dibenzyl and diallyl phosphates afforded the corresponding α-mannosyl phosphates **5** and **7**, as well as α-heptosyl phosphates **12** and **14**, in a 77% yield (Table 2).

**Table 2:** Synthesis of Glycosyl Phosphates with Retention of Anomeric Configuration in the Presence of an Auxiliary Reagent^a^

			yield/product	
lactol	phosphate	Aux. reagent	β %	α %
**1**	(PhO)_2_P(O)OH	Im	15 (**2**)	75 (**3**)
**1**	(PhO)_2_P(O)OH	DMAP	42 (**2**)	43 (**3**)
**1**	(BnO)_2_P(O)OH	Im	12 (**4**)	69 (**5**)
**1**	(AllylO)_2_P(O)OH	Im	15 (**6**)	70 (**7**)
**8**	(PhO)_2_P(O)OH	Im	13 (**9**)	75 (**10**)
**8**	(PhO)_2_P(O)OH	DMAP	41 (**9**)	42 (**10**)
**8**	(BnO)_2_P(O)OH	Im	14 (**11**)	77 (**12**)
**8**	(AllylO)_2_P(O)OH	Im	12 (**13**)	77 (**14**)

a Reaction conditions are indicated in Figure S12 and in the Experimental Section: reaction protocol *MIP-retention*.

### Mechanistic Considerations and Reaction Pathways

Taken together, our findings demonstrate that the anomeric phosphorylation of pyranose-derived lactols under modified Mitsunobu conditions can proceed through a classic Mitsunobu pathway involving the formation of (dipyranosyloxy)­phosphoranes, such as **17** or **18**, from preformed betaine **15** and the corresponding pyranose lactol **1** or **8** (path I, Schemes 2 and S1). These phosphoranes are key reaction intermediates that enable the reaction to proceed via the SN2 pathway, generating anomeric phosphates with inversion of configuration (Scheme 2, path I), while the quantity of protected phosphoric acid must be limited to prevent protonation of betaine and formation of unreactive adduct **15-H** (Scheme 2, path III). To guarantee high stereoselectivity and yields, the reaction conditions must be adjusted to ensure the continuous regeneration of (dipyranosyloxy)­triphenylphosphorane **18** from betaine **15** and α-configured lactol **8**, which consequently reacts with phosphoric acid derivative to produce the glycosyl phosphate with inversion of the anomeric configuration.

The formation of an oxyphosphonium salt such as **18B** (with an expected ^31^P NMR signal at δ: ∼ +59 to +65 ppm at −50 °C)^33b,34^ has never been observed under our experimental conditions (toluene-d_8_, r.t.), regardless of the order of reagent addition. Such a phosphonium salt has been proposed to form either via rapid reaction of an alkoxyphosphorane with the acid or through a much slower reaction of protonated betaine with the alcohol and to serve as a key reactive intermediate (detectable at −50 °C) that ultimately yields the expected products with inversion of configuration.^31b,33b,37^ An equilibrium may exist between phosphonium salt **18B** and phosphorane **18** at **–**50 °C, with **18** being the predominant species at rt, as in our case. **18B** could also act as an intermediate in the decomposition of **18** in the presence of phosphoric acid, ultimately leading to regeneration of lactol **8**.

The anomerically activated phosphorane **18** (as well as its β-configured counterpart, formed from the 10% proportion of the β-lactol) can also act as a glycosyl donor, undergoing a phosphoric acid-promoted glycosylation reaction that proceeds via the formation of an intermediate oxocarbenium ion. The latter can then react with a phosphoric acid diester anion through an SN1 mechanism, potentially affording a mixture of anomeric glycosyl phosphates (Scheme 2, path II). The observed 1,2-*trans* stereoselectivity leading to the formation of α-phosphate **10** (as a result of the glycosylation reaction) can be rationalized by electronic and steric effects, together with the specific protecting group pattern, which promotes predominant α-stereoselective glycosylation in the d-*manno*- and d-*manno*-heptose series.^38^ Complementary to this pathway, slow anomerization of the α-lactol to its β-counterpart over the 3 h reaction period might also contribute to the partial loss of stereoselectivity.

Anomeric phosphorylation under modified Mitsunobu conditions can also proceed with the retention of configuration when auxiliary reagents, such as Et_3_N, DMAP, or imidazole, are employed. In this pathway, direct phosphorylation of α-lactol **8** is facilitated by the auxiliary reagent, thereby bypassing the formation of phosphorane **18**. Imidazole, used in large excess, appears to serve a dual role: accelerating the deprotonation of the anomeric hydroxyl in **8** to form an oxyanion and likely interacting with the phosphoric acid diester to form a phosphorimidazolide, which subsequently reacts with the anomeric oxyanion to generate α-heptosyl phosphate with the retention of anomeric configuration (Scheme 2, path IV). Notably, the use of Et_3_N or DMAP as auxiliary reagents compromises stereoselectivity by promoting the anomerization of lactol **8** to its β-counterpart.

## Conclusions

Our study reveals that anomeric phosphorylation of pyranose-derived lactols under modified Mitsunobu conditions can proceed either through the traditional Mitsunobu SN2 pathway resulting in efficient formation of glycosyl phosphates with the inversion of anomeric configuration or through an alternative mechanism in which the glycosyl phosphates retain the configuration of the starting lactol. The (dipyranosyloxy)­phosphorane intermediates play a crucial role in promoting SN2-type displacement using phosphoric acid derivatives, leading to the formation of glycosyl phosphates with the inversion of stereochemistry. We have developed a highly efficient reaction protocol that enables the stereoselective synthesis of β-heptosyl phosphates, biochemically important molecules, and precursors of the innate immune modulator ADP-heptose. A switch from inversion to retention of the anomeric configuration can be achieved by supplementing Mitsunobu-type phosphorylation with auxiliary reagents, such as imidazole. Although our studies focused on a “borderline case” of anomeric phosphorylation, using α-d-*manno*-configured lactols to generate mannosyl and heptosyl phosphates of either β- or α-configuration, the reaction mechanisms established here can be extended to a variety of other pyranoses to synthesize glycosyl phosphates with high stereoselectivity by appropriately combining and tuning the two pathways.

## Experimental Section

### General Information

Reagents and solvents were purchased from commercial suppliers and used without further purification unless otherwise stated. Solvents were dried by storage over activated MS for at least 24 h prior to use (dichloromethane and toluene 4 Å; acetonitrile 3 Å). Residual moisture was determined by coulometric titration on a Mitsubishi CA21 Karl Fischer apparatus and did not exceed 20 ppm. Reactions were monitored by TLC performed on silica gel 60 F254 HPTLC precoated glass plates with a 25 mm concentration zone (Merck). Spots were visualized by dipping into a sulfuric acid–*p*-anisaldehyde solution and subsequent charring at 250 °C. Solvents were removed under reduced pressure at ≤40 °C. NMR spectra were recorded on a Bruker Avance III 600 spectrometer (^1^H at 600.22 MHz; ^13^C at 150.93 MHz; ^31^P at 242.97 MHz) by using standard Bruker NMR software. Chemical shifts are reported in ppm, where ^1^H NMR spectra recorded from samples in CDCl_3_ were referenced to internal TMS and ^13^C spectra were referenced to the corresponding solvent signal (77.16 ppm for CDCl_3_). NMR spectra recorded from samples in other solvents were referenced to residual solvent signals (for CD_3_OD 3.31 and 49.00 ppm; for CD_2_Cl_2_ 5.32 and 53.84 ppm; for toluene-d_8_ 2.08 and 128.6/21.2 ppm; and for ^1^H and ^13^C NMR, respectively). ^31^P NMR spectra are externally referenced to PPh_3_ (−5.00 ppm).^39^ Structural assignments were made with additional information from the gCOSY, gHSQC, and gHMBC experiments. High-resolution mass spectrometry (HRMS) was carried out on acetonitrile or DCM solutions via LC-TOF MS (Agilent 1200SL HPLC and Agilent 6210 ESI-TOF, Agilent Technologies). Data sets were analyzed using Agilent Mass Hunter Software. Optical rotation was measured on a PerkinElmer 243B polarimeter equipped with a Haake water circulation bath and a Haake D1 immersion circulator for temperature control or an Anton Paar MCP 100 polarimeter featuring an integrated Peltier temperature control. All [α]_20_^D^ values are reported in units of deg·dm^–1^·cm^3^·g^–1^, and the corresponding concentrations are reported in g/100 mL.

### Diphenyl (2,3,4-Tri-*O*-benzyl-6,7-di-*O*-acetyl-d-*glycero*-β-d-*manno-*heptopyranosyl)­phosphate (**9**)

#### Protocol MIP-Inversion

To a cooled (−10 °C) preconditioned solution of betaine [prepared from triphenylphosphine (0.443 mmol, 89.5 mg) and diisopropyl azodicarboxylate (DIAD) (0.443 mmol, 9 μL) by stirring for 10 min at rt in toluene (5 mL) under an atmosphere of Ar (the formation of betaine was controlled by ^31^P NMR)], a solution of **8** (0.089 mmol, 50 mg) in dry toluene (1 mL) was added. The stirring was continued for 20 min, and diphenyl phosphate (1.2 equiv., 0.106 mmol, 26 mg) was added at −10 °C under an atmosphere of Ar. The reaction mixture was stirred for 1 h at −10 °C and for 2 h at 10 °C. The mixture was concentrated, and the residue was purified by column chromatography on silica gel (toluene/EtOAc, 9:1 → 7:3) to afford **9** as a colorless syrup (50 mg, 0.063 mmol, 71%); *R*_f_ = 0.3 (toluene/EtOAc, 7:3) and **10** as a colorless syrup (18 mg, 0.022 mmol, 26%). (**9)**: *R*_f_ = 0.5 (toluene/EtOAc, 7:3); [α]_20_^D^ = +8 (*c* = 0.4, CHCl_3_); ^1^H NMR (600 MHz, CDCl_3_), δ: 7.31 (m, 21H, Ph), 7.20 (m, 4H, Ph), 5.53 (m, 1H, H-6), 5.43 (dd, 1H, ^3^ *J*_1‑2_ = 0.8 Hz, ^3^ *J*_1‑P_ = 7.2 Hz, H-1), 4.90 (AB, 1H, ^2^ *J* = 10.8 Hz, C*H*_2_-Ph), 4.73 (AB, 1H, ^2^ *J* = 10.8 Hz, C*H*_2_-Ph), 4.66 (AB, 1H, ^2^ *J* = 11.9 Hz, C*H*_2_-Ph), 4.61 (AB, 1H, ^2^ *J* = 11.9 Hz, C*H*_2_-Ph), 4.53 (AB, 1H, ^2^ *J* = 11.5 Hz, C*H*_2_-Ph), 4.50 (AB, 1H, ^2^ *J* = 11.5 Hz, C*H*_2_-Ph), 4.37 (dd, 1H, ^3^ *J*_6,7*a*_ = 3.4 Hz, ^2^ *J*_*7a,7b*_ = 12.2 Hz, H7), 4.10 (dd, 1H, ^3^ *J*_6,7*a*_ = 7.7 Hz, ^2^ *J*_7*a*,7*b*_ = 12.2 Hz, H7), 3.93 (t, 1H, ^3^ *J*_2‑3_ = ^3^ *J*_3‑4_ = 9.0 Hz, H-4), 3.85 (d, 1H, ^3^ *J*_2‑3_ = 2.5 Hz, H-2), 3.64 (dd, 1H, ^3^ *J*_4–5_ = 9.2 Hz, ^3^ *J*_5–6_ = 2.7, H5), 3.55 (dd, 1H, ^3^ *J*_2‑3_ = 2.8, ^3^ *J*_3–4_ = 8.8 Hz, H-3), 2.01 (s, 3H, C*H*_3,_ Ac), 1.97 (s, 3H, C*H*_3,_ Ac). ^13^C­{1H} NMR (150.9 MHz, CDCl_3_), δ: 170.7 (*C*O–Ac); 170.1 (*C*O–Ac); 138.3, 137.9, and 137.8 (ipso C- Ph); 130.0, 129,9, 128.6, 128.59, 128.4, 128.0, 127.9, 127.9, 127.8, 127.7, 125.7, 125.7, 120.6, 120.5, 120.3, and 120.6 (C-Ph); 97.9 (C-1, ^2^ *J*_1,P_ = 5.5 Hz), 81.1 (C-3), 76.2 (C-5), 74.9 (*C*H_2_–Ph); 74.5 (C-2, ^2^ *J*_2,P_ = 7.7 Hz), 74.4 (*C*H_2_–Ph); 73.8 (C-4); 72.2 (*C*H_2_–Ph); 70.5 (C-6), 62.4 (C-7), 21.1 and 20.9 (2 × *C*H_3,_ Ac); ^31^P NMR (243.0 MHz, CDCl_3_), δ: −13.7; HRMS (ESI) *m*/*z* calcd. for [M + NH_4_]^+^ C_44_H_49_NO_12_P: *m*/*z* = 814.298, found: *m*/*z* = 814,298.

### Diphenyl (2,3,4-Tri-*O*-benzyl-6,7-di-*O*-acetyl-d-*glycero*-α-d-*manno-*heptopyranosyl)­phosphate (**10**)

#### Protocol MIP-Retention

To a cooled (−10 °C) preconditioned solution of betaine [prepared from triphenylphosphine (0.177 mmol, 46.5 mg) and diisopropyl azodicarboxylate (0.036 mmol, 3 μL) by stirring for 10 min at rt under an atmosphere of Ar] in toluene/THF (1:1, 3 mL) was added a solution of lactol **8** (0.035 mmol, 20 mg) in toluene (1 mL) under an atmosphere of Ar. The stirring was continued for 15 min at −10 °C, and diphenyl phosphate (0.071 mmol, 20 mg) and imidazole (0.354 mmol, 24 mg) were added under an atmosphere of Ar at −10 °C. The stirring was continued for 2 h at 0 °C; the mixture was diluted with toluene (5 mL) and concentrated. The residue was purified by column chromatography on silica gel (toluene/EtOAc, 9:1 → 7:3) to afford **9** as a colorless syrup (4 mg, 0.005 mmol, 13%); *R*_f_ = 0.3 (toluene/EtOAc, 7:3) and **10** as a colorless syrup (21 mg, 0.026 mmol, 75%). (**10**): *R*_f_ = 0.5 (toluene/EtOAc, 7:3); [α]_20_^D^ = +11 (*c* = 0.2, CHCl_3_); ^1^H NMR (600 MHz, CDCl_3_) δ 7.13–7.23 (m, 25H, Ph), 5.90 (dd, 1H, ^3^ *J*_1‑2_ = 2.1 Hz, ^3^ *J*_1‑*P*_ = 6.1 Hz, H-1), 5.48 (m, 1H, H-6), 4.94 (AB, 1H, ^2^ *J* = 10.7 Hz, C*H*_2_ OBn), 4.75 (AB, 1H, ^2^ *J* = 10.6 Hz, C*H*_2_ OBn), 4.67 (AB, 1H, ^2^ *J* = 12.2 Hz, C*H*_2_ OBn), 4.63 (AB, 1H, ^2^ *J* = 12.1 Hz, C*H*_2_ OBn), 4.48 (AB, 1H, ^2^ *J* = 11.6 Hz, C*H*_2_ OBn), 4.44 (AB, 1H, ^2^ *J* = 11.7 Hz, C*H*_2_ OBn), 4.36 (dd, 1H, ^3^ *J*_6,7*a*_ = 3.4 Hz, ^2^ *J*_7*a*,7*b*_ = 12.2 Hz, H7), 4.15 (dd, 1H, ^3^ *J*_6,7*a*_ = 8.4 Hz, ^2^ *J*_7*a*,7*b*_ = 12.2 Hz, H7), 4.00 (m, 2H, H-4, H-5), 3.83 (dd, 1H, ^3^ *J*_2*‑*3_ = 2.9 Hz, ^3^ *J*_*3–4*_ = 8.6 Hz, H-3), 3.72 (t, 1H, ^3^ *J*_1‑2_ = ^3^ *J*_2*‑*3_ = 2.5 Hz, H-2), 1.97 (s, 3H, C*H*_3,_ Ac), 1.83 (s, 3H, C*H*_3,_ Ac); ^13^C­{1H} NMR (150.9 MHz, CDCl_3_): 170.7 (*C*O–Ac), 170.2 (*C*O–Ac), 138.0, 137.9, and 137.6 (C_q_-Ph), 130.0, 128,6, 128.6, 128.6, 128.4, 128.1, 128.0, 127.9, 127.8, 125.7, 120.3, 120.3, 120.3, and 120.3 (C-Ph), 97.9 (C-1, ^2^ *J*_1,P_ = 5.8 Hz), 79.1 (C-3), 75.2 (*C*H_2_–OBn), 74.9 (C5); 74.2 (C-2, ^2^ *J*_2,P_ = 9.1 Hz), 73.9 (C4); 73.0 (*C*H_2_–OBn); 72.4 (*C*H_2_–OBn); 70.59 (C-6), 62.7 (C-7), 20.9 and 20.9 (2 × *C*H_3_, Ac); ^31^P NMR (243.0 MHz, CDCl_3_): -13.9; HRMS (ESI) *m*/*z* calcd. for [M + NH_4_]^+^ C_44_H_49_NO_12_P: *m*/*z* = 814.2999, found: *m*/*z* = 814,2975.

### Dibenzyl (2,3,4-Tri-*O*-benzyl-6,7-di-*O*-acetyl-d-*glycero*-β-d-*manno*-heptopyranosyl)­phosphate (**11**)

#### Protocol MIP-Inversion

To a cooled (−10 °C) preconditioned solution of betaine [prepared from triphenylphosphine (0.443 mmol, 89.5 mg) and diisopropyl azodicarboxylate (0.443 mmol, 9 μL) by stirring at r.t for 10 min under an atmosphere of Ar (controlled by ^31^P NMR for the formation of betaine)] in toluene (5 mL), a solution of **8** (0.089 mmol, 50 mg) was added under an atmosphere of Ar. The reaction mixture was stirred for 15 min, and dibenzyl phosphate (1.17 equiv, 0.104 mmol, 29 mg) was added at −10 °C. The stirring was continued for 1 h at −10 °C and for 2 h at 10 °C under an atmosphere of Ar. The reaction mixture was diluted with toluene (5 mL) and concentrated. The residue was purified by column chromatography on silica gel (toluene/EtOAc, 8:2 → 7:3) to give **11** as a colorless syrup (51 mg, 0.062 mmol, 70%) and **12** as a colorless syrup (15 mg, 0.018 mmol, 21%). (**11**): *R*_f_ = 0,2 toluene/EtOAc 7:3 [α]_20_^D^ = −7 (c = 0.3, CHCl_3_); ^1^H NMR (600 MHz, CDCl_3_) δ 7.26–7.37 (m, 25H), 5.54 (m, 1H, H-6), 5.22 (dd, 1H, ^3^ *J*_1–2_ = 0.7 Hz, ^3^ *J*_*1‑P*_ = 7.3 Hz, H-1), 5.12 (d, 2H, ^2^ *J* = 7.7 Hz, C*H*_2_ OBn), 5.03 (dd, 2H, ^3^ *J*_*CH2‑Ph‑P*_ = 2.7, ^2^ *J* = 8.2 Hz, 2H, C*H*_2_ OBn), 4.93 (AB, 1H, ^2^ *J* = 10.8 Hz, C*H*_2_ OBn), 4.75 (AB, 1H, ^2^ *J* = 12.0 Hz, C*H*_2_ OBn), 4.73 (AB, 1H, ^2^ *J* = 10.8 Hz, C*H*_2_ OBn), 4.70 (AB, 1H, ^2^ *J* = 12.0 Hz, C*H*_2_ OBn), 4.54 (AB, 1H, ^2^ *J* = 11.8 Hz, C*H*_2_ OBn), 4.50 (AB, 1H, ^2^ *J* = 11.8 Hz, C*H*_2_ OBn), 4.38 (dd, 1H, ^3^ *J*_6,7a_ = 3.4 Hz, ^2^ *J*_7a,7b_ = 12.2 Hz, H-7), 4.16 (dd, 1H, ^3^ *J*_6,7a_ = 8.0 Hz, ^2^ *J*_7a,7b_ = 12.2 Hz, H-7), 3.94 (t, 1H, ^3^ *J*_2‑3_ = ^3^ *J*_3‑4_ = 9.2 Hz, H-4), 3.80 (d, 1H, ^3^ *J*_2‑3_ = 2.7 Hz, H-2), 3.61 (dd, 1H, ^3^ *J*_4–5_ = 9.5 Hz, ^3^ *J*_5–6_ = 2.4, H-5), 3.52 (dd, 1H, ^3^ *J*_2‑3_ = 2.8, ^3^ *J*_*3–4*_ = 8.9 Hz, H-3), 1.97 (s, 3H, *CH*_3,_ Ac), 1.94 (s, 3H, *CH*_3,_ Ac); ^13^C­{1H} NMR (150.9 MHz, CDCl_3_): 170.7 (*C*O–Ac); 170.1 (*C*O–Ac), 138.5, 137.9 and 137.83­(C_q_- Ph), 128.7, 128,7, 128.6, 128.6, 128.6, 128.1, 128.0, 128.0, 127.9, 127.9, 127.8, and 127.7 (*C*-Ph), 97.4 (C-1, ^2^ *J*_1,P_ = 5.3 Hz), 81.6 (C-3), 76.2 (C-5), 75.0 (*C*H_2_–Ph), 74.4 (C-2, ^2^ *J*_2,P_ = 7.8 Hz), 74.4 (*C*H_2_–OBn), 73.7 (C-4), 72.1 (*C*H_2_–OBn), 69.8 (*C*H_2_–Ph, ^3^ *J*_CH2‑Ph,P_ = 5.4 Hz), 69.7 (*C*H_2_–Ph, ^3^ *J*_CH2‑Ph,P_ = 5.5 Hz), 70.6 (C-6), 62.5 (C-7), 21.0 and 20.9 (2 × *C*H_3_, Ac); ^31^P NMR (243.0 MHz, CDCl_3_): - 2.5. HRMS (ESI) *m*/*z* calcd. for [M + NH_4_]^+^ C_46_H_53_NO_12_P: *m*/*z* = 842.3305, found: *m*/*z* = 842.3290.

### Dibenzyl (2,3,4-Tri-*O*-benzyl-6,7-di-*O*-acetyl-d-*glycero*-α-d-*manno*-heptopyranosyl)­phosphate (**12**)

#### Protocol MIP-Retention

To a cooled (−10 °C) preconditioned solution of betaine [prepared from triphenylphosphine (0.177 mmol, 47 mg) and diisopropyl azodicarboxylate (DIAD) (0.177 mmol, 3 μL) by stirring for 10 min at room temperature under an atmosphere of Ar] in dry toluene/THF (1:1, 3 mL) was added a solution of lactol **8** (0.035 mmol, 20 mg) in dry toluene (1 mL) under an atmosphere of Ar. The stirring was continued for 5 min, and dibenzyl phosphate (0.04 mmol, 11 mg) and imidazole (0.354 mmol, 24 mg) were added under stirring. The stirring was continued for 2 h at 10 °C; the mixture was concentrated, and the residue was purified by column chromatography on silica gel (toluene/EtOAc, 9:1 → 7:3) to give **11** as a colorless syrup (4 mg, 0.005 mmol, 14%) and **12** as a colorless syrup (22 mg, 0.027 mmol, 77%). (**12**): (*R*_f_ = 0,4 toluene/EtOAc 7:3). [α]_20_^D^ = +10 (*c* = 0.2, CHCl_3_); ^1^H NMR (600 MHz, CDCl_3_) δ 7.27–7.37 (m, 25H), 5.65 (dd, 1H, ^3^ *J*_1–2_ = 1.8 Hz, ^3^ *J*_1*‑P*_ = 6.4 Hz, H-1), 5.50 (m, 1H, H-6), 5.01 (m, 4H, 2 × C*H*_2_ OBn), 4.94 (AB, 1H, ^2^ *J* = 10.7 Hz, C*H*_2_ OBn), 4.74 (AB, 1H, ^2^ *J* = 10.7 Hz, C*H*_2_ OBn), 4.63 (AB, 1H, ^2^ *J* = 12.1 Hz, C*H*_2_ OBn), 4.58 (AB, 1H, ^2^ *J* = 12.1 Hz, C*H*_2_ OBn), 4.51 (AB, 1H, ^2^ *J* = 11.7 Hz, C*H*_2_ OBn), 4.48 (AB, 1H, ^2^ *J* = 11.7 Hz, C*H*_2_ OBn), 4.34 (dd, 1H, ^3^ *J*_6,7*a*_ = 3.4 Hz, ^2^ *J*_7*a*,7*b*_ = 12.2 Hz, H-7), 4.14 (dd, 1H, ^3^ *J*_6,7*a*_ = 8.5 Hz, ^2^ *J*_*7*a,7*b*_ = 12.2 Hz, H-7), 3.97 (m, 2H, H-4, H-5), 3.79 (dd, 1H, ^3^ *J*_*2–3*_ = 3.0, ^3^ *J*_3–4_ = 8.8 Hz, H-3), 3.66 (t, 1H, ^3^ *J*_2–3_ = ^3^ *J*_3–4_ = 2.3 Hz, H-2), 1.97 (s, 3H, C*H*_3,_ Ac), 1.87 (s, 3H, C*H*_3,_ Ac); ^13^C­{1H} NMR (150.9 MHz, CDCl_3_): 170.8 (*C*O–Ac), 170.1 (*C*O–Ac), 138.1, 137.8 (C_q_- Ph), 128.8, 128,8, 128.8, 128.6, 128.5, 128.3, 128.2, 128.1, 128.0, 127.9, 127.8 (C-Ph), 96.0 (C-1, ^2^ *J*_1,P_ = 4.7 Hz), 79.0 (C-3), 75.2 (*C*H_2_–OBn), 74.4 (C-2, ^2^ *J*_2,P_ = 8.2 Hz), 74.4 (C-5), 74.0 (C-4), 72.9 (*C*H_2_–OBn), 72.3 (*C*H_2_–OBn), 70.7 (C-6); 69.7 (*C*H_2_–Ph, ^3^ *J*_CH2‑Ph,P_ = 4.4 Hz), 69.6 (*C*H_2_–Ph, ^3^ *J*_CH2‑Ph,P_ = 5.5 Hz); 62.7 (C-7), 20.9 and 20.9 (2 × *C*H_3_, Ac); ^31^P NMR (243.0 MHz, CDCl_3_): -2.9. HRMS (ESI) *m*/*z* calcd. for [M + NH_4_]^+^ C_46_H_53_NO_12_P: *m*/*z* = 842.3305, found: *m*/*z* = 842.3292.

### Diallyl (2,3,4-Tri-*O*-benzyl-6,7-di-*O*-acetyl-D-*glycero*-β-D-*manno*-heptopyranosyl)­phosphate (**13**)

#### Protocol MIP-Inversion

To a cooled (−10 °C) preconditioned solution of betaine [prepared from triphenylphosphine (0.443 mmol, 89.5 mg) and diisopropyl azodicarboxylate (0.443 mmol, 9 μL) by stirring for 10 min at r.t. under an atmosphere of Ar (controlled by ^31^P NMR for the formation of betaine)] in toluene (5 mL), a solution of **8** (0.089 mmol, 50 mg) in toluene (1 mL) was added and the stirring was continued for 20 min at −10 °C under an atmosphere of Ar. Then, a solution of diallyl phosphate (0.106 mmol, 19 mg) in toluene (0.5 mL) was added under an atmosphere of Ar and the stirring was continued for 1 h at −10 °C and for 2 h at 10 °C. The reaction mixture was diluted with toluene (2 mL) and concentrated. The residue was purified by column chromatography on silica gel (toluene/EtOAc, 8:2 → 6:4) to afford **13** as a colorless syrup (45 mg, 0.056 mmol, 64%) (*R*_f_ = 0,22 toluene/EtOAc 7:3) and **14** as a colorless syrup (15 mg, 0.019 mmol, 21%). (**13**): (*R*_f_ = 0,3 toluene/EtOAc 7:3); [α]_20_^D^ = −40 (*c* = 0.2, CHCl_3_); ^1^H NMR (600 MHz, CDCl_3_) δ 7.39–7.31 (m, 15H, Ph), 5.96 (m, 1H, C*H*-Allyl), 5.89 (m, 1H, C*H*-Allyl), 5.54 (m, 1H, H-6), 5.37 (dd, 1H, ^2^ *J*_*=CH2cis,=CH2trans*_ = 1.5 Hz,^3^J_*CH=,=CH2cis*_ = 17.1 Hz, =C*H*_2cis_), 5.33 (dd, 1H, ^2^ *J*_*=CH2cis, =CH2trans*_ = 1.5 Hz,^3^ *J*_*CH=,=CH2trans*_ = 17.1 Hz, =CH_2trans_), 5.26–5.22 (m, 3H, =C*H*_2cis,_ H-1,=C*H*_2trans_), 4.93 (AB, 1H, ^2^ *J* = 10.8 Hz, C*H*_2_ OBn), 4.86 (AB, 1H, ^2^ *J* = 12.0 Hz, C*H*_2_ OBn), 4.78 (AB, 1H, ^2^ *J* = 12.0 Hz, C*H*_2_ OBn), 4.75 (AB, 1H, ^2^ *J* = 10.8 Hz, C*H*_2_ OBn), 4.60 (m, 2H, OCH*H*, All.), 4.56 (AB, 2H, ^2^ *J*_*AB*_ = 9.4 Hz, C*H*_2_ OBn), 4.52 (m, 2H, OCH*H*, All.), 4.39 (dd, 1H, ^3^ *J*_6,7*a*_ = 3.4 Hz, ^2^ *J*_7*a*,7*b*_ = 12.2 Hz, H-7), 4.20 (dd, 1H, ^3^ *J*_6,7*a*_ = 7.9 Hz, ^2^ *J*_7*a*,7*b*_ = 12.2 Hz, H-7), 3.94 (m, 2H, H-2, H-4), 3.93 (d, 1H, ^3^ *J*_2,3_ = 2.4 Hz, H-2), 3.61 (dd, 1H, ^3^ *J*_5–6_ = 2.5 Hz, ^3^ *J*_4–5_ = 9.5 Hz, H-5), 3.56 (dd, 1H, ^3^ *J*_*2–3*_ = 2.8 Hz, ^3^ *J*_*3–4*_ = 8.9 Hz, H-3), 2.01 (s, 3H, C*H*_3,_ Ac), 1.98 (s, 3H, C*H*_3_, Ac); ^13^C­{1H} NMR (150.9 MHz, CDCl_3_): 170.8 (*C*O–Ac); 170.0 (*C*O–Ac), 138.4, 137.9, and 137.8 (C_q_- Ph), 132.3 (*C*H=CH2), 132.3 (*C*H=CH_2_), 128.6, 128,5, 128.4, 128.0, 127.9, 127.8, 127.7 (C-Ph), 118.5 (CH=*C*H_2_), 118.4 (CH=*C*H_2_), 96.9 (C-1, ^2^ *J*_1,P_ = 5.3 Hz), 81.5 (C-3), 76.2 (C-5), 75.0 (*C*H_2_–OBn), 74.5 (C-2, ^3^ *J*_2,P_ = 7.3 Hz), 74.4 (*C*H_2_–OBn), 73.7 (C-4), 72.2 (*C*H_2_–OBn), 70.6 (C-6), 68.7 (O*C*H_2_, All. ^3^ *J*_OCH2,P_ = 5.6 Hz), 68.5 (2C, O*C*H_2_, All. ^3^ *J*_OCH2,P_ = 4.86 Hz), 62.5 (C-7), 21.1 and 20.9 (2 × *C*H_3_, Ac); ^31^P NMR (243.0 MHz, CDCl_3_): - 2.5; HRMS (ESI) *m*/*z* calcd. for [M + H]^+^ C_38_H_46_O_12_P: *m*/*z* = 725.2726, found: *m*/*z* = 725.2713.

### Diallyl (2,3,4-Tri-*O*-benzyl-6,7-di-*O*-acetyl-d-*glycero*-α-d-*manno*-heptopyranosyl) Phosphate (**14**)

#### Protocol MIP-Retention

To a cooled (−10 °C) preconditioned solution of betaine [prepared from triphenylphosphine (0.168 mmol, 44 mg) and diisopropyl azodicarboxylate (DIAD) (0.168 mmol, 3 μL) in dry toluene/THF (1:1, 3 mL)] was added a solution of lactol **8** (0.034 mmol, 19 mg) in dry toluene (1 mL) under an atmosphere of Ar. The stirring was continued for 5 min, and imidazole (0.337 mmol, 23 mg) and a solution of diallyl phosphate (0.04 mmol, 7 mg) in toluene (0.5 mL) were added under stirring. The reaction mixture was stirred for 2 h at 10 °C, diluted with toluene (5 mL), and concentrated. The residue was purified by column chromatography on silica gel (toluene/EtOAc, 9:1 → 7:3) to afford **13** as a colorless syrup (3 mg, 0.004 mmol, 12%) and **14** as a colorless syrup (19 mg, 0.026 mmol, 77%). (**14**): *R*_f_ = 0,33 toluene/EtOAc 7:3; [α]_20_^D^ = +27 (*c* = 0.1, CHCl_3_); ^1^H NMR (600 MHz, CDCl_3_) δ 7.28–7.36 (m, 15H, Ph), 5.89 (m, 2H, C*H*-Allyl), 5.66 (dd, 1H, ^3^ *J*_1–2_ = 2.2 Hz, ^3^ *J*_1‑*P*_ = 6.4 Hz,, H-1), 5.53 (m, 1H, H-6), 5.34 (t, 2H, ^3^ *J*_*CH=,=CH2cis*_ = 16.5 Hz, 2H, =C*H*_2cis_), 5.24 (t, 2H, ^3^ *J*_*CH=,=CH2trans*_ = 11.0 Hz,=C*H*_2trans_), 4.96 (AB, 1H, ^2^ *J* = 10.8 Hz, C*H*_2_ OBn), 4.77 (AB, 1H, ^2^ *J* = 10.8 Hz, C*H*_2_ OBn), 4.71 (AB, 1H, ^2^ *J* = 12.2 Hz, C*H*_2_-Ph), 4.67 (AB, 1H, ^2^ *J* = 11.9 Hz, C*H*_2_ OBn), 4.61 (AB, 1H, ^2^ *J* = 11.8 Hz, C*H*_2_ OBn), 4.58 (AB, 1H, ^2^ *J* = 11.8 Hz, C*H*_2_ OBn), 4.50 (m, 4H, OCH*H*, All.), 4.40 (dd, 1H, ^3^ *J*_6,7*a*_ = 3.2 Hz, ^2^ *J*_7*a*,7*b*_ = 12.1 Hz, H-7), 4.15 (dd, 1H, ^3^ *J*_6,7*a*_ = 8.7 Hz, ^2^ *J*_7*a*,7*b*_ = 12.1 Hz, H-7), 3.99 (m, 2H, H-4, H-5), 3.88 (dd, 1H, ^3^ *J*_2–3_ = 2.2 Hz, ^3^ *J*_3–4_ = 9.0 Hz, H-3), 3.78 (d, 1H, ^3^ *J*_2–3_ = 2.1 Hz, H-2), 2.02 (s, 3H, C*H*_3,_ Ac), 1.99 (s, 3H, CH_3,_ Ac); ^13^C­{1H} NMR (150.9 MHz, CDCl_3_): 170.8 (*C*O–Ac), 170.1 (*C*O–Ac), 138.1, 138.0, and 137.8 (C_q_- Ph), 132.3 (2C, *C*H=CH_2_), 128.6, 128,6, 128.4, 128.1, 128.0, 127.9 (C-Ph); 118.7 (CH=*C*H_2_), 118.7 (CH=*C*H_2_), 95.9 (C-1, ^2^ *J*_1,P_ = 6.2 Hz), 78.9 (C-3), 75.2 (CH_2_–OBn), 74.6 (C-2, ^3^ *J*_2,P_ = 8.4 Hz), 74.3 (C-5), 74.1 (C-4); 73.0 (*C*H_2_–OBn), 72.5 (*C*H_2_–OBn), 70.7 (C-6), 69.1 (2C, O*C*H_2_, All. ^3^ *J*_OCH2,P_ = 5.15 Hz), 62.7 (C-7), 21.1 and 20.9 (2 × *C*H_3_, Ac); ^31^P NMR (243.0 MHz, CDCl_3_): - 2.9; HRMS (ESI) *m*/*z* calcd. for [M + H]^+^ C_38_H_46_O_12_P: *m*/*z* = 725.2726, found: *m*/*z* = 725.2713.

### Di-(2,3,4,6-tetra-*O*-acetyl-α-d-*manno*-pyranosyl) triphenylphosphorane (**17**)

^1^H NMR (600 MHz, Tol-d_8_) δ 8.29 (m, 6H, PPh_3_), 7.67 (m, 6H, TPPO), 7.37 (m, 6H, PPh_3_), 7.20 (m, 3H, H-PPh_3_), 6.96–7.10 (m, 24H, TPPO, toluene), 6.45 (br, 2H, NH, DIAD-H_2_), 5.59 (dd, 2H, ^3^ *J*_2,3_ = 2.9 Hz, ^3^ *J*_3,4_ = 10.3 Hz, H-3, H-3′), 5.47 (t, 2H, ^3^ *J*_3,4_ = ^3^ *J*_4,5_ 10.2 Hz, H-4, H-4′), 4.79–4.89 (m, 6H, C*H*-(CH_3_)_2_, DIAD, C*H*-(CH_3_)_2_ DIAD-H_2_), 4.63 (dd, 2H, ^3^ *J*_1,2_ = 2.1 Hz, ^3^ *J*_2,3_ = 2.8 Hz, H-2, H-2′), 4.58 (dd, 2H, ^3^ *J*_1,2_ = 1.8 Hz, ^3^ *J*_1*,P*_ = 10.2 Hz, H-1, H-1′), 4.16 (dd, 2H, ^3^ *J*_6*a*,5_ = 5.4 Hz, ^2^ *J*_*6a,6b*_ = 10.1 Hz, H-6a, H-6a′), 3.91–3.93 (m, 2H, H-5, H-5′), 3.82 (dd, 2H, ^3^ *J*_*6b,5*_ = 2.8 Hz, ^2^ *J*_6*b*,6*a*_ = 12.1 Hz, H-6b, H-6b′), 1.70 (s, 6H, C*H*_3,_ Ac), 1.68 (s, 6H, C*H*_3,_ Ac), 1.63 (s, 6H, C*H*_3,_ Ac), 1.39 (s, 6H, C*H*_3,_ Ac), 1.0 (d, 21H, ^3^ *J*_*CH3‑CH*_ = 6.2 Hz, CH-(C*H*_3_)_2_, DIAD-H_2_), 0.92 (d, 12H, ^3^ *J*_*CH3‑CH*_ = 6.2 Hz, CH-(C*H*_3_)_2_ DIAD); ^13^C­{1H} NMR (150.9 MHz, Tol-d_8_): 170.0 (*C*O–Ac); 169.6 (*C*O–Ac); 169.5 (*C*O–Ac); 169.4 (*C*O–Ac); 169.3 (*C*O–Ac); 132.4, 132.38, 128.7, 128,59 (C-Ph), 92.7 (C-1), 71.6 (C-3), 70.1 (C-2), 69.9 (C-5), 67.2 (C-4), 63.0 (C-6), 21.9 (*C*H_3_, Ac); 21.1 (*C*H_3_, Ac); ^31^P NMR (243.0 MHz, Tol-d_8_): −49 ppm.

### Di-(2,3,4-tri-*O*-benzyl-6,7-di-*O*-acetyl-d-*glycero*-α-d-*manno*-heptopyranosyl) Triphenylphosphorane (**18**)

^1^H NMR (600 MHz, Tol-d_8_) δ 7.86 (m, 6H, H-PPh_3_), 7.73 (m, 10H, TPPO), 7.50 (m, 3H, 3 × CH_2_C_6_ *H*_5_), 7.43 (m, 3H, 3 × CH_2_C_6_ *H*_5_) 7.05–7.30 (m, 70H, PPh_3_, toluene, TPPO, 6 × O–CH_2_C_6_ *H*_5_), 6.52 (br, 2H, NH, DIAD-H_2_), 5.74 (m, 2H, H-6, H-6′), 5.07 (d, 2H, ^2^ *J* = 11.4 Hz, 2 × C*H*_2_C_6_H_5_), 4.85–5.00 (m, 10H, 2 × C*H*_2_C_6_H_5_, C*H*-(CH_3_)_2_ DIAD, C*H*-(CH_3_)_2_ DIAD-H_2_), 4.55–4.65 (m, 6H, 2 × C*H*_2_C_6_H_5_, H-1, H-1′), 4.49 (dd, 2H, ^3^ *J*_6,7a_ = 3.0 Hz, ^2^ *J*_7a,7b_ = 12.1 Hz, H-7, H-7′), 4.08–4.14 (m, 4H, H-4, H-4′, H-7, H-7′), 4.06 (d, 2H, ^2^ *J* = 12.3 Hz, 2 × C*H*_2_C_6_H_5_), 3.95–3.98 (m, 4H, 2 × C*H*_2_C_6_H_5_, H-3, H-3′), 3.86 (dd, 2H, ^3^ *J*_5,6_ = 1.3 Hz, ^3^ *J*_4,5_ = 10.1 Hz, H-5, H-5′), 2.65 (dd, 2H, ^3^ *J*_1,2_ = 1.1 Hz, ^3^ *J*_2,3_ = 1.5 Hz, H2, H-2′), 1.68 (s, 6H, C*H*_3_, Ac), 1.67 (s, 6H, C*H*_3_, Ac), 0.91–1.1 (m, 47H, CH-(C*H*_3_)_2_ DIAD, CH-(C*H*_3_)_2_ DIAD-H_2_); ^31^P NMR (243.0 MHz, Tol-d_8_): - 50 ppm.

## Supplementary Material



## Data Availability

The data underlying this study are available in the published article and its Supporting Information.

## Notes

Additional experimental data, analytical data, and copies of all ^1^H-, ^13^C-, and ^31^P NMR spectra (PDF)
