# Supplementary material for: Stereoselective Anomeric Phosphorylation under Modified Mitsunobu Reaction Conditions
Source: J Org Chem. 2025 Sep 24;90(39):13891–903. doi: 10.1021/acs.joc.5c01760 (PMC12501929; doi:10.1021/acs.joc.5c01760)

Supporting Information

Stereoselective Anomeric Phosphorylation under Modified Mitsunobu Reaction Conditions

Alessandro Monti,[a] Biswajit Sarkar,[a] Alla Zamyatina*[a]

[a] Department of Natural Sciences and Sustainable Resources
Institute of Organic Chemistry, BOKU University
Muthgasse 18, Vienna, A-1190 Austria
E-mail: alla.zamyatina@boku.ac.at

Supporting information summarizes additional studies on the stereoselectivity of phosphorylation under Mitsunobu reaction conditions, synthetic procedures incl. analytical data, and copies of the of ^{1}H-, ^{13}C- and ^{31}P- NMR spectra.

Content

Preliminary studies for anomeric phosphorylation under modified Mitsunobu reaction conditions……………………S1

NMR studies reveal the structure of di(mannosyloxy)triphenylphosphorane **17**…………………………………………………S3

NMR studies reveal the structure of [di-(D-*glycero*-α-D-*manno*-heptosyloxy)]triphenylphosphorane **18**………………S5

Stability of phosphorane **17** in solvents of varying polarity…………………………………………………………………………S8

Stability of phosphorane **18**………S9

Studies on the stereoselectivity of anomeric phosphorylation under modified
Mitsunobu reaction conditions……………………………………………………………………………………………… S10

Auxiliary reagents invert the stereoselectivity of anomeric phosphorylation under
Mitsunobu reaction conditions………………………………………………………………………………………………..S13

Synthetic procedures……S16

References……S19

NMR spectra………S20

Preliminary studies for anomeric phosphorylation under modified Mitsunobu reaction conditions.

First, we found that the preparation of betaine, formed via a Michael-type nucleophilic attack by the triphenyl phosphine on the azodicarboxylate DIAD,[1] must precede the addition of any other reaction components. Following the original procedure consisting of combining lactol **1** with an acidic component (dibenzyl phosphate), triphenyl phosphine and DIAD resulted in no conversion (Table S1, entries 1, 2). Premixing PPh_3, DIAD and dibenzyl phosphate in a solvent (THF, DCM or toluene) with subsequent addition of lactol **1** gave no phosphorylated product (Table S1, entries 5, 8, 14). Consequently, diverse reaction protocols with altered scheme of the sequence of reagent addition[2] were investigated.

Pre-forming betaine (from equimolar quantities of PPh_3 and DIAD) followed by the addition of the lactol **1** and the slow addition of a solution of dibenzyl phosphate in THF (Table S1, entries 3, 4), DCM (entries 6, 7), DMF (entry 10), CH_3CN (entry 11), and toluene (entries 12, 15) at different temperatures (r.t. or -10 °C) resulted in the formation of anomeric phosphate with partial inversion of configuration and yields ranging from 30 to 60%. The anomeric ratio of the final glycosyl phosphate varied from α/β = 1:1 to 1.5:1 and was dependent on the sequence and protocols of reagent addition, for instance, slow addition of the phosphate alone (Table S1, entries 4, 6, 12, 16) vs. slow addition of both the phosphate and lactol **1** (entries 7, 10, 11) to the pre-formed betaine. The overall yield could be increased from 30% (reaction solvent: DCM, entries 6, 7) to 50% (reaction solvents: THF, acetonitrile, DMF, entries 3, 4, 10, 11) and up to 60% using toluene as reaction solvent (entry 12). Reversing the order of addition of lactol **1** and dibenzyl phosphate to the preformed betaine (phosphate first, lactol last) resulted in no transformation (entry 14). Changing the nature of the phosphine[3] did not lead to any improvements (Table S1, entries 16, 17). However, using triethylamine as an additive increased the overall yield to 80% with DCM as the solvent (entry 9), although the stereoselectivity was compromised as substantial amount of the α-configured product was formed (without inversion of configuration, α/β = 3.3:1). With toluene as the solvent, the addition of triethylamine increased the overall yield but did not improve the stereochemical outcome (Table S1, entries 15, 18, 19).

Table S1: Screening of conditions for the phosphorylation of lactol **1** using dibenzyl phosphate under Mitsunobu reaction conditions: the influence of solvent polarity, the nature of phosphine and the sequence of addition of reagents.

entry	phosphine	solvent	pre-formed betaine (Phosphine (eq) + DIAD (eq))	additive	ratio (α/β) (**5**/**4**)	conversion	sequence of reagent addition
1	PPh_3	THF	NO		NO REACTION		(a)
2	PPh_3	THF	NO		NO REACTION		(a2)
3	PPh_3	THF	YES		1:1[(g)]	50%[(h)]	(b)
4	PPh_3	THF	YES		1.5:1	50%[(h)]	(c)
5	PPh_3	THF	NO		NO REACTION		(f)
6	PPh_3	DCM	YES		1:1	30%[(h)]	(c)
7	PPh_3	DCM	YES		1.5:1[(g)]	30%[(h)]	(d)
8	PPh_3	DCM	NO		NO REACTION		(f)
9	PPh_3	DCM	YES	Et_3N	3.3:1[(g)]	80%[(h)]	(c)
10	PPh_3	CH_3CN	YES		1:1[(g)]	50%[(h)]	(d)
11	PPh_3	DMF	YES		1:1[(g)]	50%[(h)]	(d)
12	PPh_3	Toluene	YES		0.7:1	60%[(h)]	(c)
13	PPh_3	Toluene	YES		NO REACTION		(e)
14	PPh_3	Toluene	NO		NO REACTION		(f)
15	PPh_3	Toluene	YES	Et_3N	1.5:1[(g)]	50%[(h)]	(c)
16	P(*m* ClPh)$_3$	Toluene	YES		1:1[(g)]	50%[(h)]	(c)
17	P(*p* CF_3Ph)$_3$	Toluene	YES		1:1[(g)]	50%[(h)]	(c)
18	P(*m* ClPh)$_3$	Toluene	YES	Et_3N	1:1[(g)]	70%[(h)]	(c)
19	P(*p* CF_3Ph)$_3$	Toluene	YES	Et_3N	1:1[(g)]	80%[(h)]	(c)

(a) Phosphine (3 equiv.), $(BnO)_2P(O)OH$ (3 equiv.) and 1 (1 equiv.) were dissolved in a solvent (see Tablle) and mixed at r.t. (a) or (at -50°C, subsequently at r.t.) (a2) and a solution of DIAD (3 equiv.) in a solvent was slowly added over 2 h.

(b) betaine (1.5 equiv.) was preformed from PPh_3 (1.5 equiv.) and DIAD (1.5 equiv.). After 10 min lactol **1** (1 equiv.) in a solvent was added, followed by addition of a solution of $(BnO)_2P(O)OH$ (1.2 equiv.) and a solution of betaine (1.5 equiv.) over 1 h at r.t.

(c) betaine (3 equiv.) was preformed in a solvent (see table) from PPh_3 (3 equiv.) and DIAD (3 equiv.). Subsequently, lactol **1** (1 equiv.) was added followed by a slow addition (over 3 h) of a solution of $(BnO)_2P(O)OH$ (3 equiv.) in a solvent (see table) at -10 °C.

(d) betaine (3 equiv.) was prepared from PPh_3 (3 equiv.) and DIAD (3 eq) in a solvent (see table), then a solution of lactol **1** (1 equiv.) and a solution of $(BnO)_2P(O)OH$ (3 equiv.) were added slowly (over 1 h) at r.t.

(e) betaine (3 equiv.) was preformed from PPh_3 (3 equiv.) and DIAD (3 equiv.) in a solvent (see table), then a solution of $(BnO)_2P(O)OH$ (3 equiv.) in a solvent was added, followed by the addition of a solution of lactol 1 (1 equiv.) at r.t.

(f) PPh_3 (3 equiv.), DIAD (3 equiv.) and $(BnO)_2P(O)OH$ (3 equiv.) were premixed in a solvent, followed by the addition of lactol **1** (1 equiv.) at r.t.

(g) the α/β ratio was determined by analysis of the ^{31}P- and ^{1}H- NMR spectra

(h) the α/β ratio was determined based on the TLC analysis

NMR studies reveal the structure of di(mannosyloxy)triphenylphosphorane 17

The structure of (di-mannosyloxy)triphenylphosphorane **17** obtained from Man-lactol **1** and betaine **15** was confirmed using ^{1}H-NMR and 2-D NMR (^{31}P-^{1}H-HMBC and ^{13}C-^{1}H-HSQC) analyses (Figures S1, S2). Betaine **15** was prepared from DIAD and PPh_3, with concomitant formation of TPPO (**16**) due to partial hydrolysis of the betaine (caused by residual water in toluene-d_8). Addition of Man-lactol **1** led to the formation of phosphorane **17** (Figure S1-A).

A

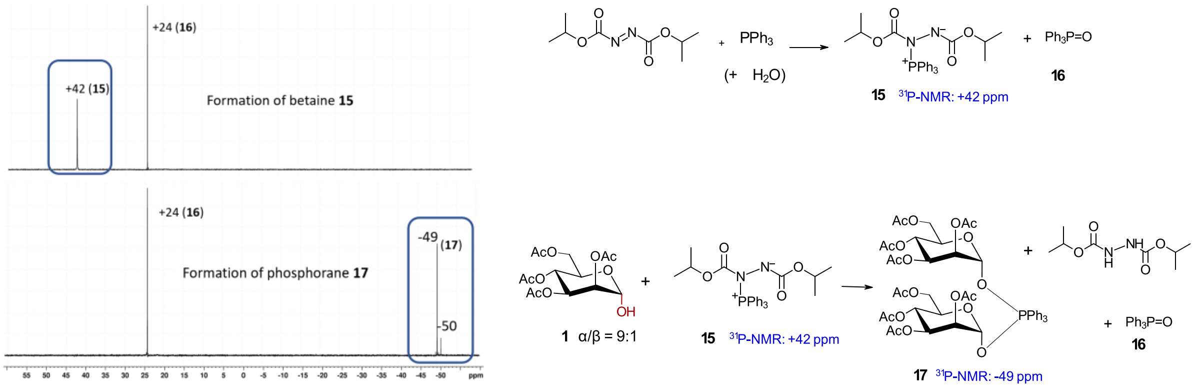

B

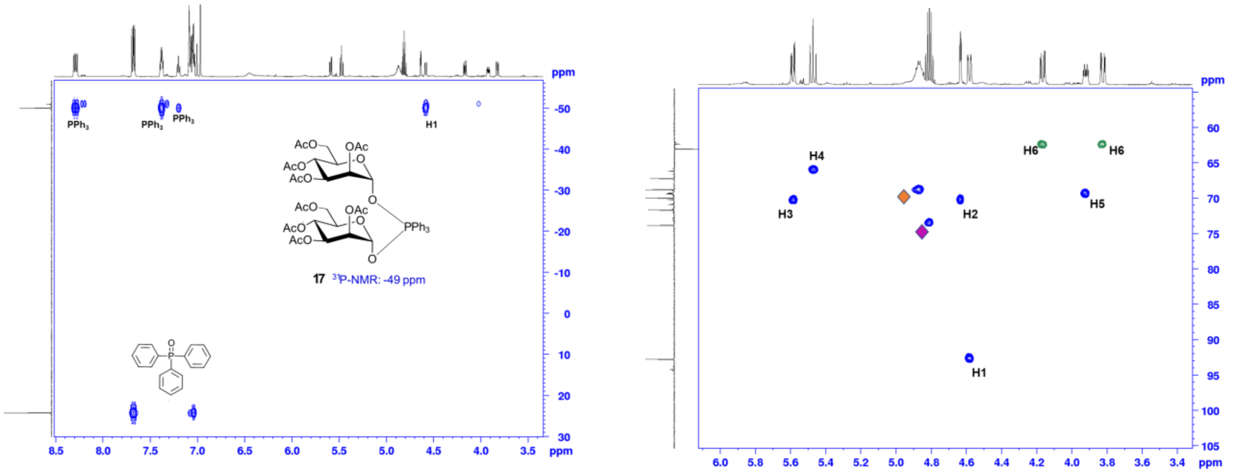

C

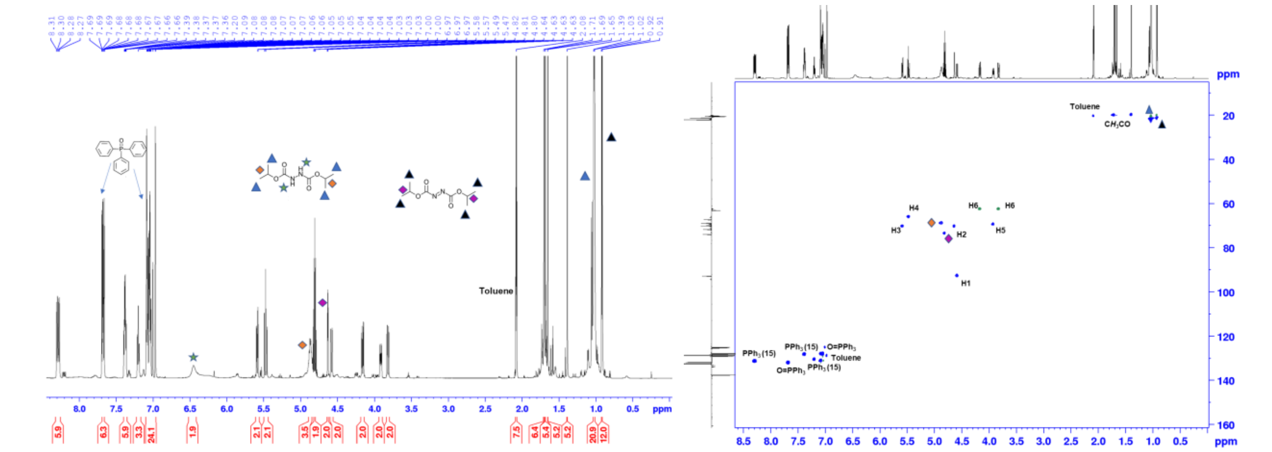

Figure S1. Structural assignment of signals in ^{1}H-NMR spectrum of (di-mannosyloxy)triphenylphosphorane **17**. (A) ^{31}P-NMR spectra reflecting the reaction steps leading to the formation of betaine **15** and phosphorane **17**; (B) ^{31}P-^{1}H HMBC-NMR spectrum and ^{13}C-^{1}H HSQC-NMR spectrum of **17** showing cross-peaks of the Man-H1 and >PPh_3 of **17** as well as those of TPPO; (C) ^{1}H-NMR and ^{13}C-^{1}H HSQC-NMR spectra of **17** showing structural assignments of the signals corresponding to DIAD, DIAD-H_2 and Man.

The ^{1}H-NMR peak of the anomeric proton at δ: 4.58 ppm with $^3J_{1\text{-}2}$ = 2.2 Hz and $^3J_{1\text{-}P}$ = 6.4 Hz correlates with a ^{13}C-NMR peak at δ: 91 ppm (Figure S1-B), with the heteronuclear-coupling constant $J_{1H\text{-}1C}$ = 168 Hz supporting the α-D-*manno*-configuration at the anomeric center.[4] The signals of the -C*H*(CH$_3$)$_2$ corresponding to the unreacted DIAD (used in excess) and DIAD-H$_2$ appear around 4.5 ppm, while the peaks corresponding to the terminal methyl groups -CH(C*H*$_3$)$_2$ of DIAD and DIAD-H$_2$ are observed at δ: 0.9 – 1.2 ppm (Figure S1-C). A comparison of the integrals of the protons of the phenyl groups >P(C$_6$*H*$_5$)$_3$ of phosphorane **17** (15H in the aromatic region) with the integrals of the resonances from the protons of the pyranose ring (each proton showing an integral of 2H), confirms the reaction stoichiometry and the structure of **17**, in which two Man moieties are attached to a single PPh$_3$ residue (Figure S2-A). Other peaks present in the aromatic area are attributed to TPPO (**16**) and toluene (Figure S1-C, S2-A). Betaine **15** was fully consumed, as evidenced by the absence of the ^{1}H-NMR resonances corresponding to betaine in the preparation of **17** (Figure S2-B). Phosphorane **17** appears to be relatively stable on the timescale of the NMR measurements, as no signs of hydrolysis of **17** back to lactol **1** were observed (Figure S2-B).

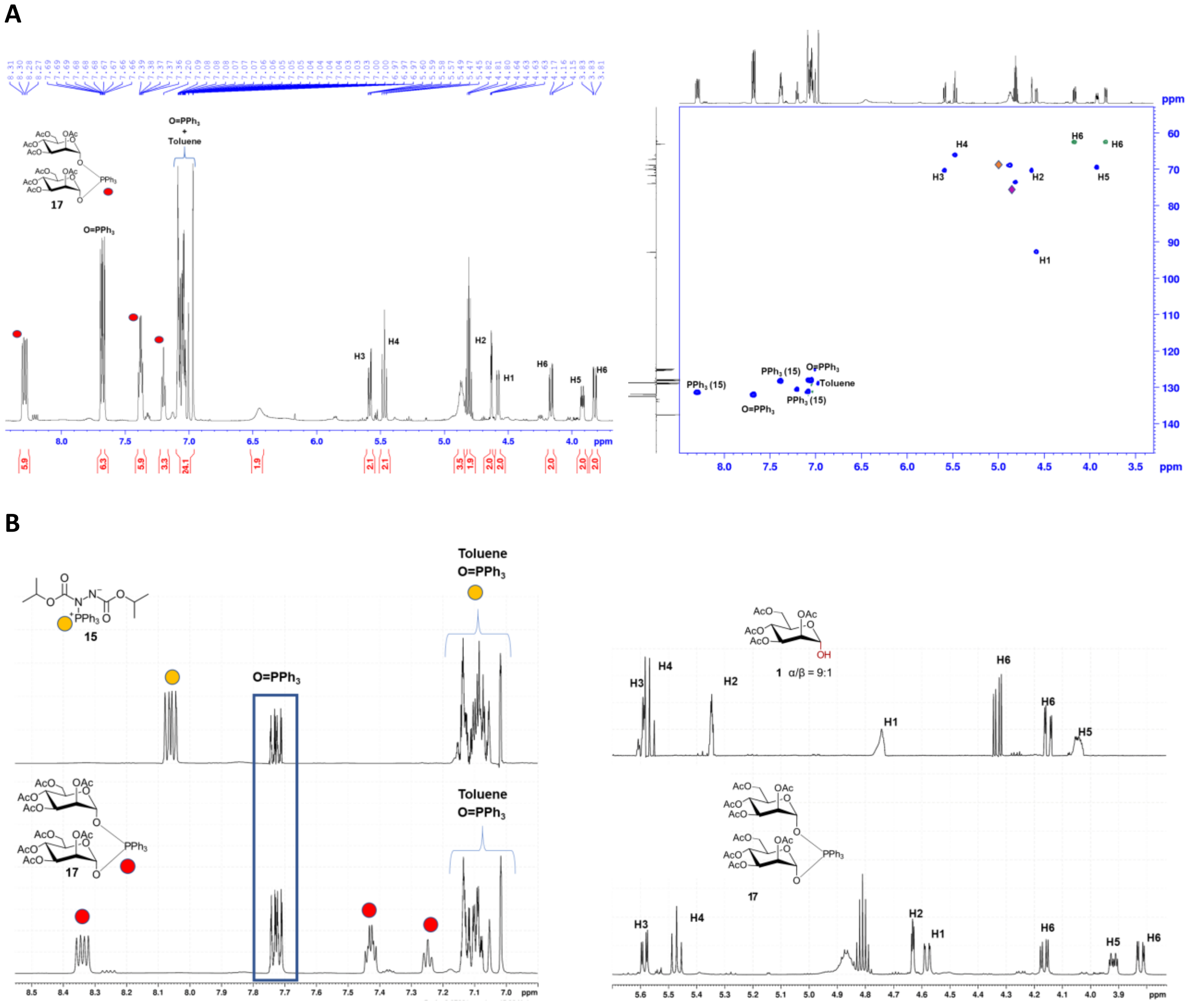

Figure S2. Structural assignment of signals in ^{1}H-NMR spectrum of (di-mannosyloxy)triphenylphosphorane **17**.

(**A**) *Left*: ^{1}H-NMR spectrum of **17** showing the structural assignment of signals corresponding to >PPh$_3$ and Man moieties; *Right*: ^{1}H-^{13}C HSQC-NMR spectrum of **17** displaying cross-peaks corresponding to the aromatic signals of >PPh$_3$ in **17** and TPPO, as well as signals in the carbohydrate region of **17**; (**B**) Structural assignment of signals corresponding to the aromatic and carbohydrate moieties of **17**. *Left*: Superimposed ^{1}H-NMR spectra of betaine **15** (containing TPPO **16** due to partial hydrolysis) and phosphorane **17**, demonstrating that **17** is free of betaine; *Right*: Superimposed ^{1}H-NMR spectra of Man-lactol **1** and phosphorane **17**, confirming the absence of lactol **1** in the NMR-preparation of **17**.

NMR studies reveal the structure of [di-(D-*glycero*-α-D-*manno*-heptosyloxy)]triphenylphosphorane 18.

The structure of (di-heptosyloxy)triphenylphosphorane **18** was confirmed using ^{1}H-NMR, ^{31}P-^{1}H-HMBC- and ^{13}C-^{1}H-HSQC-NMR analysis (Figures S3, S4). As the preparation of **18** in the NMR tube represents a mixture of phosphorane **18** (90%), its β-heptosyl counterpart (approx. 5%), a small amount of β-heptose (approx. 5%, resulting from hydrolysis of β-Hep-**18**) and several reaction by-products: TPPO **16** and DIAD-H_2 (arising from hydrolysis of betaine due to residual water in toluene-d_8 and from the reaction between Hep-lactol **8** and betaine **15**), DIAD (used in slight excess), we performed a comprehensive NMR analysis to quantify the number of equivalents of each reaction component present in the mixture (Table S2). This analysis was crucial, as the resonances of the PPh_3 moiety in **18** overlap with those of TPPO and with the signals from the benzyl groups of the Hep moiety.

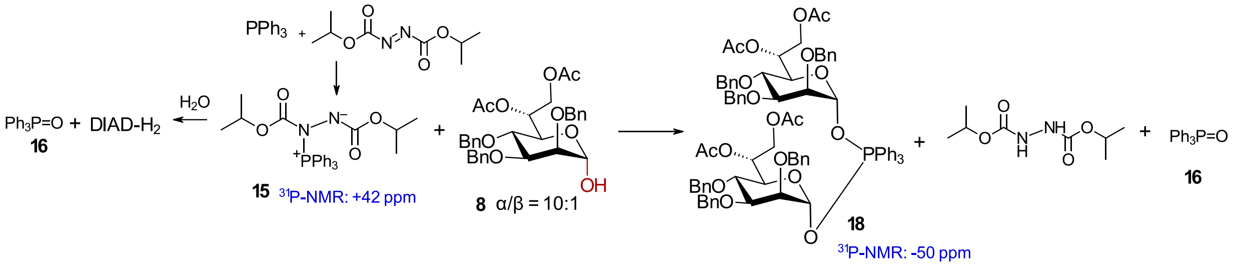

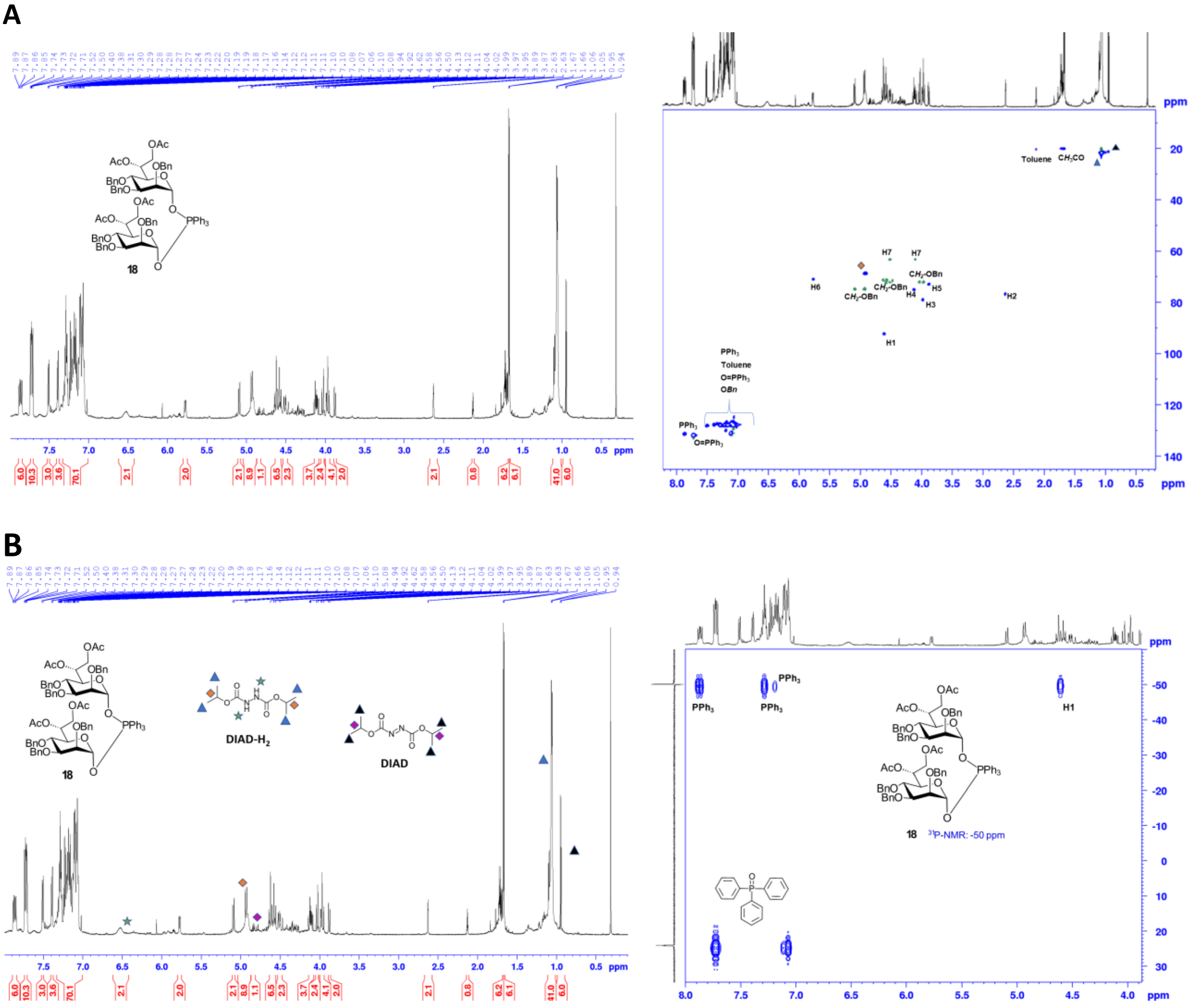

Figure S3. Structural assignment of signals in ^{1}H-NMR spectrum of (di-heptosyloxy)triphenylphosphorane **18**.
(A) ^{1}H-NMR spectrum and ^{1}H-^{13}C HSQC-NMR spectrum of **18**; (B) Structural assignment of signals corresponding to the unreacted DIAD and to DIAD-H_2 and ^{31}P-^{1}H HMBC-NMR spectrum of **18** showing the cross-peaks corresponding to the >PPh_3 moiety of **18** and to TPPO (**16**).

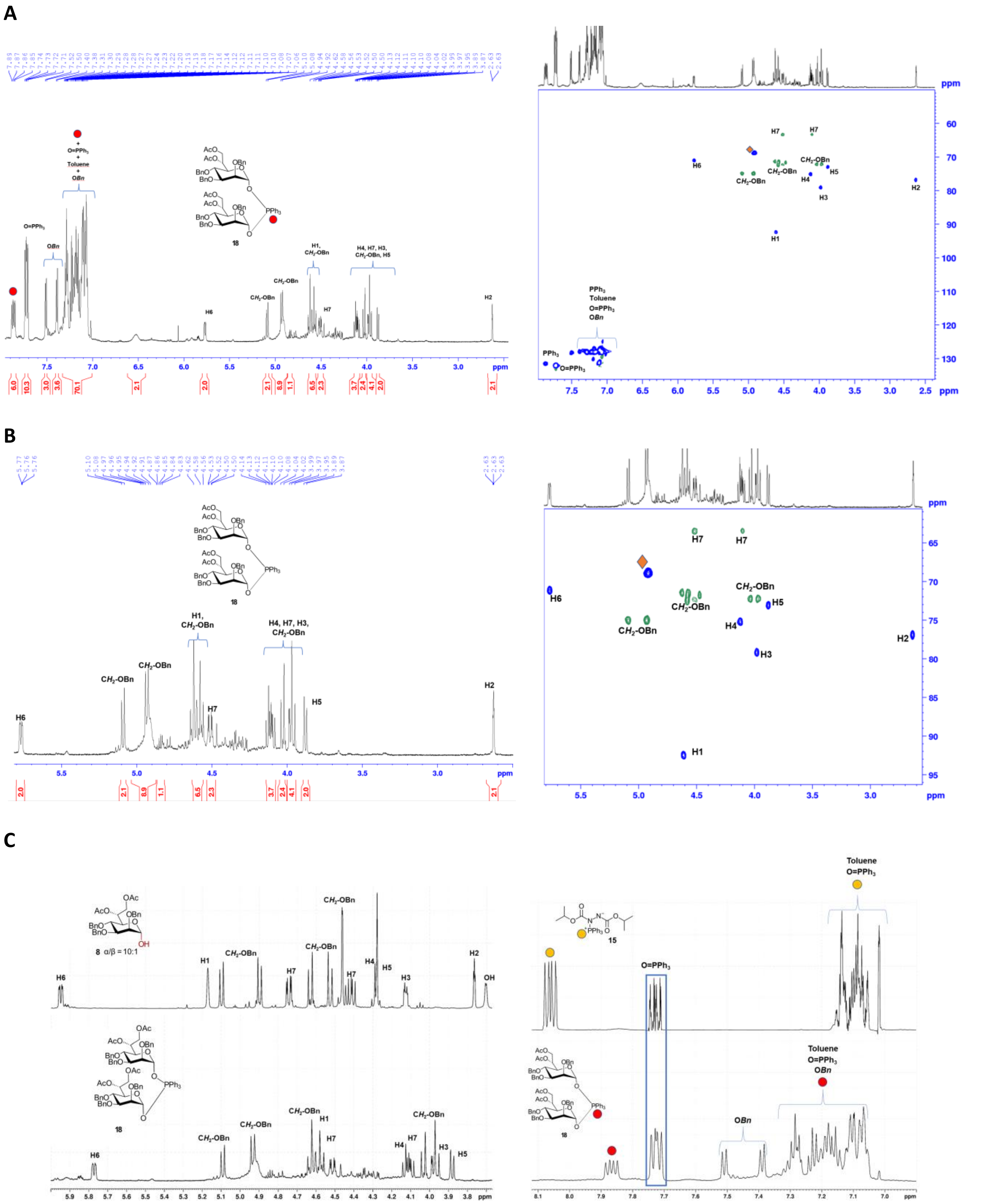

Figure S4. Structural assignment of signals in ^{1}H-NMR spectrum of (di-heptosyloxy)triphenylphosphorane **18**.
(**A**) ^{1}H-NMR spectrum of **18** and ^{1}H-^{13}C HSQC-NMR spectrum of **18** showing assignments in the carbohydrate and aromatic regions. (**B**) Structural assignment of signals corresponding to the heptosyl moieties of **18**. (**C**) Right: A superimposition of the ^{1}H-NMR spectrum of betaine **15** (contains TPPO **16** due to partial hydrolysis) and ^{1}H-NMR spectrum of **18** showing that the preparation of **18** is betaine-free; Left: A superimposition of the ^{1}H-NMR spectrum of Hep-lactol **8** and phosphorane **18** showing the presence of residual signals of **8** (α/β mixture) and demonstrating insufficient stability of **18**.

A comparison of the integrals of the phenyl groups in PPh_3 of TPPO with the calculated integrals for the aromatic protons of the PPh_3 moiety in **18** (15 H), and with the integrals of the heptose protons H6, H7, H5, and H2 (each integrating to 2H) confirmed the structure of **18** as (di-D-*glycero*-α-D-*manno*-heptosyloxy)phosphorane (Table S2). The resonance corresponding to the anomeric proton was identified using ^{1}H-NMR, ^{31}P-^{1}H-HMBC and the ^{13}C-^{1}H-HSQC NMR analysis. The anomeric proton of **18** appears at δ: 4.6 ppm with heteronuclear coupling constant $J_{C1\text{-}H1}$ = 168 Hz confirming the α-Hep configuration of **18**.

Table S2. Integral calculation for structural assignment of the ^{1}H-NMR spectrum of **18**

Region (ppm)	Integral TPPO (**16**)	Toluene (residual solvent	>PPh_3 of **18**	3×(CH_2-C_6***H***$_5$) of **18**	H1, H3, H4, H4, 2×H7 of Hep in **18**	Integral DIAD	Integral DIAD-H_2
6.90 – 8.32 Total Integral 92 H	2.4 equiv. 2.4×15=36H	(C_6*H*$_5$) 6 H	1 equiv. 15 H	2 equiv. 30 H		-	-
4.50 – 5.60	-	-	-		2 equiv.Hep 12 H each 2 H	0.5 equiv. 1 H -C*H*-(CH_3)$_2$	3.4 equiv. 7 H **-C*H*-(CH_3)$_2$**
2.08	-	0.8 H C*H*$_3$				-	-
0.8 – 2.50	-	1.1 equiv. 0.8 H (CH_3)	-	-	-	0.5 equiv. 6 H -CH-(C*H*$_3$)$_2$ corresponds to	3.4 equiv. 41 H -CH-(C*H*$_3$)$_2$ corresponds to
equiv. calculated based on the integration	2.4 equiv. (hydrolysis of betaine) **1 equiv.** (reaction **15**+**8**)		**1 equiv**.	**2 equiv.**	**2 equiv.**	0.5 equiv. (used in excess)	2.4 equiv. (hydrolysis of betaine) **1 equiv.** (reaction **15**+**8**)

The aromatic region (6.9–8.32 ppm) contains signals from TPPO, the aromatic protons of phosphorane **18**, and a residual signal from toluene, with a total integral of **92 H**. To accurately determine the quantity of aromatic protons in **18**, and the amount of TPPO **16** (formed both from the partial hydrolysis of betaine due to residual water in toluene-d_8, and from the reaction of lactol **8** with betaine **15**) following calculations were performed:

- The integral corresponding to the aromatic protons of toluene (6 H), determined from the integration of the methyl group of toluene (δ: 2.10 ppm, 1 H), which reflects 1 equiv. of toluene, must be subtracted.
- According to the ^{31}P-^{1}H HMBC-NMR and ^{1}H-NMR spectra of phosphorane **18**, approx. 3.4 equiv. of TPPO **16** are present in the mixture. This was determined from the integrals of the isopropyl residue of DIAD-H_2: **-CH-(C*H*$_3$)$_2$** (41 H, 3.4 equiv.) and **-C*H*-(CH_3)$_2$** (7 H, 3.4 equiv.)
- The TPPO **16** (3.17 equiv.) is formed from two sources: 2.4 equiv. result from the hydrolysis of betaine **15** and 1 equiv. is formed in the reaction of betaine with lactol **8**.
- The aromatic protons of **18** are assigned to 1 equiv. of >PPh_3 (15 H) and 2 equiv. of 2,3,4-tri-*O*-benzylated heptose (30 H), residual 5 H are in the aromatic area attributed to benzyl groups of heptose lactol **8** (5%, α/β mixture), and to the β-counterpart of **18** (this is supported by corresponding signals in the carbohydrate region).

The integration of resonances corresponding to the pyranose protons H6, H7, H5, and H2, along with the calculated integrals for the aromatic protons of the PPh_3 moiety of compound **18**, clearly confirms a **1:2** stoichiometry (**1** part **PPh_3** to **2** parts **Hep**).

Stability of phosphorane 17 in solvents of varying polarity

The stability of (di-mannosyloxy)phosphorane **17**, generated in the NMR tube by reaction of a preconditioned betaine **15** (1 equiv.) with Man-lactol **1** (2 equiv.) in various solvents, was studied using ^{31}P-NMR spectroscopy. After the formation of **17** in CD_2Cl_2 was confirmed by ^{31}P-NMR (0 min), the phosphorane rapidly decomposed, becoming undetectable within 10 min. (Figure S5-A). The use of a more polar solvent, CD_3CN did not improve stability, as the ^{31}P-NMR peak of phosphorane **17** was similarly undetectable 10 min after its initial formation (Figure S5-B).

In contrast, (dimannosyloxy)phosphorane **17** generated using a similar procedure in toluene-d_8 exhibited the highest stability. The compound remained intact during the first 3 h after formation, followed by gradual decomposition to Man-lactol **1**, with approximately 20% decomposition observed after 4 h (Figure S6). After 6 h from initial detection, **17** had fully decomposed to lactol **1** and TPPO (Figure S6-B).

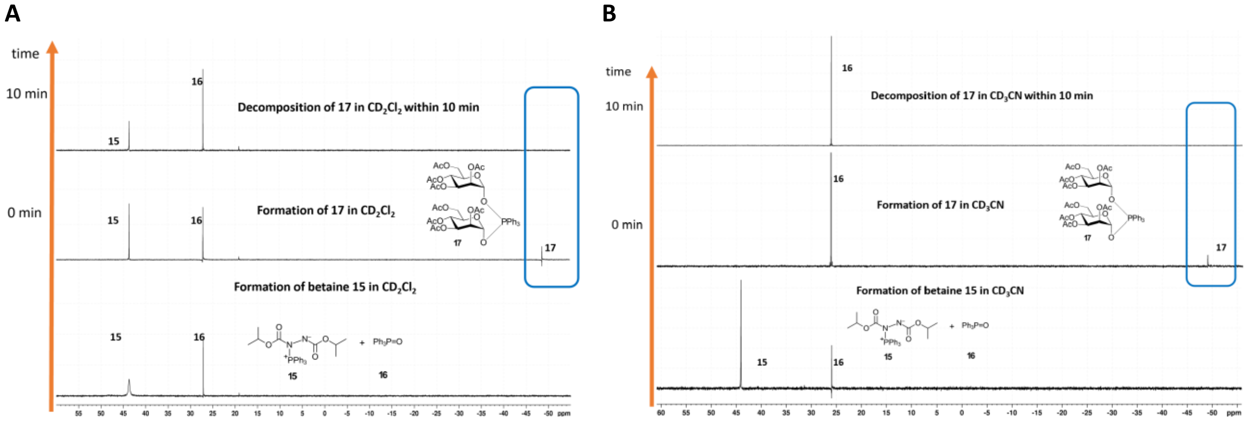

Figure S5. Stability of phosphorane **17** in different solvents: (**A**) in CD_2Cl_2; (**B**) in CD_3CN

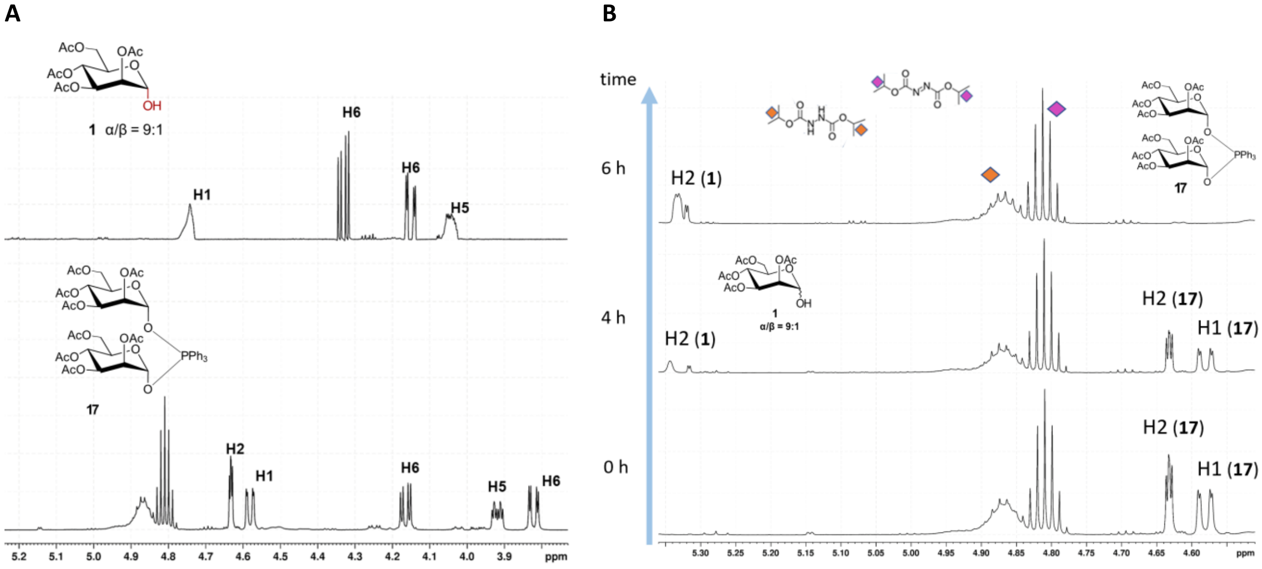

Figure S6. Stability of phosphorane **17** in toluene-d_8. (**A**) Superimposition of ^{1}H-NMR spectra of Man-lactol **1** and phosphorane **17**; (**B**) Phosphorane **17** is stable in toluene-d_8 during the first 3 h after detection and fully decomposes within 6 h of formation.

Stability of phosphorane 18

The stability of (di-heptosyloxy)phosphorane **18** in toluene-d_8 was somewhat reduced due to the presence of electron-donating protecting groups (2,3,4-tri-*O*-benzyl), which activate the anomeric center and render the phosphorane more susceptible to hydrolysis and decomposition. Only about 30% of phosphorane **18** remained intact within 3 h after formation, and the reactive species was no longer detectable by ^{31}P-NMR after 4.5 h (Figure S7).

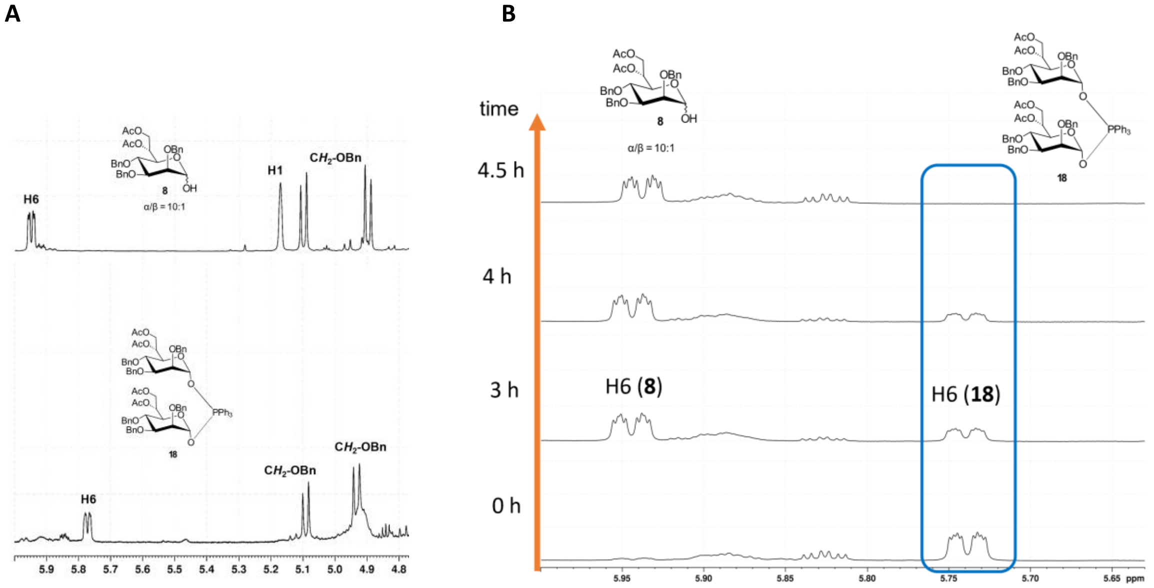

Figure S7. Stability of phosphorane **18** in toluene-d_8.
(**A**) Superimposition of ^{1}H-NMR spectra of Hep-lactol **8** and phosphorane **18**; (**B**) Phosphorane **18** is moderately stable in toluene, as it slowly collapses within the first 3 h after detection to form lactol **8** and TPPO, and fully decomposes within 4.5 h of its formation.

Studies on the stereoselectivity of anomeric phosphorylation under modified Mitsunobu reaction conditions.

Given the limited stability of phosphorane **17** in the presence of excess phosphoric acid diester, which explains the poor reaction outcome when using 3 equiv. of phosphoric acid, it became clear that the reactive intermediate **17** must be continuously regenerated from lactol **1** and betaine. This implies the use of limited amounts of phosphoric acid diester, sufficient to form mannosyl phosphate from **17**, but not to react with betaine to form the unreactive phosphonium salt **15-H**.

When (dimannosyloxy)phosphorane **17**, generated from betaine (3 equiv.) and lactol **1** (3 equiv.) (Figure S8 (I, ii)), was treated with a limited amount of diphenyl phosphate (1.2 equiv.), the ^{31}P-NMR analysis after 1 h revealed the formation of anomeric phosphates **2** and **3** in a β/α ratio of 3:1 (Figure S8 (iii)). At this point, both phosphorane **17** and betaine **15** remained in the reaction mixture, while no signal was observed at δ = +51 ppm for phosphonium salt **15-H**, indicating that the amount of phosphoric acid present was sufficient to convert **17** into glycosyl phosphates, but not enough to protonate betaine. (Figure S8 (iii)). This suggests that the recycling of **17** from betaine **15** and lactol **1** proceeds more rapidly than the protonation of betaine by the phosphoric acid diester. To improve the conversion of lactol **1**, the reaction was extended by an additional 3 h, leading to complete consumption of the starting material and predominant formation of β-mannosyl phosphate **2** (**2/3**=β/α=3:1) (Figure S8 (iv)).

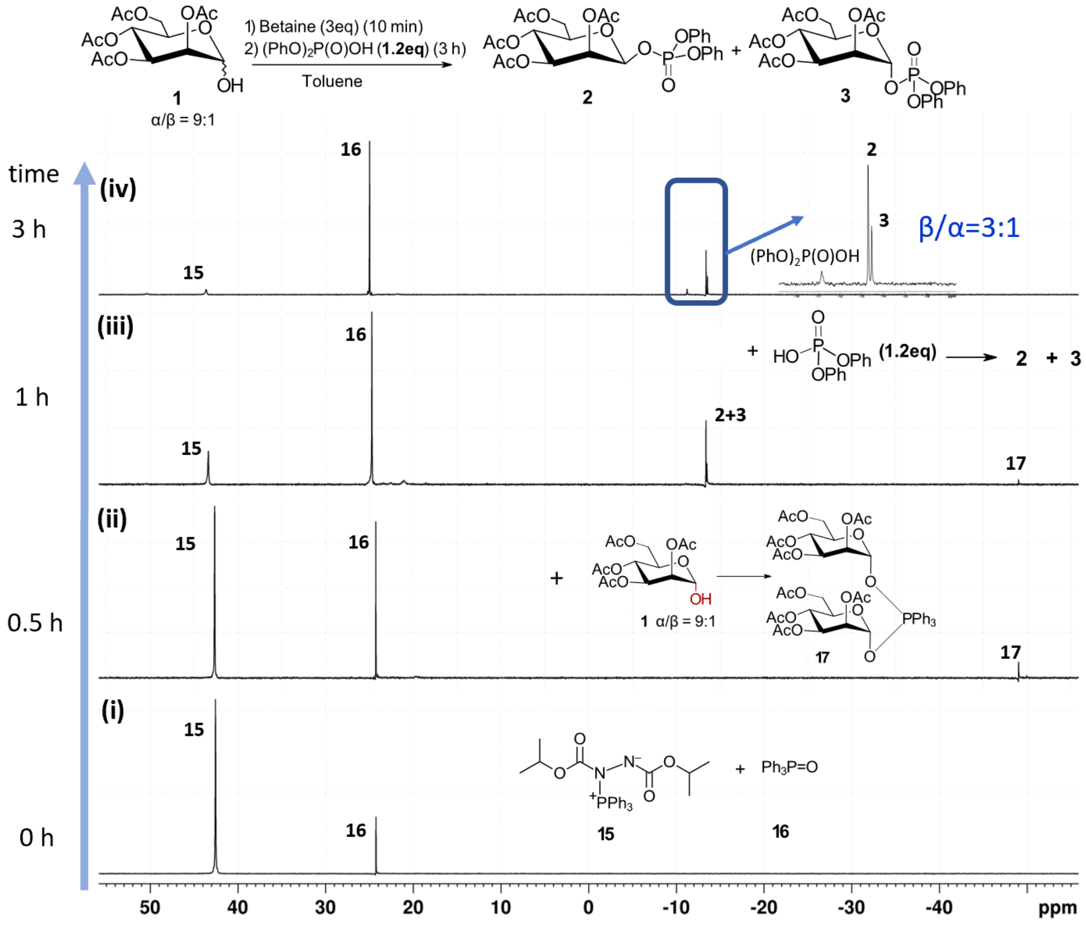

Figure S8. Stereoselectivity of the phosphorylation of Man-lactol **1** under Mitsunobu reaction conditions (using diphenyl phosphate as an acidic component) followed by ^{31}P-NMR spectroscopy in toluene-d_8 (reaction protocol *MIP-inversion*)
(i) Formation of betaine **15**; (ii) generation of phosphorane **17** from Man-lactol **1** and betaine **15**; (iii) generation of mannosyl phosphates upon addition of 1.2 equiv. of diphenyl phosphate; (iv) progression of the phosphorylation reaction, predominantly affording β-mannosyl phosphate **2** with inversion of configuration.

To gain a better understanding of the reaction kinetics and to investigate whether using less bulky protecting groups on the phosphorus atom of phosphoric acid would promote a more pronounced shift toward an SN2 reaction mechanism, diallyl phosphate was employed as the acidic component in the phosphorylation reaction, carried out in an NMR tube using toluene-d_8 as the solvent (Figure S9). The preformed betaine **15** (3 equiv.) (Figure S9 (i)) was reacted with lactol **1** (β/α=9:1) to generate (di-α-mannosyloxy)triphenylphosphorane **17** (Figure S9 (ii)). Upon addition of a limited amount of diallyl phosphate (1.2 equiv., ^{31}P-NMR, δ: -0.5 ppm), rapid formation of β- and α-mannosyl phosphates **6** (^{31}P-NMR, δ: -1.5 ppm) and **7** (^{31}P-NMR, δ: -2.5 ppm) with β/α ratio of 4:1 was observed (Figure S9 (ii)). At this stage, both betaine **15** and (di-α-mannosyloxy)phosphorane **17** remained in the mixture, along with a small amount of phosphonium salt **15-H**. As the overall conversion of the lactol **1** was low, an additional amount of diallyl phosphate (1.2 equiv.) was added, resulting in the predominant formation of α-mannosyl phosphate **7** (Figure S9 (iii)), accompanied by simultaneous consumption of betaine and disappearance of **17** (Figure S9 (iv)).

Whether the observed change in stereochemistry (a shift in the β/α ratio from 4:1 to 2:3) was driven by an SN1-type mechanism (i.e., phosphoric acid-promoted glycosylation reaction proceeding via formation of an oxocarbenium ion) or by anomerisation could not be conclusively determined. However, this experiment confirmed that the continuous presence of betaine, capable of reacting with lactol **1** to regenerate (di-α-mannosyloxy)phosphorane **17**, is essential for promoting the SN2 reaction pathway, which leads to the formation of β-mannosyl phosphate with inversion of configuration. Accordingly, the reaction mixture was supplemented with additional betaine **15** (2 equiv.) (Figure S9 (v)), resulting in the formation of β-mannosyl phosphate **6** as the major product and improving the final β/α ratio of anomeric glycosyl phosphates to 3:1 (Figure S9 (v)). However, under these conditions, lactol **1** was not completely consumed, which led to a reduction in overall yield.

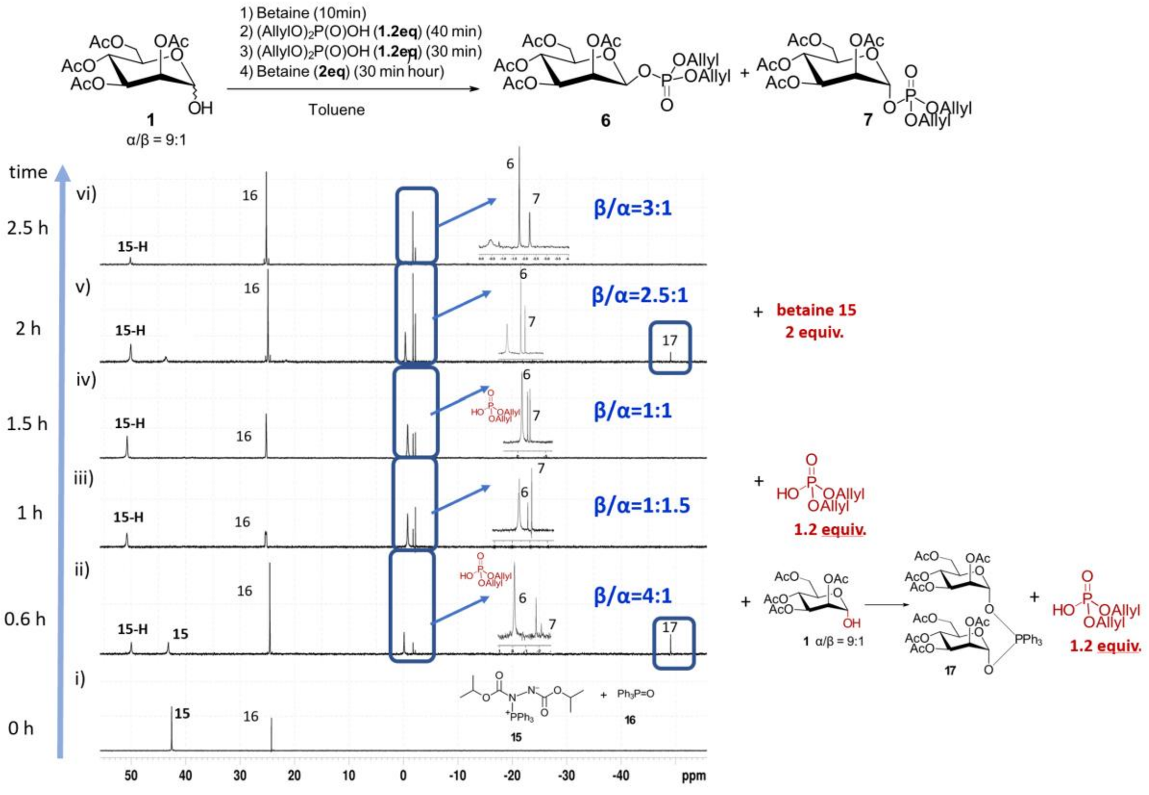

Figure S9. Stereoselectivity of the phosphorylation of Man-lactol **1** under Mitsunobu reaction conditions (using diallyl phosphate as an acidic component) as monitored by ^{31}P-NMR in toluene-d_8.
(i) Formation of betaine **15**; (ii) generation of phosphorane **17** from Man-lactol **1** and betaine **15** followed by phosphorylation with diallyl phosphate (iii) generation of mannosyl phosphates **6** and **7** after addition of the extra 1.2 equiv. of diphenyl phosphate; (iv) moderate stereoselectivity in the absence of betaine; (v) progress of reaction after addition of betaine (2 equiv.); (vi) formation of the major product: β-mannosyl phosphate **6**.

Enhanced yield and improved stereoselectivity were achieved by adding a limited amount of dibenzyl phosphate to the mixture containing both betaine **15** and phosphorane **17** (Figure S10 (ii)). This led to the formation of β-mannosyl phosphate **6** with an β/α ratio of 2:1 (Figure S10 (iii)), while both betaine **15** and phosphorane **17** remained detectable. After 2 h of reaction, upon complete consumption of the starting lactol **1**, the β/α ratio of the resulting mannosyl phosphates increased to β/α=3.5:1 (Figure S10 (iv)).

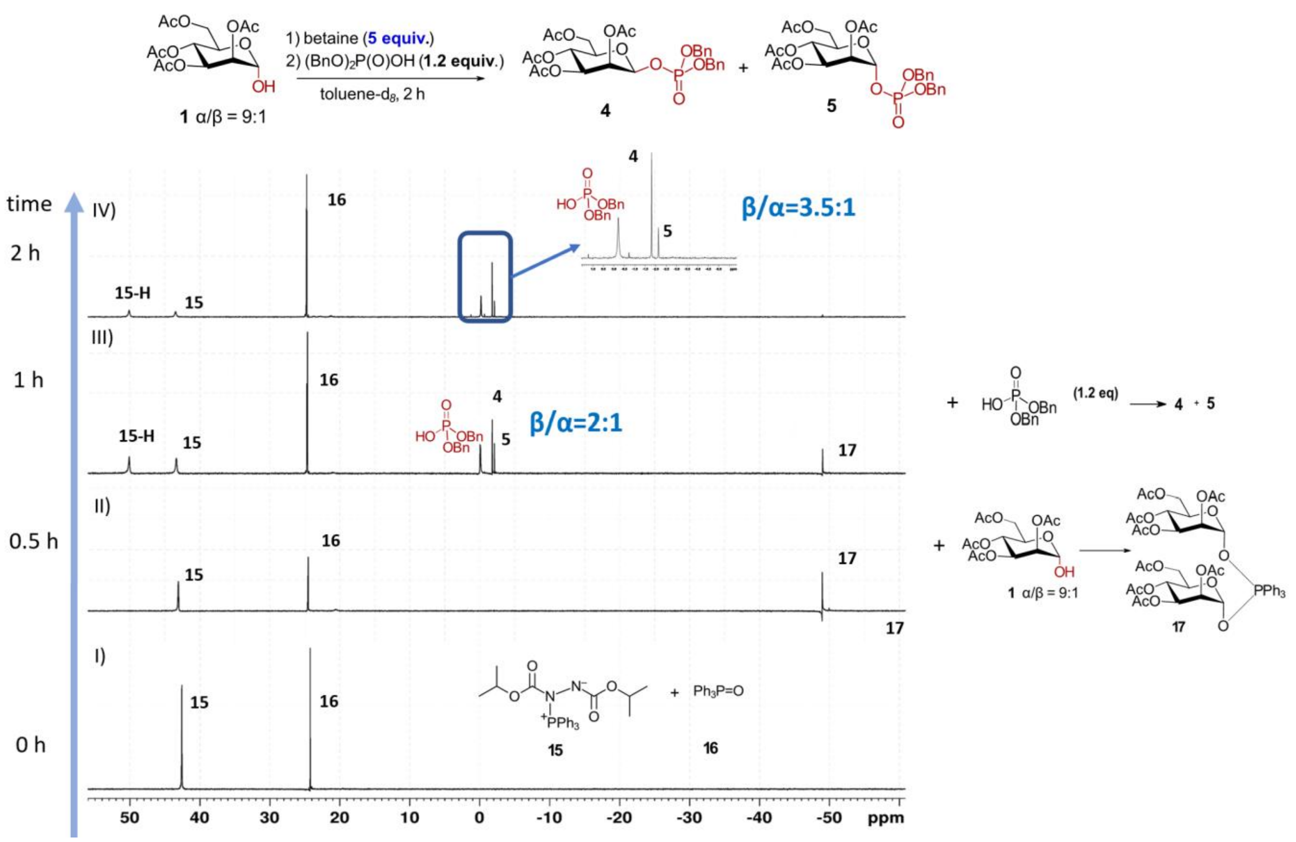

Figure S10. Stereoselectivity of the phosphorylation of Man-lactol **1** under Mitsunobu reaction conditions (using dibenzyl phosphate as an acidic component) as monitored by ^{31}P-NMR in toluene-d_8 (reaction protocol *MIP-inversion*)
(i) Formation of betaine **15**; (ii) generation of phosphorane **17** from Man-lactol **1** and betaine **15**; (iii) generation of mannosyl phosphates **4** and **5** after addition of 1.2 equiv. of dibenzyl phosphate; (iv) progress of reaction with formation of the major product: β-mannosyl phosphate **4**.

To establish reaction conditions for the stereoselective formation of β-heptosyl phosphates, Hep-lactol **8** was used as the nucleophilic component and diallyl phosphate as the acidic component (Figure S11). Betaine **15** (3 equiv.) was preconditioned in the NMR tube (Figure S11 (i)) and subsequently replenished with lactol 8 (β/α = 10:1), which gave (di-α-heptosyloxy)triphenylphosphorane **18** (Figure S11 (ii)). As proof of concept, an equimolar amount of diallyl phosphate (3 equiv., ^{31}P-NMR, δ: -2 ppm; (Figure S11 (iii)) was added to the mixture, which, as expected, resulted within 2 h in the predominant formation of α-heptosyl phosphate **14** (β/α=1:2) and concomitant formation of phosphonium salt **15-H** (Figure S11 (iv)). This experiment confirmed previous conclusions for the β-stereoselective phosphorylation under Mitsunobu conditions using tetraacylated mannose lactol **1**. In the following experiments, using Hep lactol **8** as the nucleophilic component and dibenzyl or diphenyl phosphates as the acidic components, the focus was on supporting the continuous regeneration of phosphorane **18** (from lactol **8** and betaine **15**), while keeping the amount of acidic component to a minimum (Figure 4).

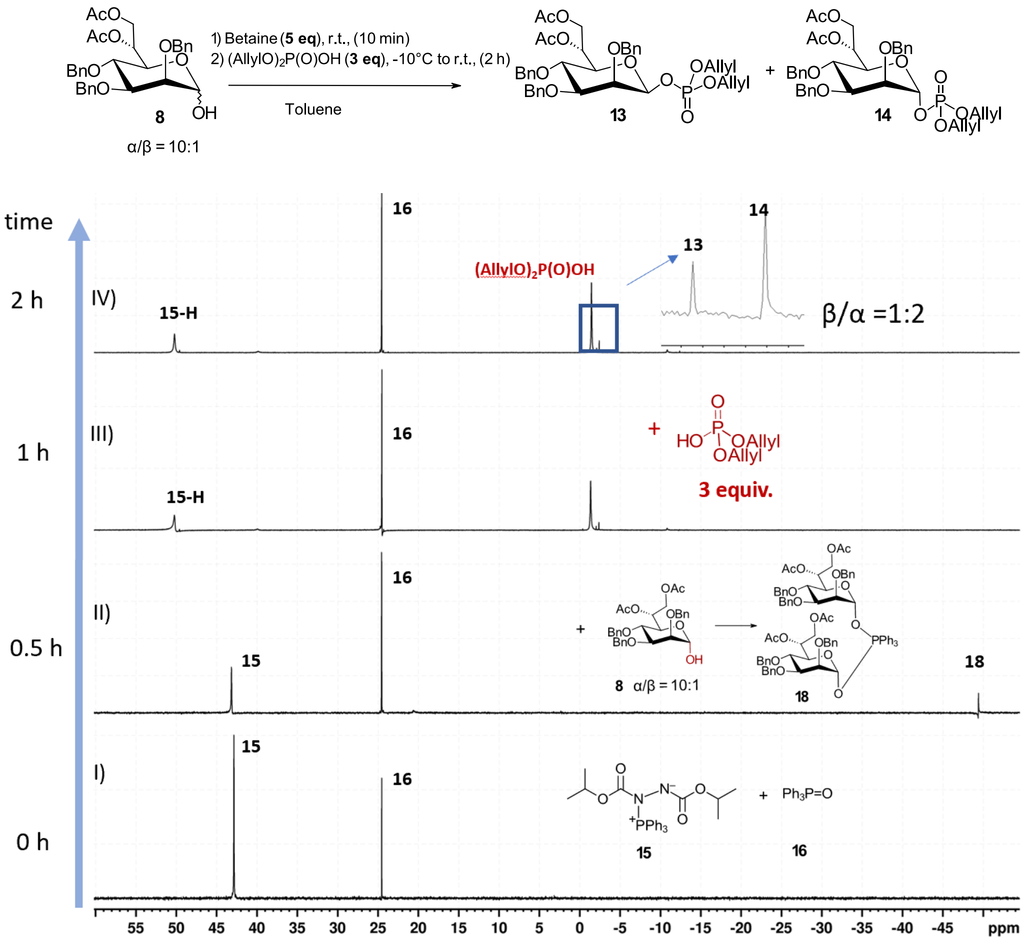

Figure S11. Stereoselectivity of the phosphorylation of Hep-lactol **8** under Mitsunobu reaction conditions (using diallyl phosphate as an acidic component) as monitored by ^{31}P-NMR in toluene-d_8.
(i) Formation of betaine **15**; (ii) generation of phosphorane **18** from Hep-lactol **8** and betaine **15**; (iii) generation of heptosyl phosphates **13** and **14** after addition of 3 equiv. of diallyl phosphate; (iv) progress of reaction with formation of the major product: α-heptosyl phosphate **14**.

Auxiliary reagents invert the stereoselectivity of anomeric phosphorylation under Mitsunobu reaction conditions.

Because all reported methods for carbohydrate phosphorylation under modified Mitsunobu conditions rely on an auxiliary reagent, most commonly Et_3N, to accelerate the reaction rate and improve overall conversion, we investigated the role of additives in the reaction mechanism using ^{31}P NMR spectroscopy. As supported by both literature data and our initial experiments (Table S1), the transformation in the presence of auxiliary reagents generally exhibits poor stereoselectivity, yielding a mixture of anomeric phosphates formed with both inversion and retention of configuration. To clarify the effect of auxiliary reagents, we carried out the anomeric phosphorylation of lactols **1** and **8** using diphenyl phosphate as the acidic component and Et_3N or DMAP as an additive. After conducting several preliminary experiments, we hypothesized that the use of Et_3N or DMAP shifts the mechanism toward phosphorylation with retention of configuration (Figure S12). In order to favour the formation of glycosyl phosphates with retention of anomeric configuration the formation of phosphorane **17** was bypassed by altering the order of reagent addition. Rather than premixing betaine with lactol **1** to produce phosphorane **17**, all the reagents – lactol, protected phosphoric acid and DMAP – were added simultaneously to the preformed betaine **15** giving rise to β/α = 1:1 mixture of mannosyl phosphates (Figure S12-A). Given that DMAP is known to promote rapid anomerisation of α-Man-lactol **1** to its β-anomer, we investigated imidazole as an alternative auxiliary reagent. This completely shifted the reaction mechanism toward phosphorylation with retention of configuration, resulting in the predominant formation of α-configured mannosyl phosphate **3** (**2/3** = β/α = 1:5) (Figure S12-B).

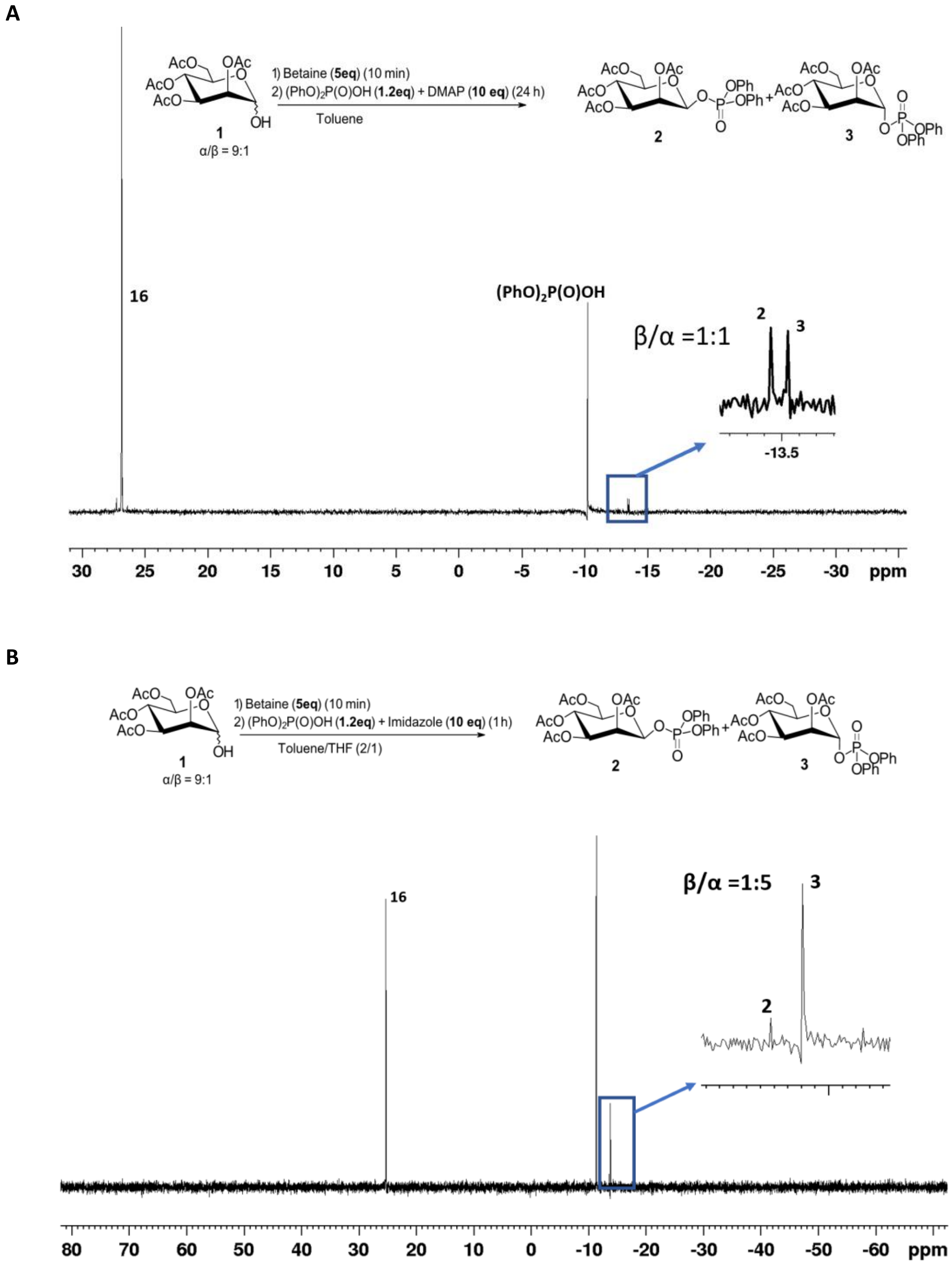

Figure S12. Influence of auxiliary reagents on the stereoselectivity of the phosphorylation of Man-lactol **1** under Mitsunobu reaction conditions, ^{31}P-NMR in toluene-d_8. (**A**) The use of DMAP as an auxiliary reagent leads to the loss of stereoselectivity; (**B**) The application of imidazole as an auxiliary reagent results in anomeric phosphorylation with retention of configuration and excellent stereoselectivity (reaction protocol *MIP-retention*).

Applying similar reaction conditions (reaction protocol *MIP-retention*) to the anomeric phosphorylation of Hep-lactol **8** afforded α-heptosyl phosphate **10** as the major product, along with the minor amount of its β-counterpart **9** (Figure S13).

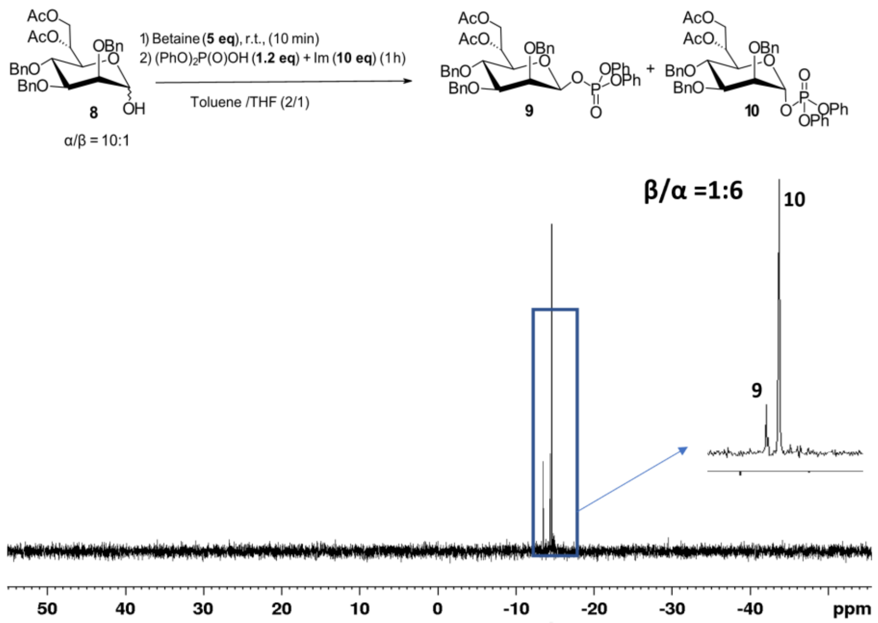

Figure S13. Influence of auxiliary reagents on the stereoselectivity of anomeric phosphorylation of Hep-lactol **8** under modified Mitsunobu reaction conditions, ^{31}P-NMR in toluene-d$_8$. The use of imidazole as an auxiliary reagent leads to phosphorylation with retention of configuration and excellent stereoselectivity (reaction protocol *MIP-retention*).

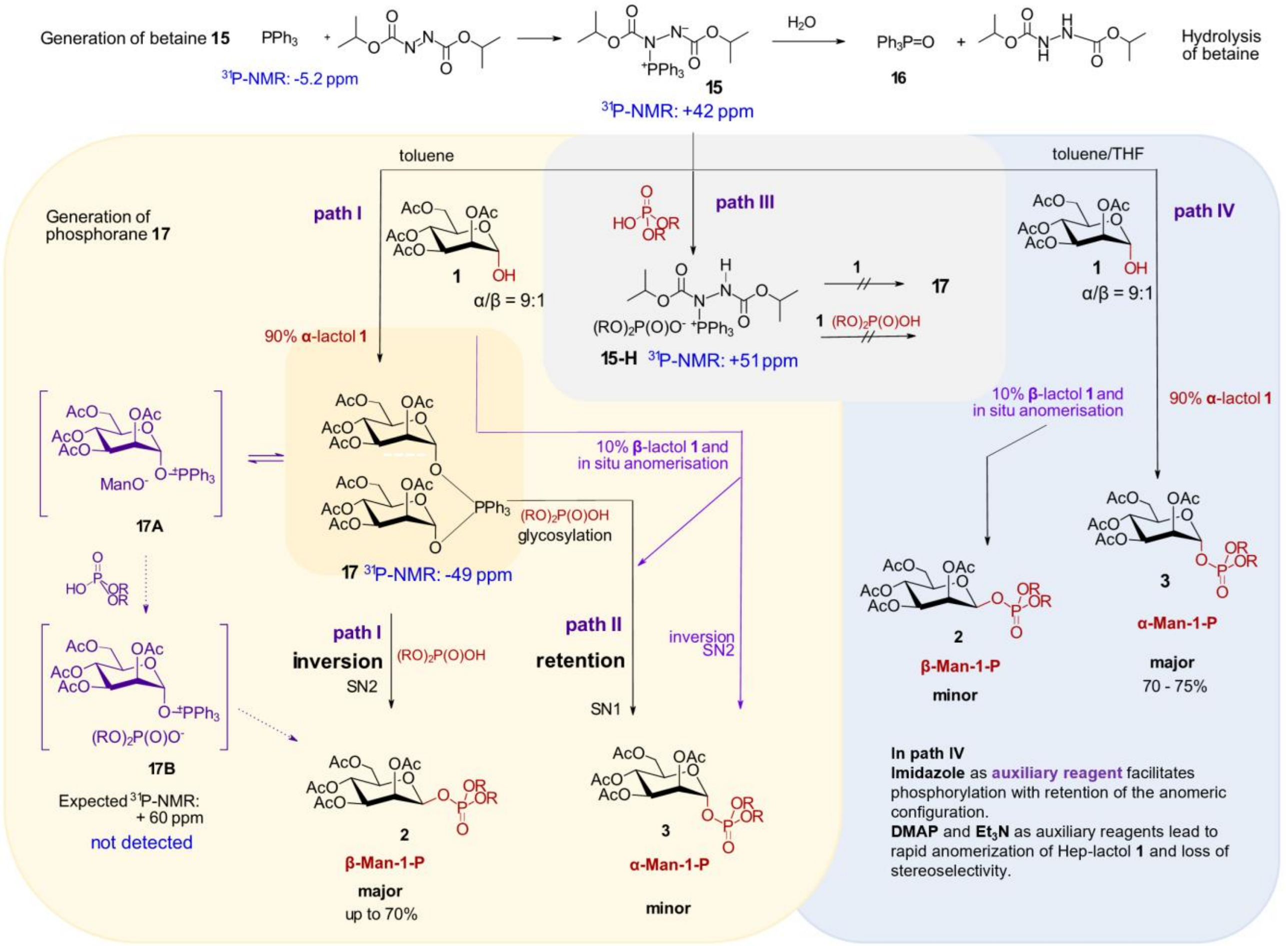

Scheme S1. Phosphorylation under modified Mitsunobu reaction conditions with α-D-Man lactol **1** as nucleophile: reaction pathways.

Synthetic Procedures

Synthesis of 8

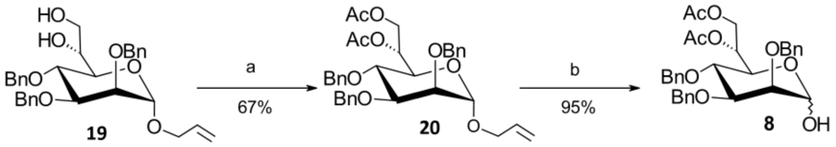

Reagents and conditions: (a) Ac_2O, DMAP, Py, rt; (b) $[Ir(COD)(Ph_2MeP)_2]PF_6$, H_2, THF, then I_2, THF-H_2O (2:1).

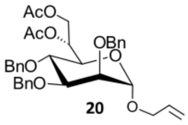

Allyl 2,3,4-tri-*O*-benzyl-6,7-di-*O*-acetyl-D-glycero-D-*manno*-heptopyranose (20)

To a stirred solution of **19**[5] (1.229 mmol, 640 mg) in pyridine (30 ml), DMAP (25 mg) and Ac_2O (0,30 ml) were added and the reaction mixture was stirred for 16 h at r.t.. The mixture was concentrated and the residue was purified by column chromatography on silica gel (toluene/EtOAc, 9:1) to give **20** (500 mg, 0.822 mmol, 67%) as a white amorphous solid. R_f = 0.5 (toluene - EtOAc, 8:2); $[\alpha]^{20}_D$ = +5.00 (c = 0.1, $CHCl_3$); ^{1}H NMR (600 MHz, $CDCl_3$) δ 7.32 (m, 15H,Ph), 5.84 (m, 1H, C*H*-Allyl), 5.54 (m, 1H, H6), 5.21 (dd, 1H, $^2J_{=CH2cis,=CH2trans}$ = 1.6 Hz,$^3J_{CH=,=CH2cis}$ = 17.3 Hz, =CH_{2cis}), 5.16 (dd, 1H, $^2J_{=cis}$ =$^2J_{trans}$ = 1.3 Hz,$^3J_{CH=,=CH2trans}$ = 10.1 Hz, =CH_{2trans}), 4.96 (AB, 1H, ^2J = 10.7 Hz, CH_2-Ph), 4.74 (AB, 1H, ^2J = 10.7 Hz, CH_2-Ph), 4.84 (d, 1H, $^3J_{1,2}$= 1.9 Hz, H1), 4.73 (AB, 1H, ^2J = 12.3 Hz, CH_2-Ph), 4.65 (AB, 1H, ^2J = 12.1 Hz, CH_2-Ph), 4.63 (s, 2H, CH_2-Ph), 4.43 (dd, 1H, $^3J_{6,7a}$ = 3.4 Hz, $^2J_{7a,7b}$ = 12.2 Hz, H7), 4.22 (dd, 1H, $^3J_{6,7b}$ = 8.2 Hz, $^2J_{7a,7b}$ = 12.2 Hz, H7), 4.12 (m, 1H, OCH*H*, All.), 3.96- 3.91 (m, 3H, H3, H4, *H*-Allyl), 3.81 (dd, 1H, $^3J_{5-6}$= 2.1 Hz, $^3J_{4-5}$= 9.8 Hz, H-5), 3.77 (t, 1H, $^3J_{1,2}$ = $^3J_{2,3}$ = 2.4 Hz, H2), 2.04 (s, 3H, CH_3 , Ac), 2.01 (s, 3H, CH_3 , Ac). ^{13}C{1H} NMR (150.9 MHz, $CDCl_3$): 170.8 (*C*O-Ac); 170.2 (*C*O-Ac); 138.5, 138,4 and 138.3 (3x ipso C- Ph); 133.7 (*C*H-Allyl); 128.5. 128.5 128.3, 127.9 and 127.8 (C-Ph); 117.5 (*C*H_2-Allyl); 97.2 (C-1); 80.4 (C-3); 75.1 (*C*H_2-Ph); 74.97 (C-4); 74.91 (C-2), 72.8 (*C*H_2-Ph); 72.47 (*C*H_2-Ph); 72.26 (C-5); 71.17 (C-6); 68.1 (*C*H_2-Allyl); 62.9 (C-7); 20.9 and 20.8 (2x *C*H_3, Ac); HRMS (ESI) m/z calcd. for $[M+NH_4]^+$ $C_{35}H_{44}NO_9$: m/z = 622.301, found: m/z = 622,302.

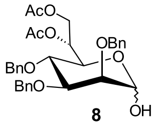

α/β = 10:1

2,3,4-tri-*O*-benzyl-6,7-di-*O*-acetyl-D-glycero-D-*manno*-heptopyranose (8)

A degassed solution of H_2-activated (1,5-cyclooctadiene)bis(methyldiphenylphosphine)-iridium(I) hexafluorophosphate (5 mg, 6 µmol) in dry THF (5 mL) was added to a degassed solution of **20** (350 mg, 0.579 mmol) in dry THF (8 mL) under atmosphere of Ar. The mixture was stirred for 2 h. at r. t., cooled to 0 °C and a solution of I_2 (323 mg, 1.273 mmol) in THF - H_2O (2:1, 15 mL) was added. The mixture was stirred for 30 min at 0 °C, diluted with EtOAc (150 mL) and washed with aq. 5% $Na_2S_2O_3$ (2 x 30 ml), sat. aq. $NaHCO_3$ (2 x 30 mL), water (30 mL) and brine (30 mL), dried over Na_2SO_4, filtered and concentrated. The residue was purified by silica gel chromatography on silica gel (toluene – EtOAc, 8 : 2 → 1 : 1) to give **8** (312 mg, 0.553 mmol, 95%) as a syrup. R_f = 0.37 (toluene - EtOAc, 8 : 2); ^{1}H NMR (600 MHz, $CDCl_3$) δ 7.31 (m, 15H,Ph), 5.55 (m, 1H, H-6), 5.20 (t, 1H, $^3J_{OH,1}$ = $^3J_{1,2}$ = 2.5 Hz, H-1), 4.96 (AB, 1H, ^2J = 10.7 Hz, CH_2-Ph), 4.75 (AB, 1H, ^2J = 10.7 Hz, CH_2-Ph), 4.74 (AB, 1H, ^2J = 12.3 Hz, CH_2-Ph) 4.64 (AB, 1H, ^2J = 12.3 Hz, CH_2-Ph), 4.63 (s, 2H, CH_2-Ph); 4.42 (dd, 1H, $^3J_{6,7a}$ = 3.3 Hz, $^2J_{7a,7b}$ = 12.2 Hz, H7), 4.18 (dd, 1H, $^3J_{6,7b}$ = 8.4 Hz, $^2J_{7a,7b}$ = 12.2 Hz, H7), 3.99-3.95 (m, 3H, H-4, H-5, H-3), 3.77 (t, 1H, $^3J_{1,2}$ = $^3J_{2,3}$ = 2.3 Hz, H2), 2.73 (s, 1H, OH), 2.05 (s, 3H, CH_3 , Ac), 2.00 (s, 3H, CH_3 , Ac). ^{13}C{1H} NMR (150.9 MHz, $CDCl_3$): 170.9 (*C*O-Ac); 170.2 (*C*O-Ac); 138.5, 138,4 and 138.3 (3x ipso C-

Ph); 128.6. 128.54 128.53, 128.3, 127.89, 127.83 and 127.80 (C-Ph); 92.8 (C-1); 79.7 (C-3); 75.12 (C-4); 75.02 (CH_2-Ph); 74.75 (C-2), 72.88 (CH_2-Ph); 72.6 (C-5); 72.4 (CH_2-Ph); 71.2 (C-6); 63.1 (C-7); 21.1 and 20.9 (2x CH_3, Ac); HRMS (ESI) m/z calcd. for $[M+NH_4]^+$ $C_{32}H_{40}NO_9$: m/z = 582.270, found: m/z = 582,270.

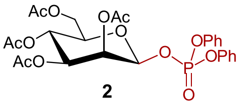

2

Diphenyl (2,3,4,6-tetra-*O*-acetyl-β-D-*manno*-pyranosyl)phosphate (2)

To a preconditioned solution of betaine [prepared from triphenylphosphine (1.436 mmol, 376mg) and diisopropyl azodicarboxylate (DIAD) (1.436 mmol, 28 µl) by stirring at r.t. for 10 min in toluene (8 ml) under atmosphere of Ar (the formation of betaine was controlled by ^{31}P-NMR)] a solution of **1**[6] (0.287 mmol, 100 mg) in toluene (1 mL) was added and the stirring was continued for 10 min. Then diphenyl phosphate (1.2 equiv., 0.344 mmol, 86 mg) was added and the reaction mixture was stirred for 3 h under atmosphere of Ar. The mixture was diluted with toluene (5 mL) and concentrated. The residue was purified by column chromatography on silica gel (toluene/EtOAc, 3:1 → 3:2) to afford **2** as a colorless syrup (115 mg, 0.198 mmol, 68%) and **3** as a colorless syrup (45 mg, 0.077 mmol, 22%). Analytical data including ^{1}H- and ^{13}C NMR were in full accordance with previously published.[7]

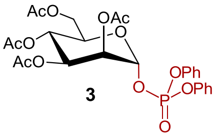

3

Diphenyl (2,3,4,6-tetra-*O*-acetyl-α-D-*manno*-pyranosyl)phosphate (3)

To a preconditioned solution of betaine [prepared from triphenylphosphine (0.640 mmol, 0.168mg) and diisopropyl azodicarboxylate (DIAD) (0.640 mmol, 13 µl) by stirring for 10 min in toluene/THF (1:1, 6 ml) under atmosphere of Ar (control by ^{31}P-NMR for the formation of betaine)] a solution of **1**[6] (0.144 mmol, 50 mg) in dry toluene (1 mL) was added under atmosphere of Ar. The stirring was continued for 10 min and diphenyl phosphate (0.284 mmol, 71 mg) and imidazole (1.281 mmol, 87 mg) were added. The reaction mixture was stirred for 1 h, diluted with toluene (5 mL) and concentrated. The residue was purified by column chromatography on silica gel (toluene/EtOAc, 3:1 → 3:2) to afford **2** as a colorless syrup (12 mg, 0.021 mmol, 15%) and **3** as a colorless syrup (62 mg, 0.107 mmol, 74%). Analytical data including ^{1}H- and ^{13}C NMR were in full accordance with previously published.[7] ^{1}H NMR (600 MHz, $CDCl_3$) δ 7.35-7.38 (m, 4H, Ph), 7.21-7.27 (m, 6H, Ph), 5.87 (dd, 1H, $^3J_{1-2}$ = 1.8 Hz, $^3J_{1-P}$ = 6.5 Hz, H-1), 5.36-5.38 (m, 1H, H-4), 5.31-5.34 (m, 2H, H-2, H-3), 4.19 (dd, 1H, $^3J_{5-6a}$ = 4.7 Hz, $^3J_{6a-6b}$ = 12.4 Hz, H-6a), 4.08 (m, 1H, H-5), 3.93 (dd, 1H, $^3J_{5-6b}$ = 2.3 Hz, $^3J_{6b-6a}$ = 12.4 Hz, H-6b), 2.16 (s, 3H, CH_3, Ac), 2.04 (s, 3H, CH_3, Ac), 2.01 (s, 3H, CH_3, Ac), 1.98 (s, 3H, CH_3, Ac).

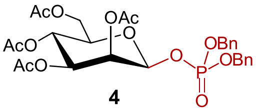

4

Dibenzyl (2,3,4,6-tetra-*O*-acetyl-β-D-*manno*-pyranosyl)phosphate (4)

To a preconditioned solution of betaine [prepared from triphenylphosphine (1.436 mmol, 376mg) and diisopropyl azodicarboxylate (1.436 mmol, 28 µl) by stirring for 10 min at r.t in toluene (8 ml) in the atmosphere of Ar (control by ^{31}P-NMR for the formation of betaine)] a solution of **1**[6] (0.287 mmol, 100 mg) in dry toluene (1 mL) was added and the stirring was continued for 15 min. Then dibenzyl phosphate (1.08 equiv., 0.309 mmol, 86 mg) was added and the stirring was continued for 3 h under atmosphere of Ar. The mixture was diluted with toluene (10 mL) and concentrated. The residue was purified by

column chromatography on silica gel (toluene/EtOAc, 3:1 → 1.5:1) to afford **4** as a colorless syrup (100 mg, 0.164 mmol, 64%) and **5** as a colorless syrup (45 mg, 0.074 mmol, 28%). Analytical data including ^{1}H- and ^{13}C NMR were in full accordance with previously published.[8] ^{1}H NMR (600 MHz, $CDCl_3$) δ 7.30-7.36 (m, 10H, Ph), 5.46 (d, 1H, $^{3}J_{2\text{-}3}$ = 3.3 Hz, H-2), 5.44 (dd, 1H, $^{3}J_{1\text{-}2}$ = 1.1 Hz, $^{3}J_{1\text{-}P}$ = 7.7 Hz, H-1), 5.24 (t, 1H, $^{3}J_{2\text{-}3}$ = $^{3}J_{3\text{-}4}$ = 9.8 Hz, H-4), 5.00-5.10 (m, 5H, H-3, 2 × CH_2 OBn), 4.24 (dd, 1H, $^{3}J_{5\text{-}6a}$ = 5.8 Hz, $^{3}J_{6a\text{-}6b}$ = 12.4 Hz, H-6a), 4.15 (dd, 1H, $^{3}J_{5\text{-}6b}$ = 2.6 Hz, $^{3}J_{6b\text{-}6a}$ = 12.3 Hz, H-6b), 3.75 (m, 1H, H-5), 2.15 (s, 3H, CH_3, Ac), 2.05 (s, 3H, CH_3, Ac), 2.00 (s, 3H, CH_3, Ac), 1.99 (s, 3H, CH_3, Ac).

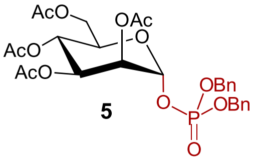

5

Dibenzyl (2,3,4,6-tetra-*O*-acetyl-α-D-*manno*-pyranosyl)phosphate (5)

To a preconditioned solution of betaine [prepared from triphenylphosphine (0.640 mmol, 0.168mg) and diisopropyl azodicarboxylate (DIAD) (0.640 mmol, 13 µl) by stirring for 10 min at r.t. in toluene/THF (1:1, 6 ml) under atmosphere of Ar] a solution of **1**[6] (0.115 mmol, 40 mg) in dry toluene (1 mL) was added. The stirring was continued for 10 min under atmosphere of Ar and dibenzyl phosphate (0.137 mmol, 38 mg) and imidazole (1,148 mmol, 78 mg) were added. The reaction mixture was stirred for 1 h under atmosphere of Ar, diluted with toluene (5 mL) and concentrated. The residue was purified by column chromatography on silica gel (toluene/EtOAc, 3:1 → 1.5:1) to afford **4** as a colorless syrup (9 mg, 0.014 mmol, 12%) and **5** as a colorless syrup (48 mg, 0.079 mmol, 69%). Analytical data including ^{1}H- and ^{13}C NMR were in full accordance with previously published.[8] ^{1}H NMR (600 MHz, $CDCl_3$) δ 7.34-7.37 (m, 10H, Ph), 5.61 (dd, 1H, $^{3}J_{1\text{-}2}$ = 1.9 Hz, $^{3}J_{1\text{-}P}$ = 6.4 Hz, H-1), 5.28-5.29 (m, 2H, H-3, H-4), 5.23 (m, 1H, H-2), 5.07-5.11 (m, 4H, 2 × CH_2 OBn), 4.18 (dd, 1H, $^{3}J_{5\text{-}6a}$ = 4.7 Hz, $^{3}J_{6a\text{-}6b}$ = 12.5 Hz, H-6a), 4.05 (m, 1H, H-5), 3.94 (dd, 1H, $^{3}J_{5\text{-}6b}$ = 2.3 Hz, $^{3}J_{6b\text{-}6a}$ = 12.5 Hz, H-6b), 2.15 (s, 3H, CH_3, Ac), 2.03 (s, 3H, CH_3, Ac), 2.00 (s, 3H, CH_3, Ac), 1.99 (s, 3H, CH_3, Ac).

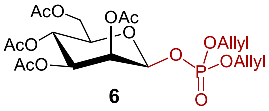

6

Diallyl (2,3,4,6-tetra-*O*-acetyl-β-D-*manno*-pyranosyl)phosphate (6)

To a preconditioned solution of betaine [prepared from triphenylphosphine (1.436 mmol, 376mg) and diisopropyl azodicarboxylate (1.436 mmol, 28 µl) by stirring for 10 min at r.t. in toluene (8 ml) under atmosphere of Ar (the formation of betaine was controlled by ^{31}P-NMR)] a solution of **1**[6] (0.287 mmol, 100 mg) in toluene (1 mL) was added. The stirring was continued for 15 min under atmosphere of Ar and a solution of diallyl phosphate (1.18 equiv., 0.345 mmol, 61 mg) in toluene (1 mL) was added. The reaction mixture was stirred for 3 h at r.t., diluted with toluene (5 mL) and concentrated. The residue was purified by column chromatography on silica gel (toluene/EtOAc, 3:1 → 1:1) to afford **6** as a colorless syrup (89 mg, 0.175 mmol, 61%) and **7** as a colorless syrup (31 mg, 0.061 mmol, 20%). Analytical data including ^{1}H- and ^{13}C NMR were in full accordance with previously published.[9] ^{1}H NMR (600 MHz, $CDCl_3$) δ 5.92 (m, 2H, 2 × C*H*-Allyl), 5.52 (d, 1H, $^{3}J_{2\text{-}3}$ = 3.2 Hz, H-2), 5.48 (dd, 1H, $^{3}J_{1\text{-}2}$ = 1.1 Hz, $^{3}J_{1\text{-}P}$ = 7.8 Hz, H-1), 5.37 (dd, 1H, $^{2}J_{=CH2cis,=CH2trans}$ = 1.5 Hz, $^{3}J_{CH=,=CH2cis}$ = 16.8 Hz, =CH_{2cis}), 5.35 (dd, 1H, $^{2}J_{=CH2cis,=CH2trans}$ = 1.5 Hz, $^{3}J_{CH=,=CH2trans}$ = 16.8 Hz, =CH_{2trans}), 5.22-5.27 (m, 3H, =CH_{2cis}, H-4, =CH_{2trans}), 5.09 (dd, 1H, $^{3}J_{2\text{-}3}$ = 3.3 Hz, $^{3}J_{3\text{-}4}$ = 9.9 Hz, H-3), 4.55 (m, 4H, 2 × OCH*H*, All.), 4.27 (dd, 1H, $^{3}J_{5\text{-}6a}$ = 5.9 Hz, $^{3}J_{6a\text{-}6b}$ = 12.3 Hz, H-6a), 4.19 (dd, 1H, $^{3}J_{5\text{-}6b}$ = 2.6 Hz, $^{3}J_{6b\text{-}6a}$ = 12.3 Hz, H-6b), 3.79 (m, 1H, H-5), 2.19 (s, 3H, CH_3, Ac), 2.07 (s, 3H, CH_3, Ac), 2.06 (s, 3H, CH_3, Ac), 2.00 (s, 3H, CH_3, Ac).

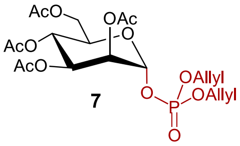

7

Diallyl (2,3,4,6-tetra-*O*-acetyl-β-D-*manno*-pyranosyl)phosphate (7)

To a preconditioned solution of betaine [prepared from triphenylphosphine (0.574 mmol, 0.150 mg) and diisopropyl azodicarboxylate (DIAD) (0.574 mmol, 11 µl) by stirring for 10 min at r.t. in toluene/THF (1:1, 6 ml) under atmosphere of Ar] a solution of **1**[6] (0.115 mmol, 40 mg) in dry toluene (1 mL) was added. The reaction mixture was stirred for 15 min under atmosphere of Ar and a solution of diallyl phosphate (1.17 equiv., 0.133 mmol, 24 mg) in toluene (1 mL) and imidazole (1.281 mmol, 87 mg) were added under stirring. The reaction mixture was stirred for 1 h, diluted with toluene (5 mL) and concentrated. The residue was purified by column chromatography on silica gel (toluene/EtOAc, 3:1 → 1.5:1) to afford **6** as a colorless syrup (9 mg, 0.009 mmol, 15%) and **7** as a colorless syrup (41 mg, 0.081 mmol, 70%). Analytical data including ^{1}H- and ^{13}C NMR were in full accordance with previously published.[9] ^{1}H NMR (600 MHz, $CDCl_3$) δ 5.96 (m, 2H, 2 × C*H*-Allyl), 5.65 (dd, 1H, $^3J_{1\text{-}2}$ = 1.8 Hz, $^3J_{1\text{-}P}$ = 6.6 Hz, H-1), 5.41 (dd, 1H, $^2J_{=CH2cis,=CH2trans}$ = 1.5 Hz, $^3J_{CH=,=CH2cis}$ = 17.1 Hz, =CH_{2cis}), 5.39 (dd, 1H, $^2J_{=CH2cis,=CH2trans}$ = 1.5 Hz, $^3J_{CH=,=CH2trans}$ = 17.1 Hz, =CH_{2trans}), 5.28-5.37 (m, 5H, H-2, H-4, H-3, =CH_{2cis}, =CH_{2trans}), 4.61 (m, 4H, 2 × OCH*H*, All.), 4.29 (dd, 1H, $^3J_{5\text{-}6a}$ = 4.9 Hz, $^3J_{6a\text{-}6b}$ = 12.4 Hz, H-6a), 4.20 (m, 1H, H-5), 4.12 (dd, 1H, $^3J_{5\text{-}6b}$ = 2.3 Hz, $^3J_{6b\text{-}6a}$ = 12.4 Hz, H-6b), 2.17 (s, 3H, CH_3, Ac), 2.09 (s, 3H, CH_3, Ac), 2.05 (s, 3H, CH_3, Ac), 2.00 (s, 3H, CH_3, Ac).

References

(1) (a) Camp, D.; von Itzstein, M.; Jenkins, I. D. The mechanism of the first step of the Mitsunobu reaction. *Tetrahedron* **2015**, *71* (30), 4946-4948. (b) Morrison, D. Notes: Reactions of Alkyl Phosphites with Diethyl Azodicarboxylate. *J. Org. Chem.* **1958**, *23* (7), 1072-1074. DOI: 10.1021/jo01101a619. (c) Crich, D.; Dyker, H.; Harris, R. J. Some observations on the mechanism of the Mitsunobu reaction. *J. Org. Chem.* **1989**, *54* (1), 257-259. DOI: 10.1021/jo00262a057.
(2) (a) Smith, A. B.; Hale, K. J.; Rivero, R. A. An efficient synthesis of glycosyl esters exploiting the mitsunobu reaction. *Tetrahedron Lett.* **1986**, *27* (48), 5813-5816. DOI: https://doi.org/10.1016/S0040-4039(00)85334-4. (b) Loibner, H.; Zbiral, E. Reaktionen mit phosphororganischen Verbindungen. XLII. Nucleophile Substitutionen an Hydroxysteroiden mit Hilfe von Triphenylphosphan/Azodicarbonsäureester. *Helv. Chim. Acta* **1977**, *60* (2), 417-425. DOI: https://doi.org/10.1002/hlca.19770600213.
(3) Itzstein, M. v.; Mocerino, M. (p-Dimethylaminophenyl) Diphenylphosphine: A More Practical Phosphine in the Mitsunobu Reaction. *Synth. Commun.* **1990**, *20* (13), 2049-2057. DOI: 10.1080/00397919008053136.
(4) Walker, T. E.; London, R. E.; Whaley, T. W.; Barker, R.; Matwiyoff, N. A. Carbon-13 nuclear magnetic resonance spectroscopy of [1-13C] enriched monosaccharides. Signal assignments and orientational dependence of geminal and vicinal carbon-carbon and carbon-hydrogen spin-spin coupling constants. *J. Am. Chem. Soc* **1976**, *98* (19), 5807-5813. DOI: 10.1021/ja00435a011.
(5) Inuki, S.; Aiba, T.; Kawakami, S.; Akiyama, T.; Inoue, J. i.; Fujimoto, Y. Chemical Synthesis of D-glycero-D-manno-Heptose 1,7-Bisphosphate and Evaluation of Its Ability to Modulate NF-κB Activation. *Org. Lett* **2017**, *19* (12), 3079-3082. DOI: 10.1021/acs.orglett.7b01158.
(6) Mikamo, M. Facile 1-O-deacylation of per-O-acylaldoses. *Carbohydr. Res.* **1989**, *191* (1), 150-153. DOI: https://doi.org/10.1016/0008-6215(89)85056-6.
(7) van Summeren, R. P.; Moody, D. B.; Feringa, B. L.; Minnaard, A. J. Total Synthesis of Enantiopure β-D-Mannosyl Phosphomycoketides from Mycobacterium tuberculosis. *J. Am. Chem. Soc.* **2006**, *128* (14), 4546-4547. DOI: 10.1021/ja060499i.
(8) Sim, M. M.; Kondo, H.; Wong, C. H. Synthesis of dibenzyl glycosyl phosphites using dibenzyl N,N-diethylphosphoramidite as phosphitylating reagent: an effective route to glycosyl phosphates, nucleotides, and glycosides. *J. Am. Chem. Soc* **1993**, *115* (6), 2260-2267.
(9) Li, T.; Tikad, A.; Pan, W.; Vincent, S. P. β-Stereoselective Phosphorylations Applied to the Synthesis of ADP- and Polyprenyl-β-Mannopyranosides. *Org. Lett.* **2014**, *16* (21), 5628-5631.

^{1}H NMR (600 MHz, $CDCl_3$)

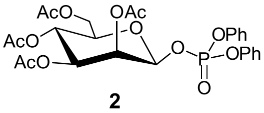

2

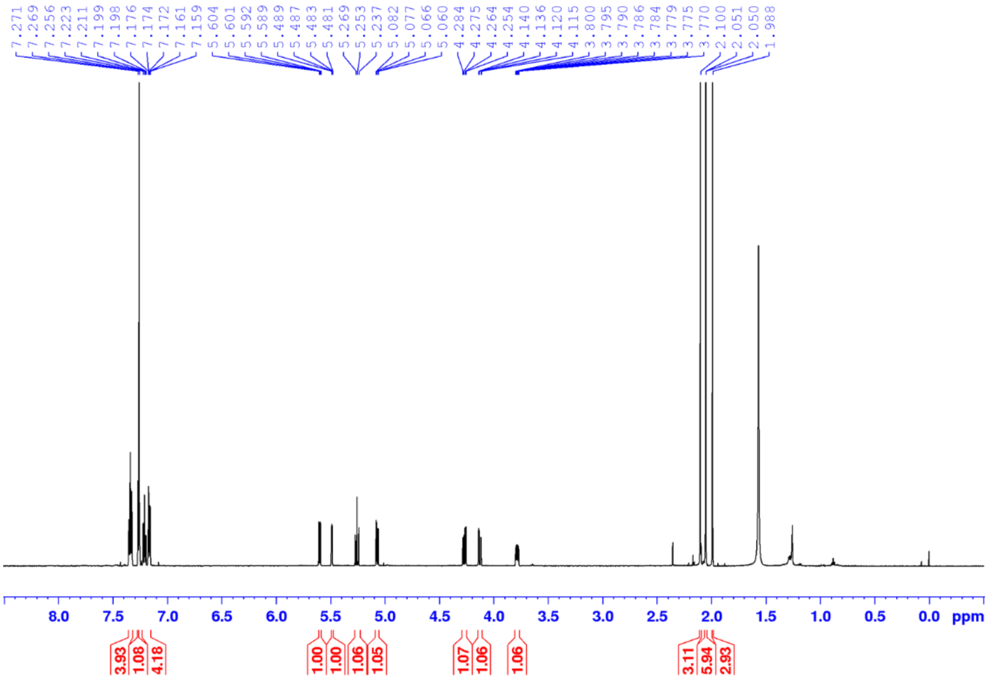

^{1}H NMR (600 MHz, $CDCl_3$)

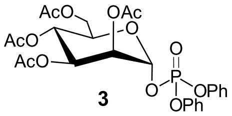

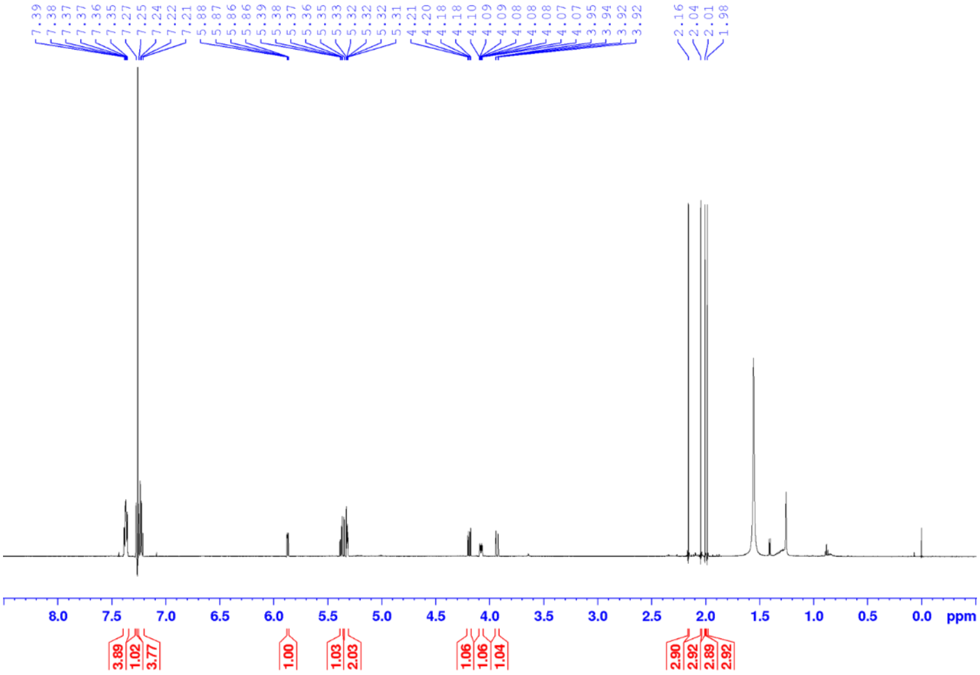

^{1}H NMR (600 MHz, $CDCl_3$)

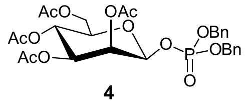

4

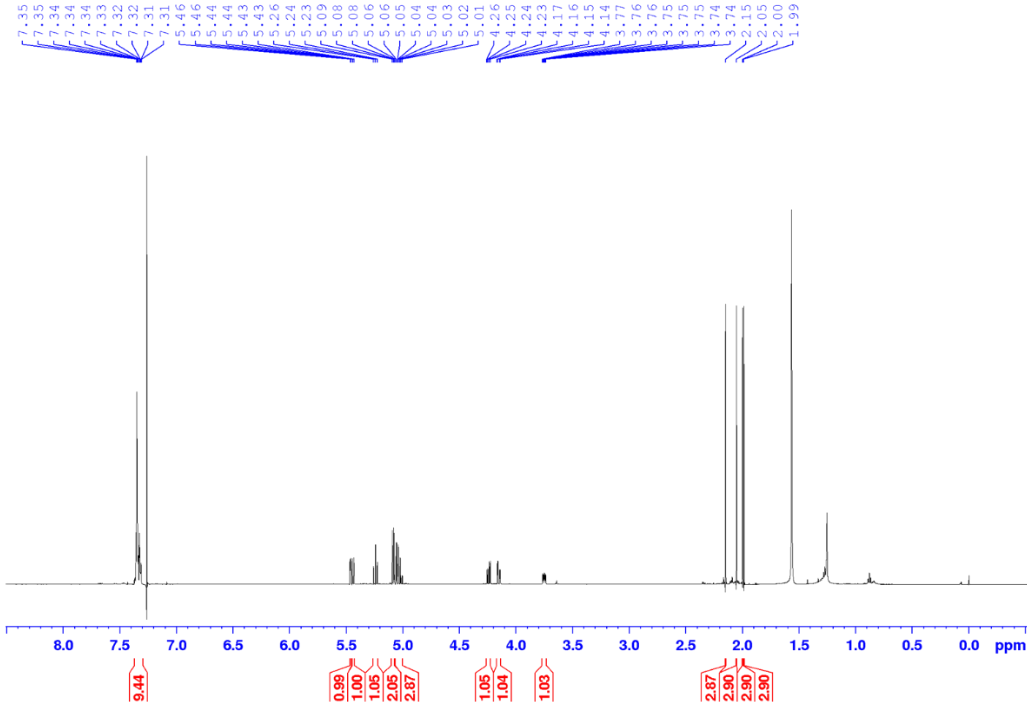

^{1}H NMR (600 MHz, $CDCl_3$)

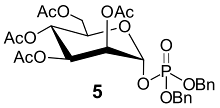

5

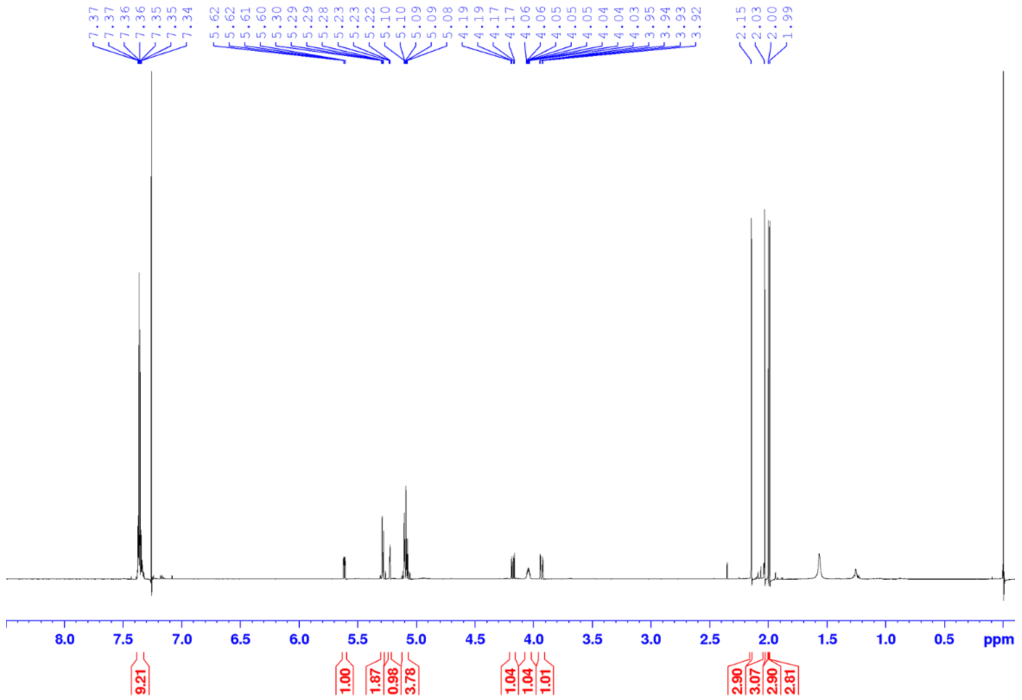

^{1}H NMR (600 MHz, $CDCl_3$)

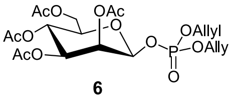

6

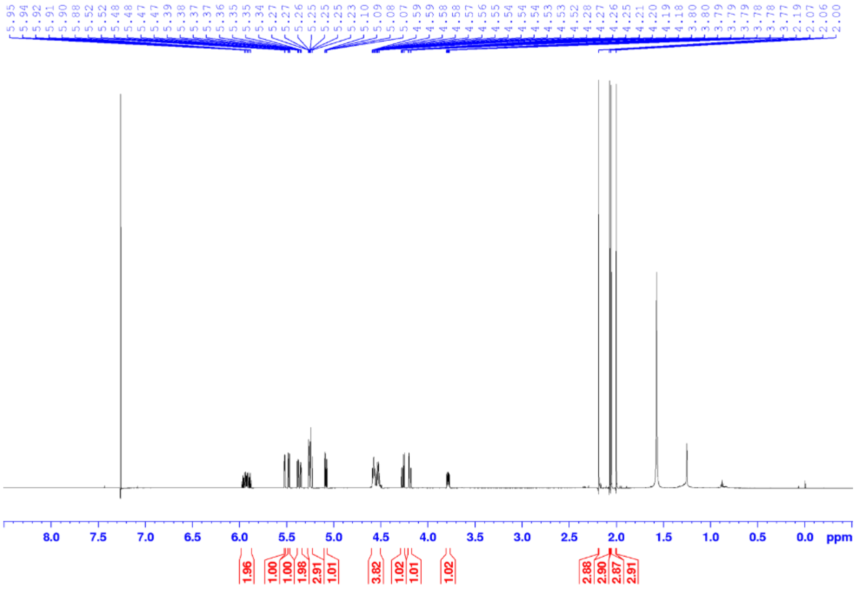

^{1}H NMR (600 MHz, $CDCl_3$)

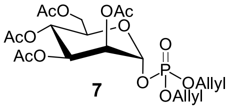

7

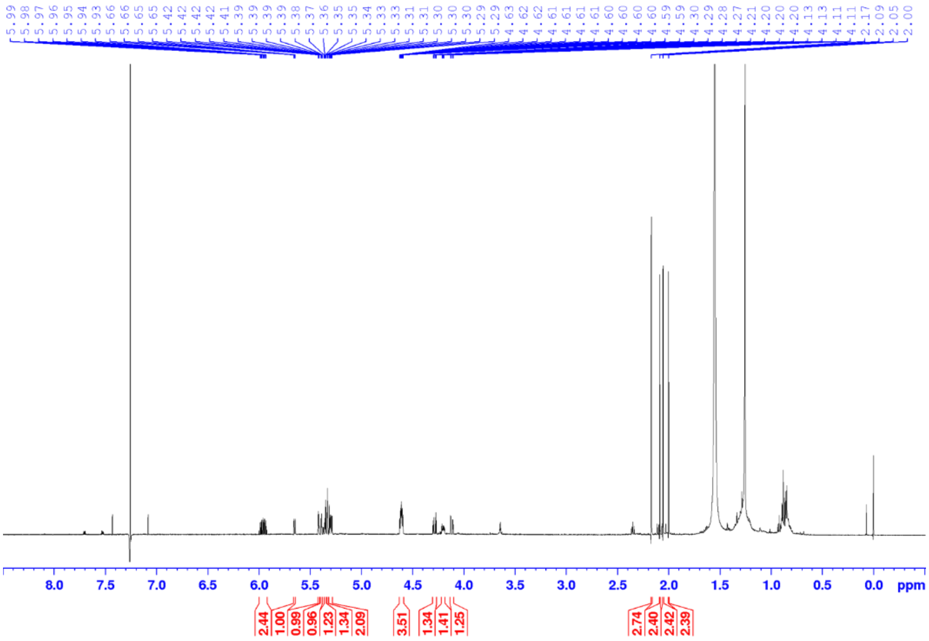

^{1}H NMR (600 MHz, $CDCl_3$)

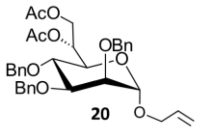

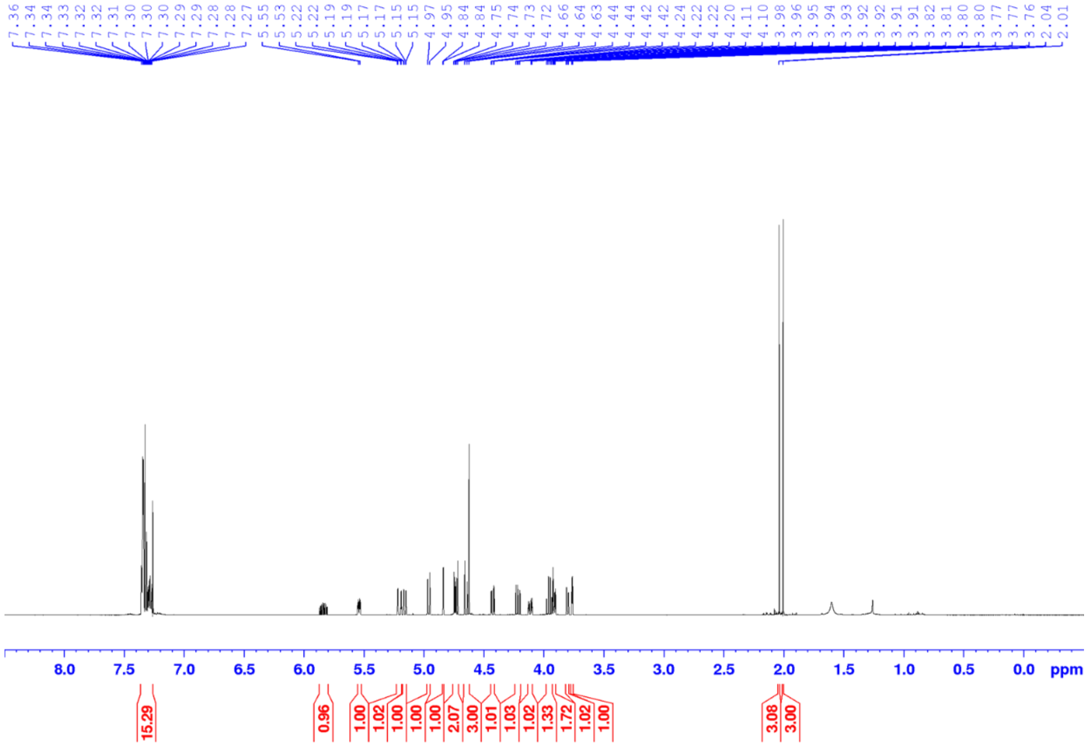

^{13}C{1H} NMR (151 MHz, $CDCl_3$)

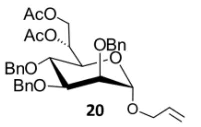

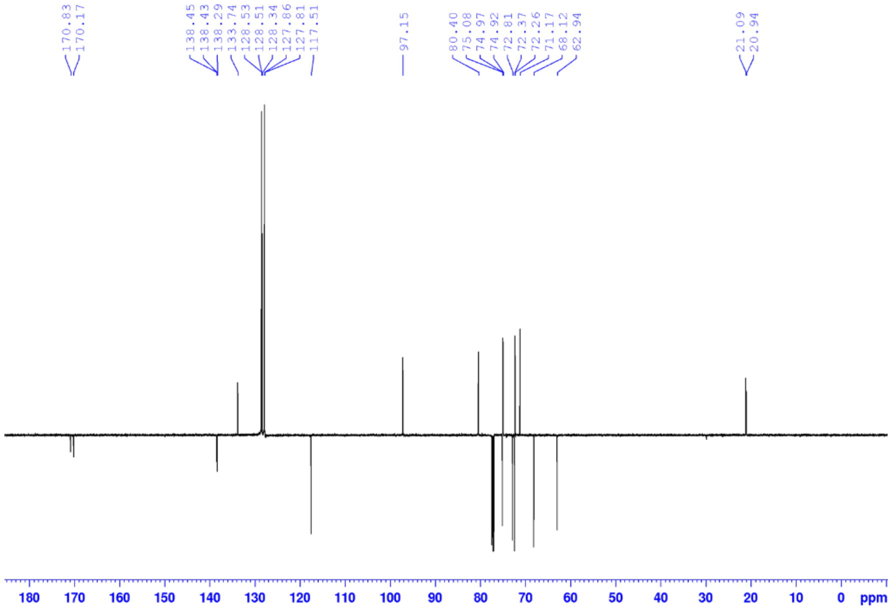

8, ^{1}H NMR (600 MHz, $CDCl_3$)

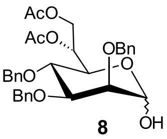

8

α/β = 10:1

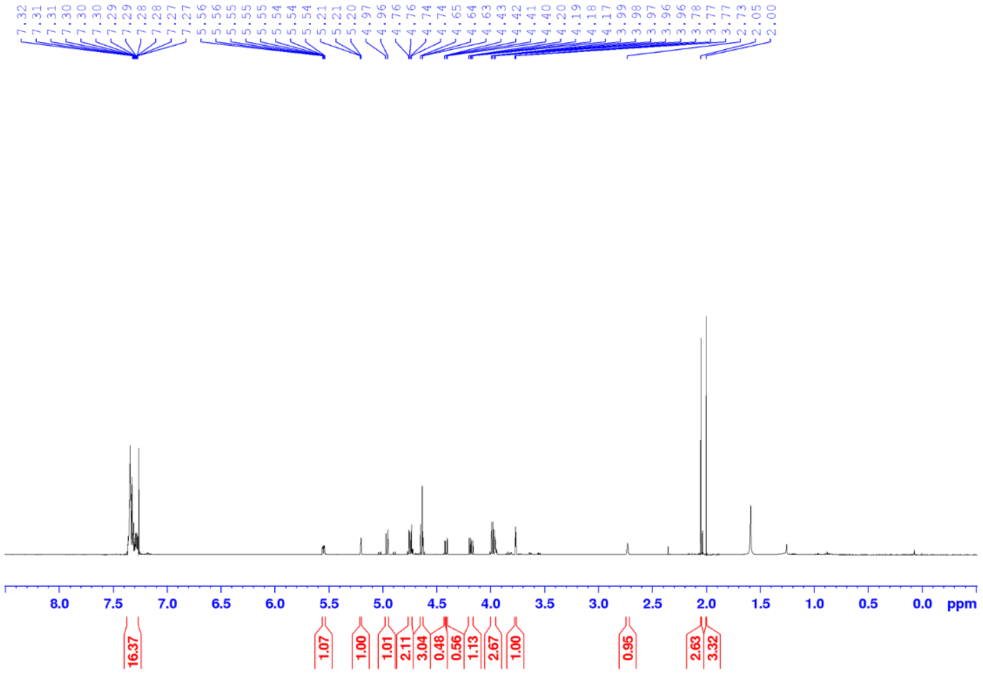

8, ^{13}C{1H} NMR (151 MHz, $CDCl_3$)

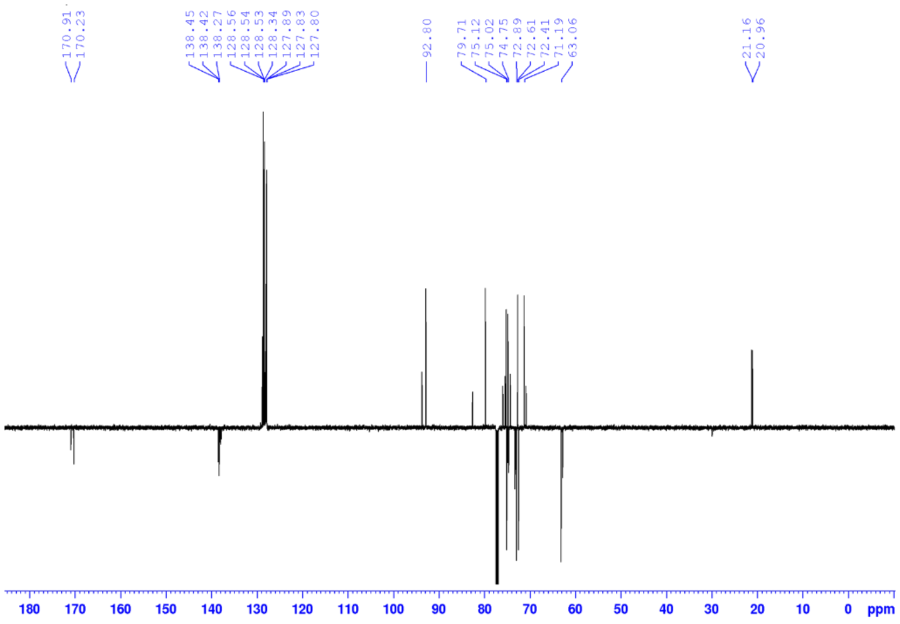

9, ^{1}H NMR (600 MHz, CDCl$_3$)

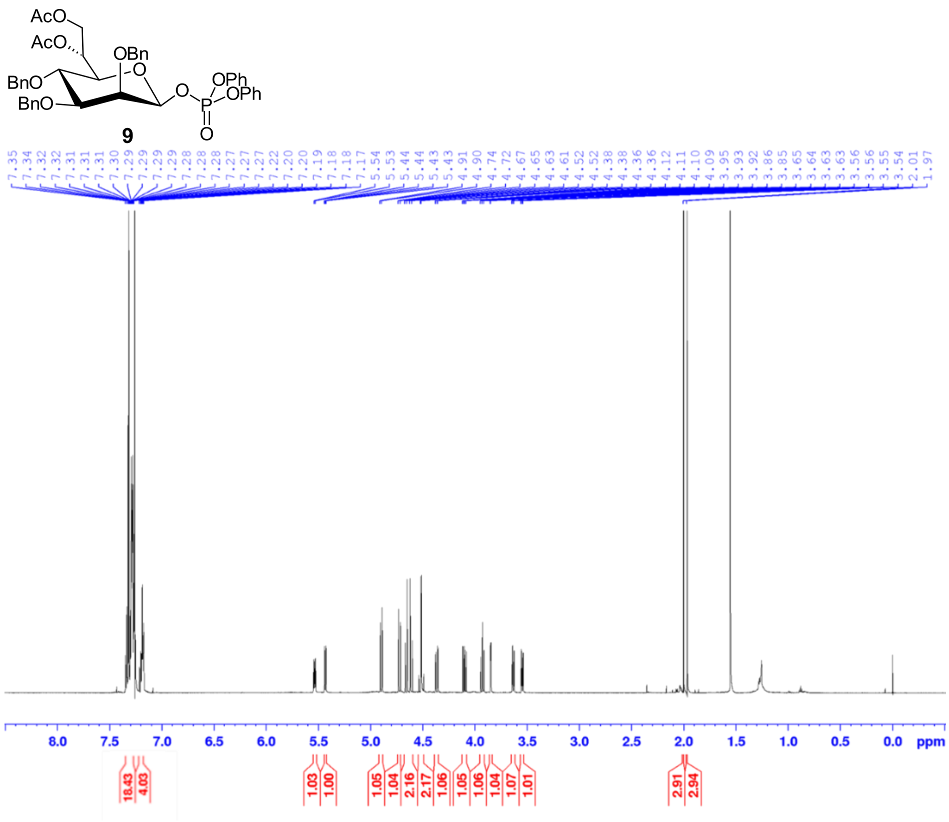

9, ^{13}C{1H} NMR (151 MHz, CDCl$_3$)

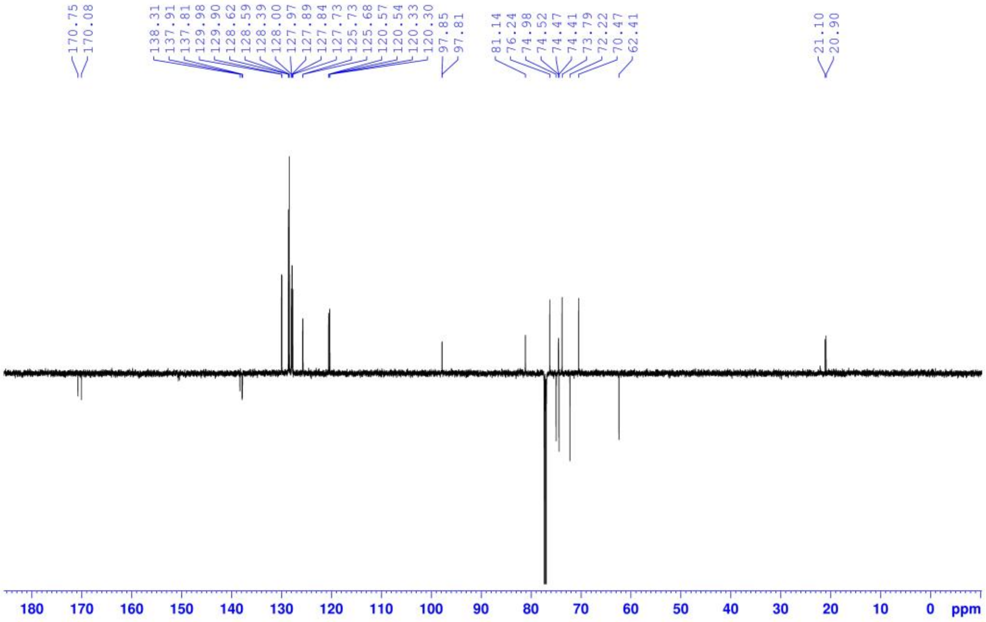

10, ^{1}H NMR (600 MHz, $CDCl_3$)

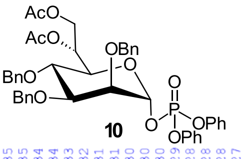

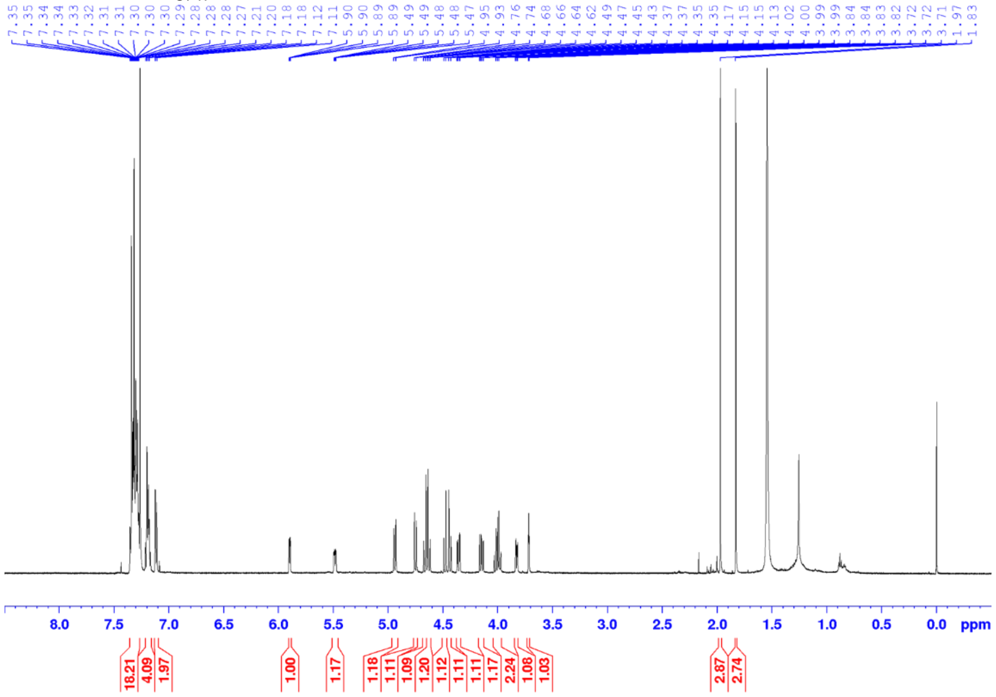

10, ^{13}C{1H} NMR (151 MHz, $CDCl_3$)

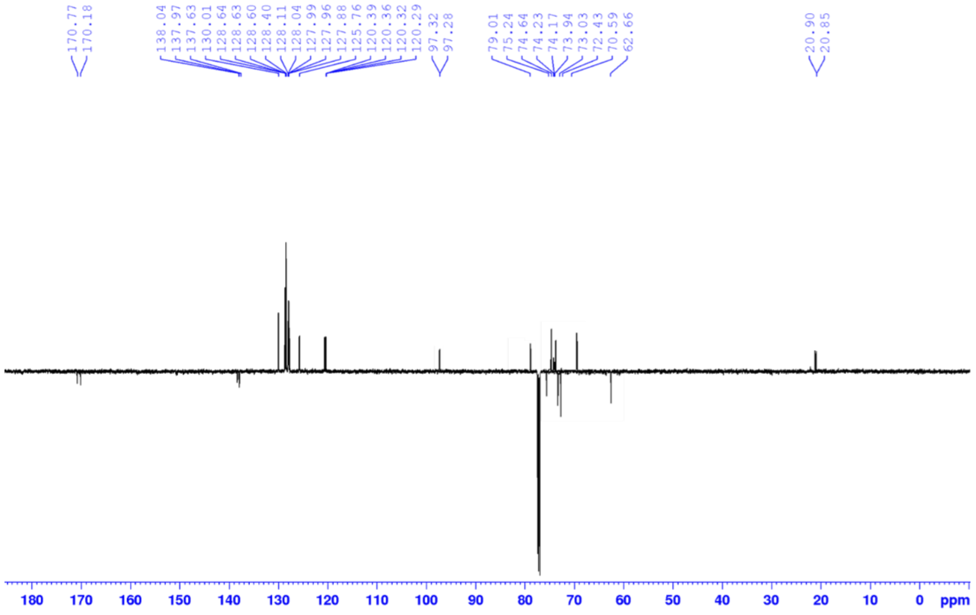

11, ^{1}H NMR (600 MHz, CDCl$_3$)

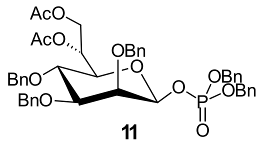

11

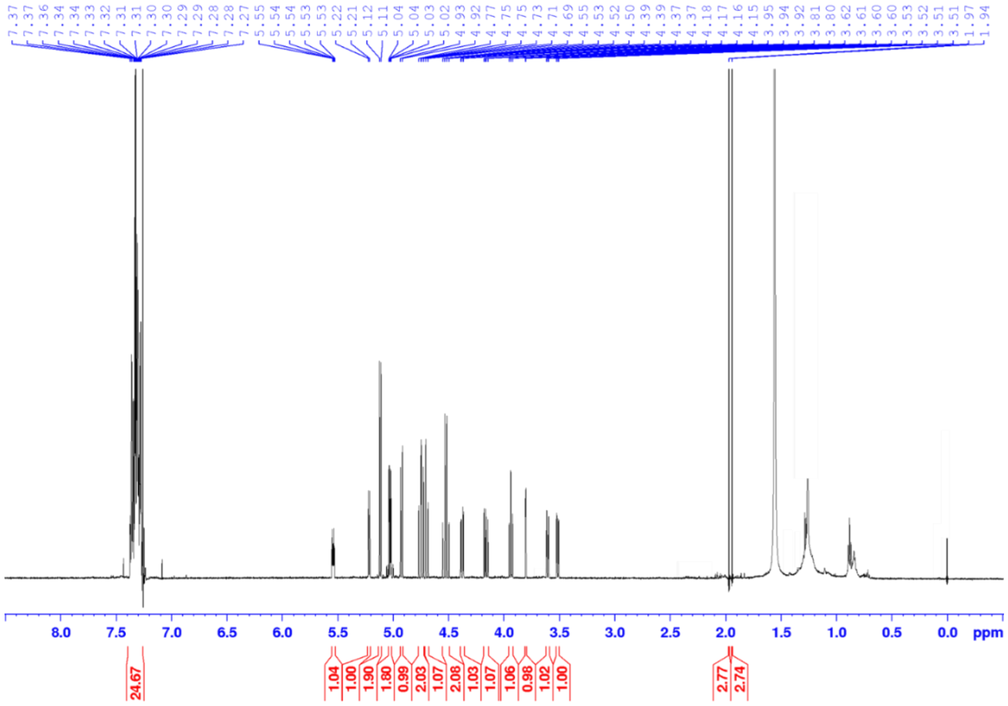

11, ^{13}C{1H} NMR (151 MHz, CDCl$_3$)

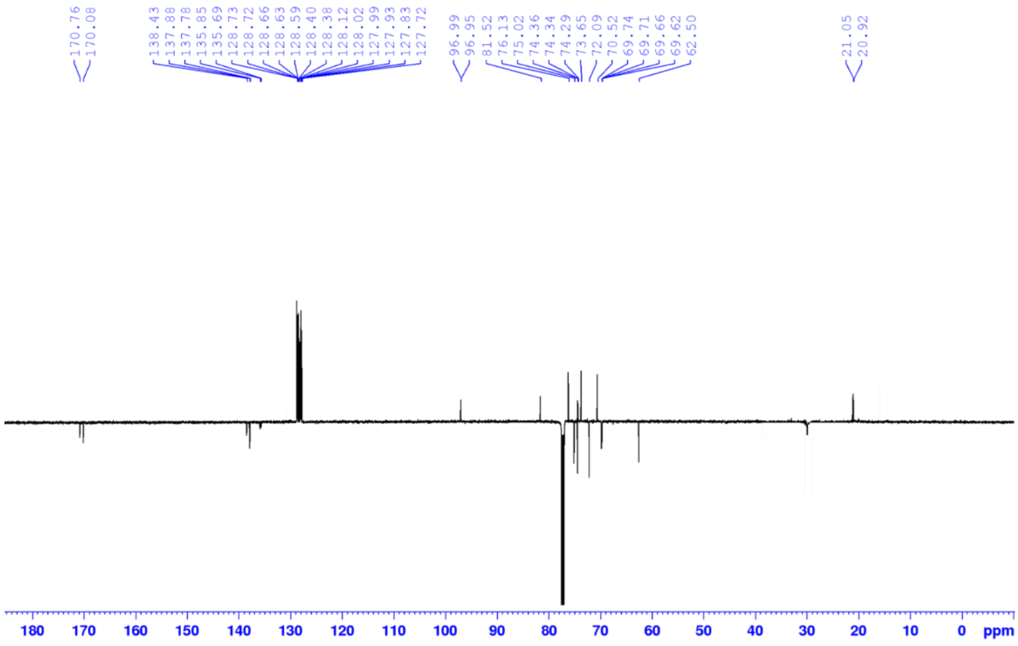

12, ^{1}H NMR (600 MHz, CDCl$_3$)

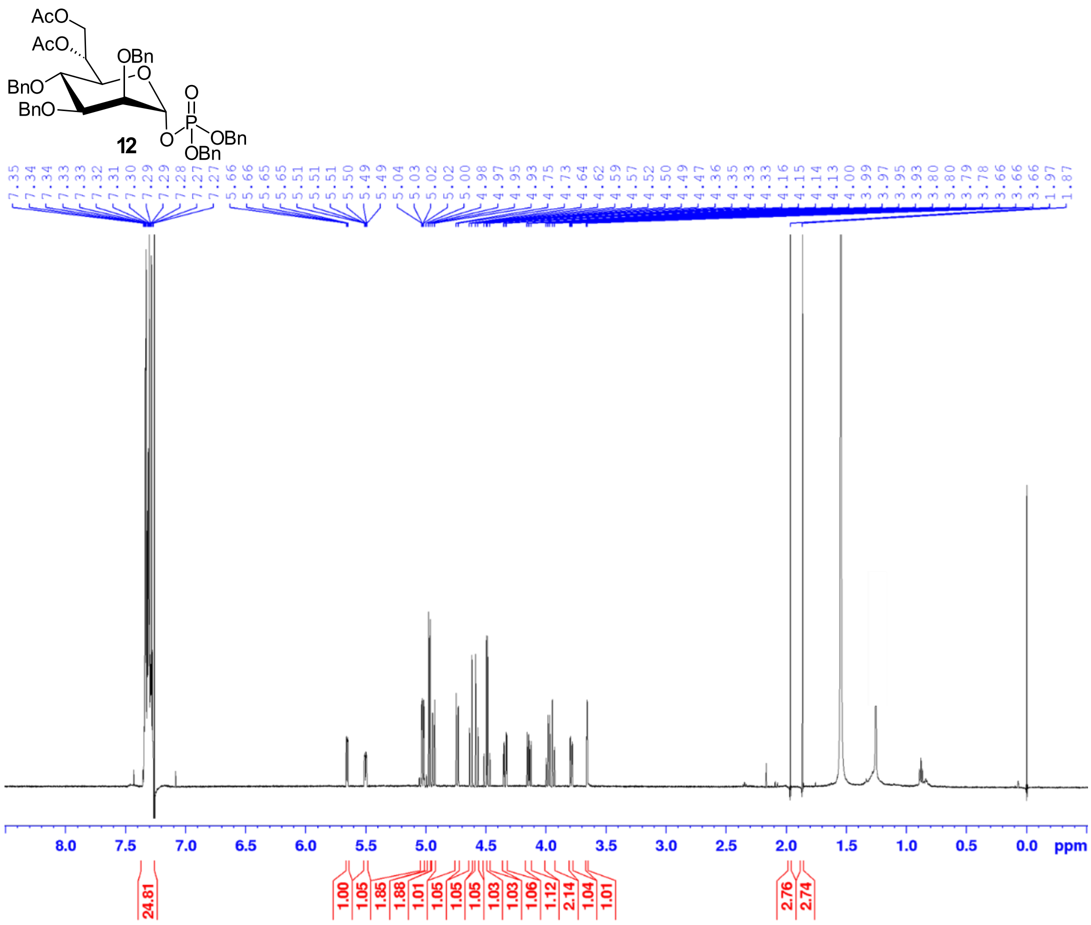

12, ^{13}C{1H} NMR (151 MHz, CDCl$_3$)

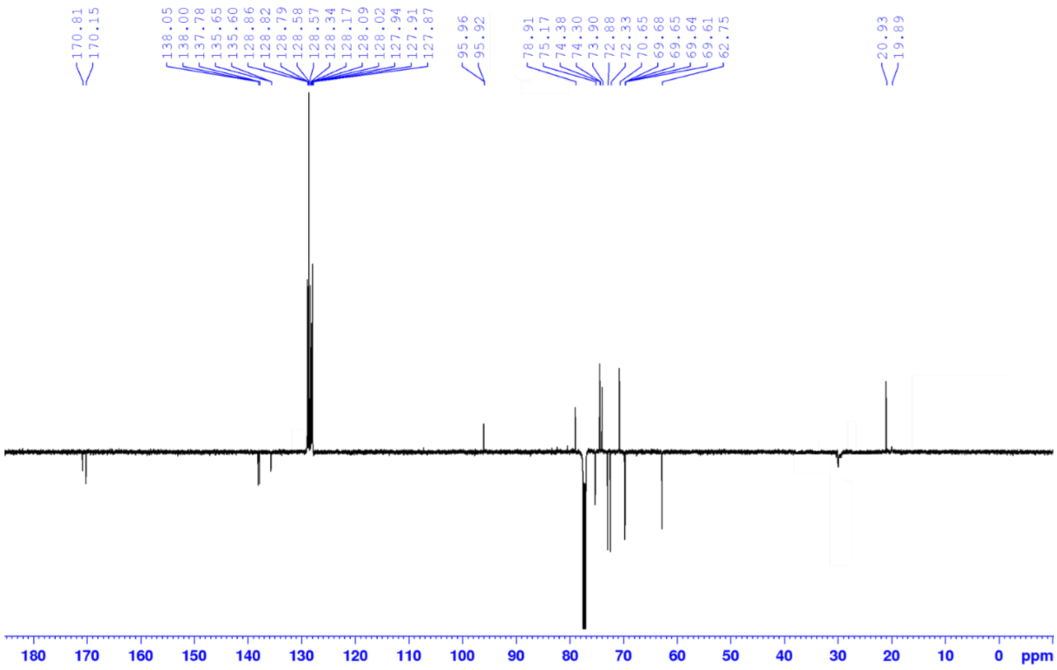

13, ^{1}H NMR (600 MHz, CDCl$_3$)

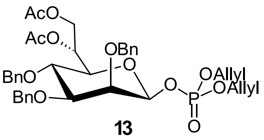

13

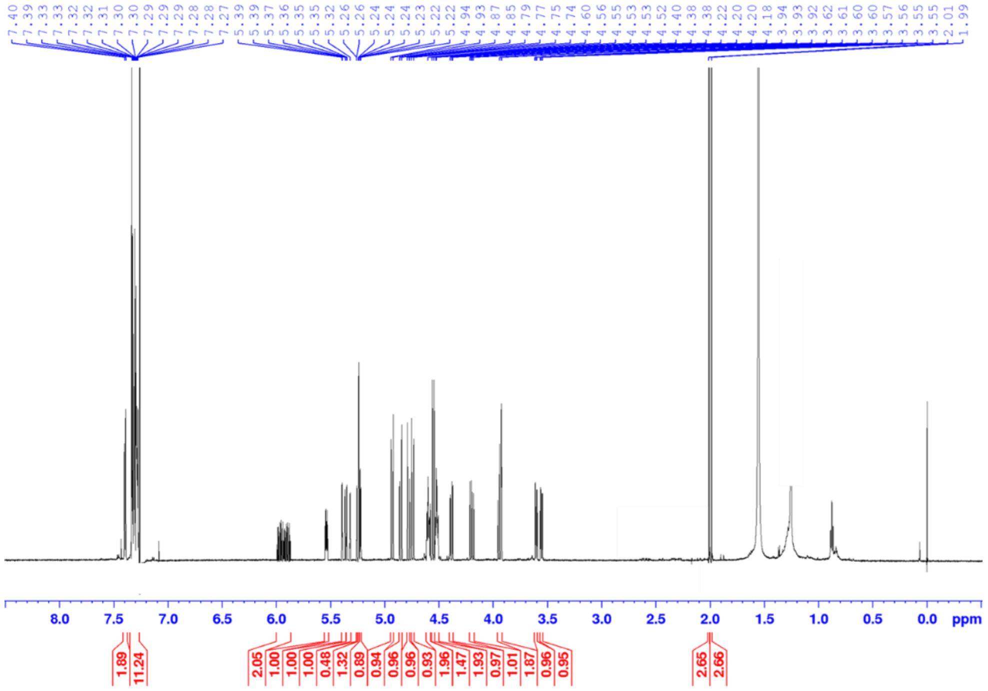

13, ^{13}C{1H} NMR (151 MHz, CDCl$_3$)

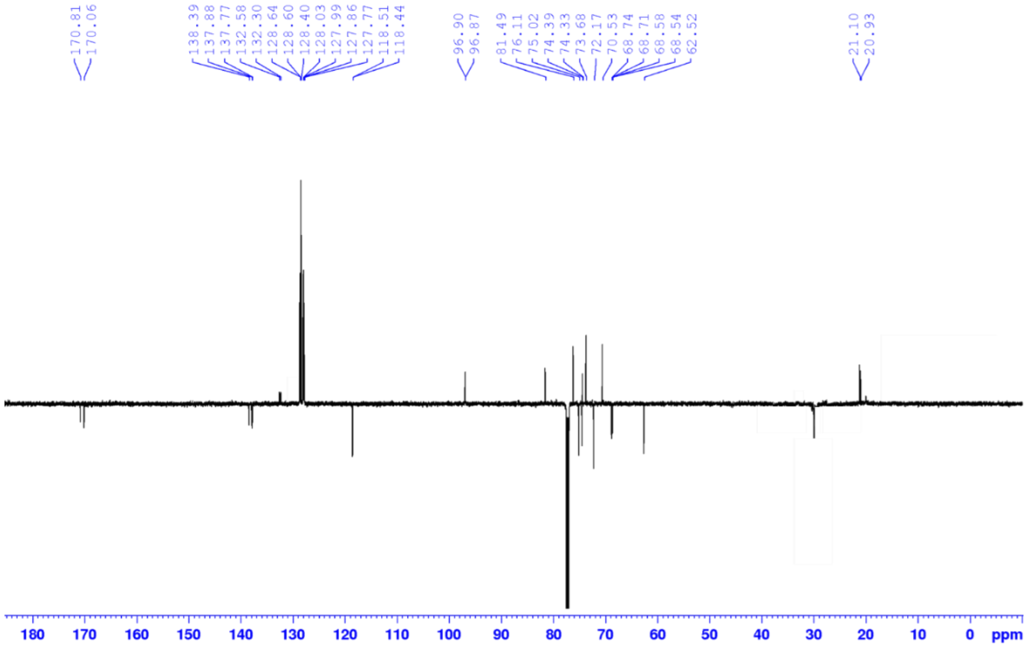

14, ^{1}H NMR (600 MHz, $CDCl_3$)

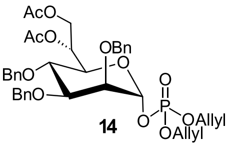

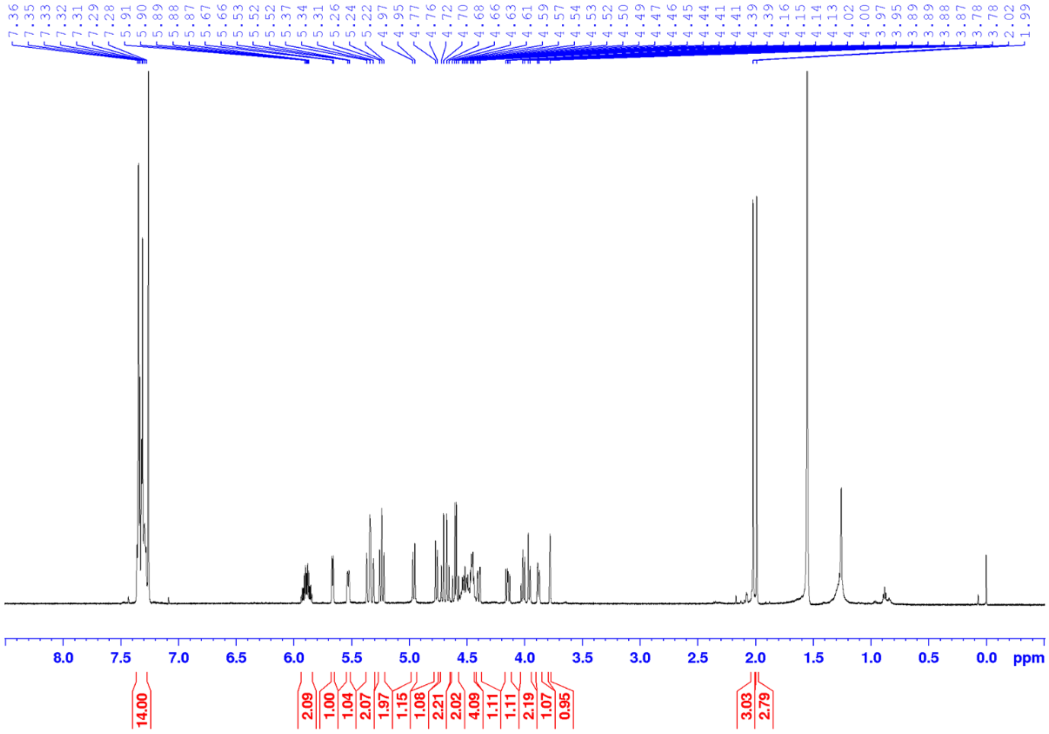

14, ^{13}C{1H} NMR (151 MHz, $CDCl_3$)

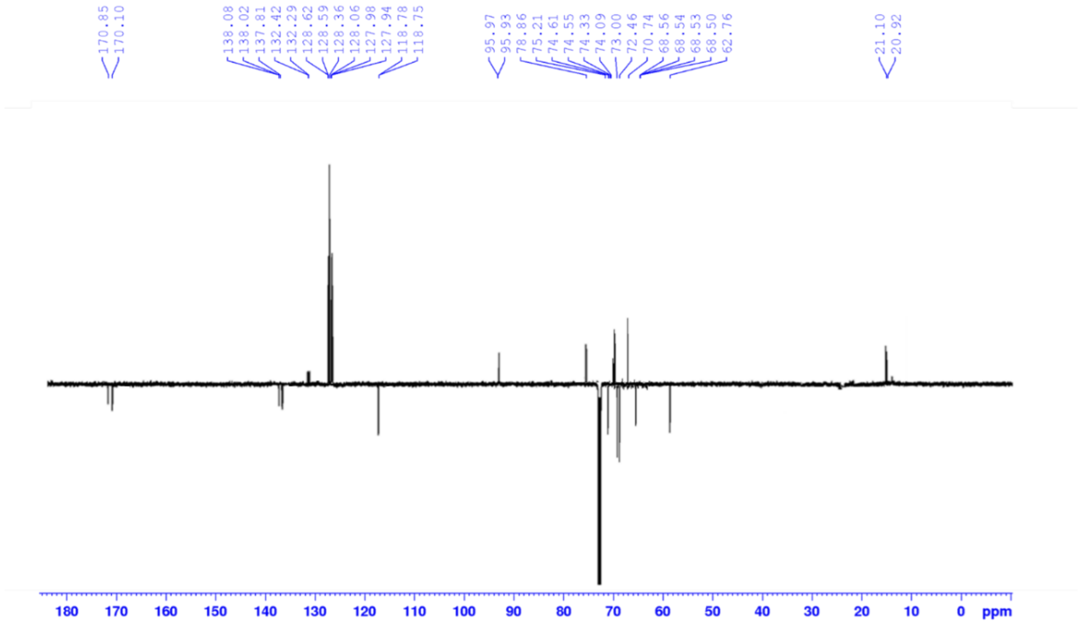

17, ^{1}H NMR (600 MHz, toluene-d$_8$), reaction performed in the NMR tube, Figure S1.

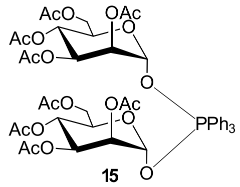

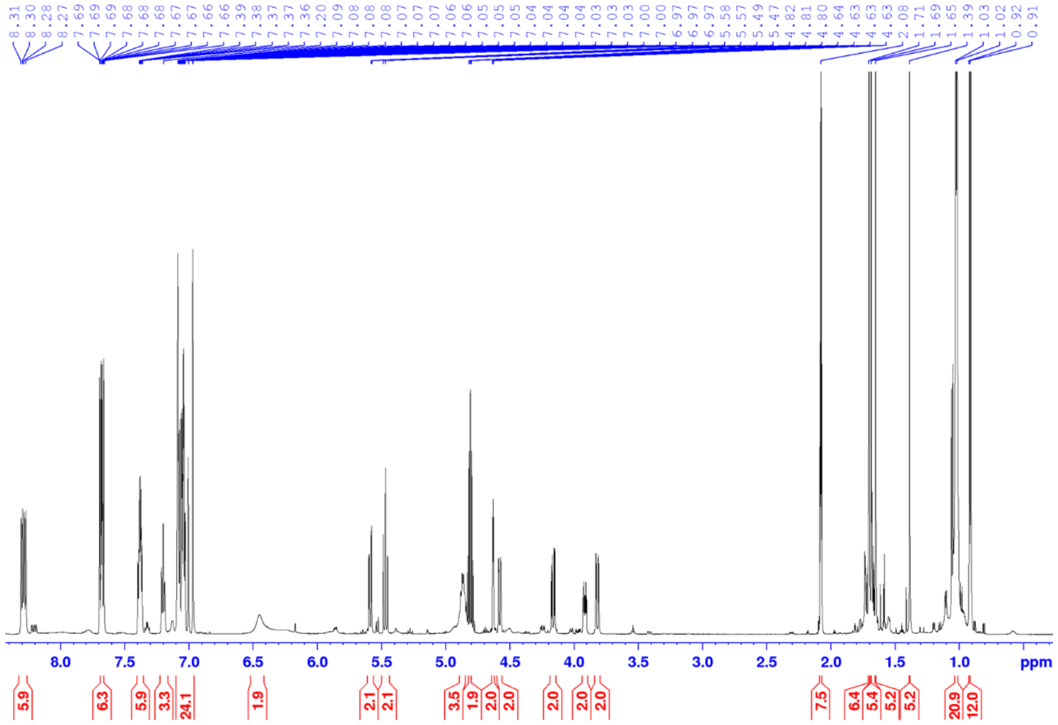

17, $^{13}C\{1H\}$ NMR (151 MHz, Toluene-d$_8$)

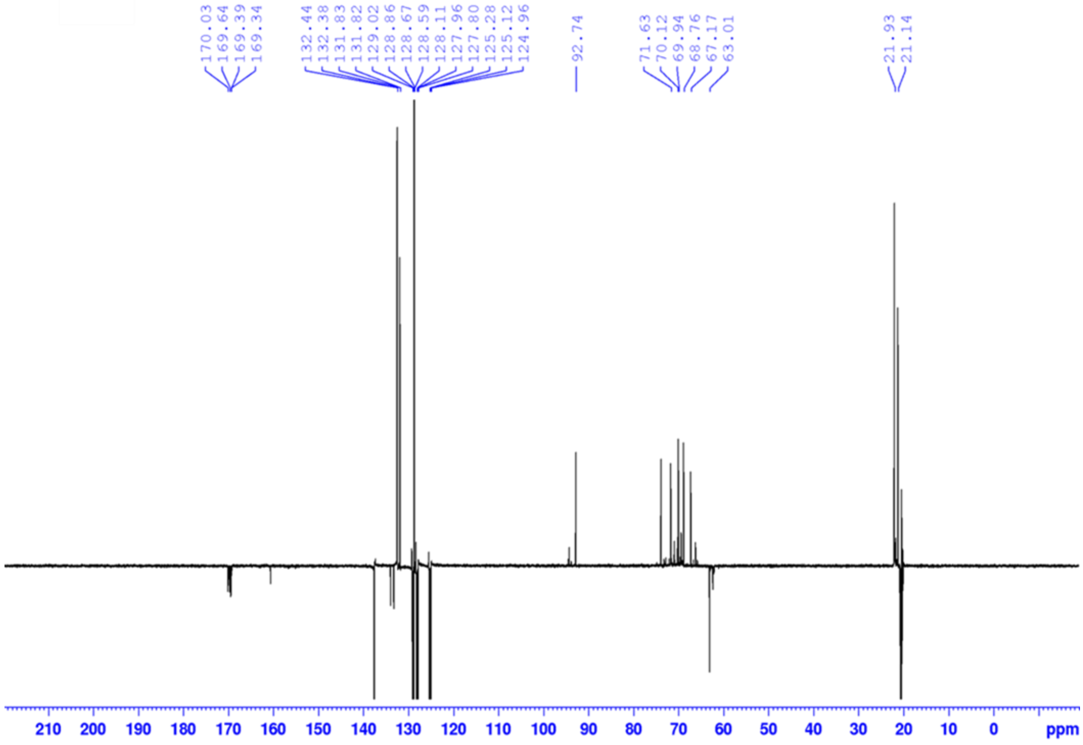

17, ^{1}H-^{31}P HMBC (600/243 MHz, Toluene-d8)

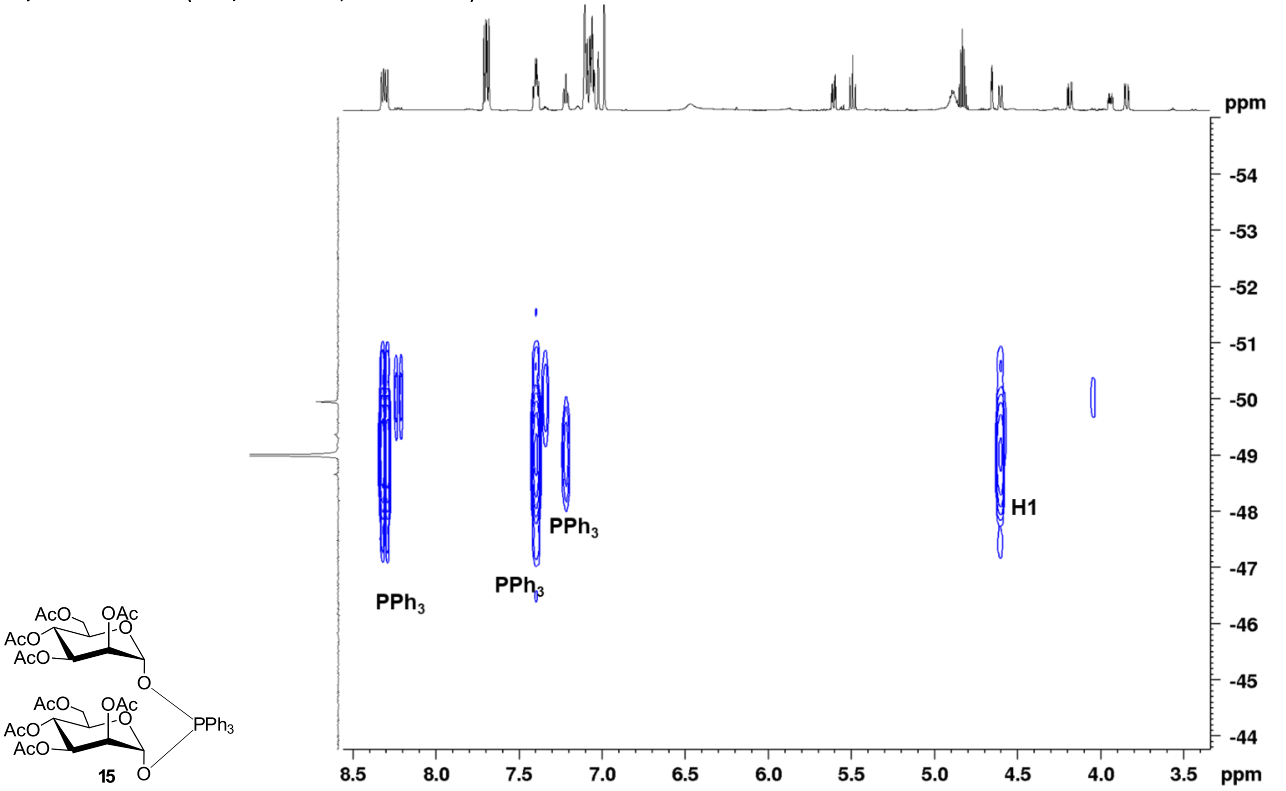

17, ^{1}H-^{13}C HSQC (600/151 MHz, Toluene-d8)

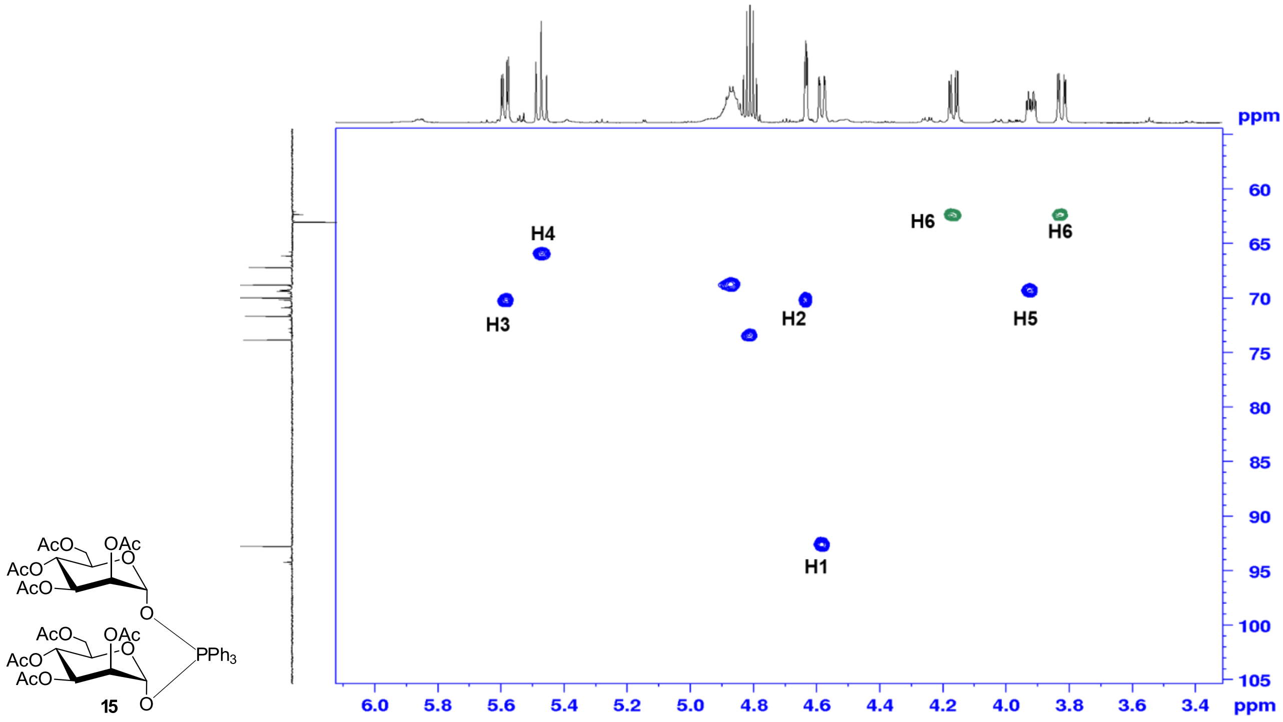

18, ^{1}H NMR (600 MHz, toluene-d_8), reaction performed in the NMR tube, Figure S3.

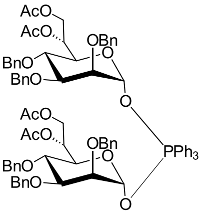

18

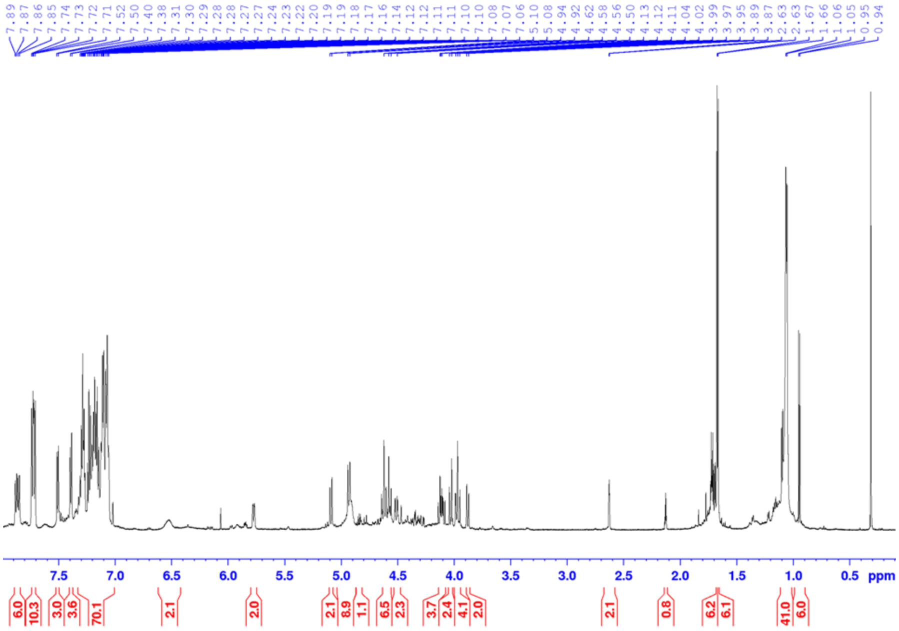

18, ^{1}H-^{31}P HMBC (600/243 MHz, Toluene-d_8)

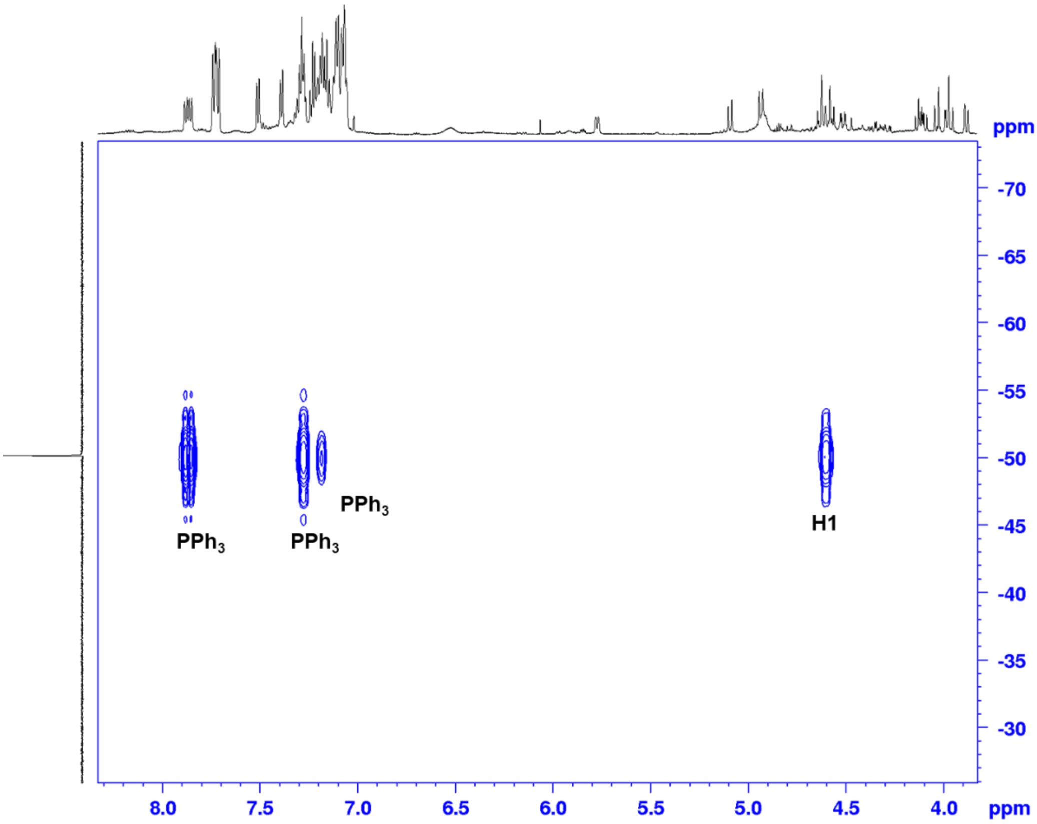

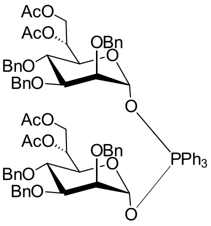

18

18, ^{1}H-^{13}C HSQC (600/151 MHz, Toluene-d_8)

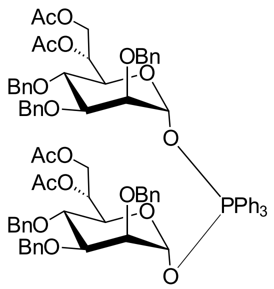

18

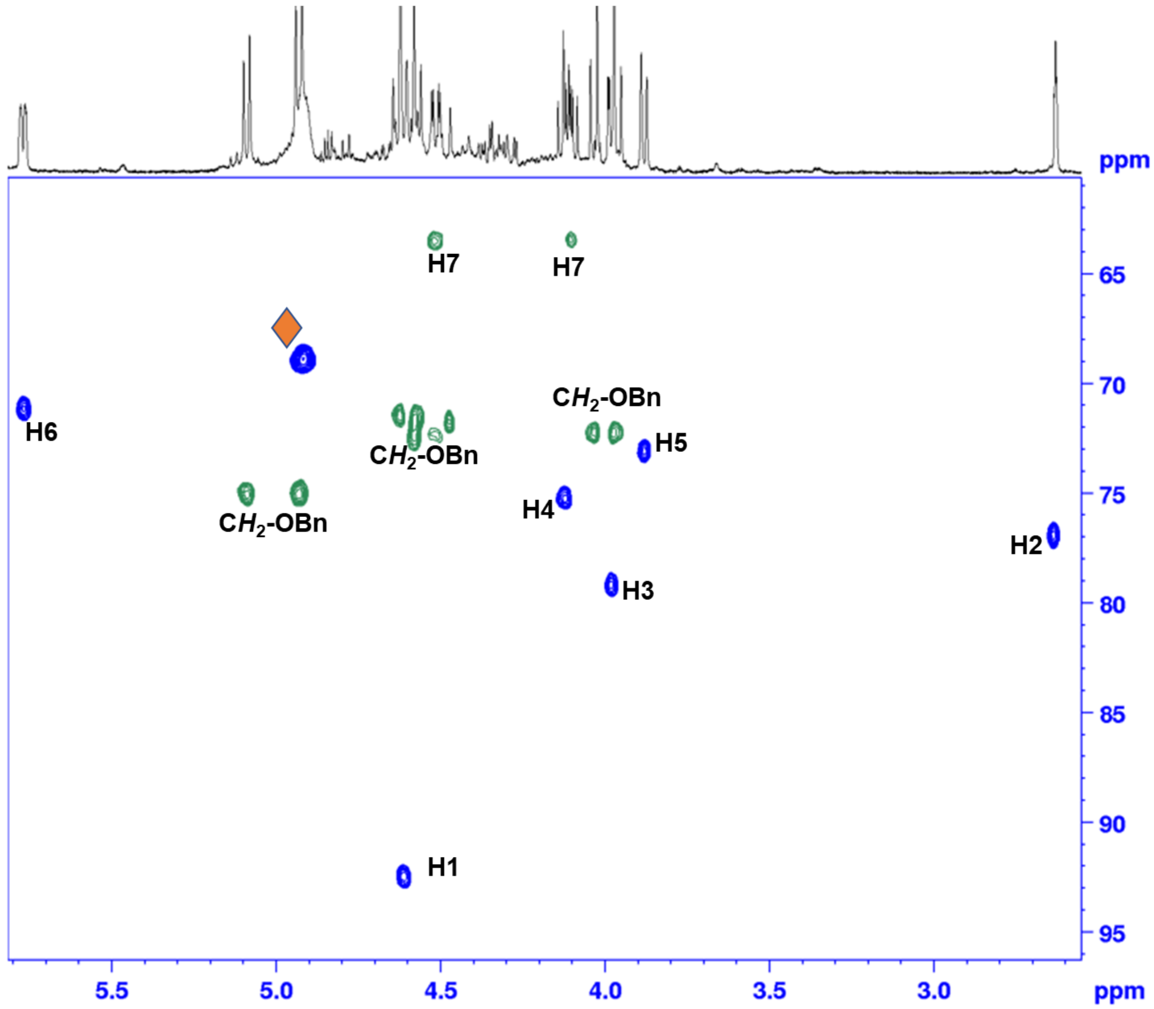

Supplement: Supplementary file 1 [file jo5c01760_si_001.pdf]
